# Deciduous pulp stem cell-derived extracellular vesicles stimulate the proliferation of cartilage progenitor cells via extracellular signal-regulated protein kinase 1/2 activation

**DOI:** 10.1038/s41598-026-37380-7

**Published:** 2026-03-09

**Authors:** Sara Murata, Soichiro Sonoda, Yukari Kyumoto-Nakamura, M. Majd Sharifa, Liting Yu, Reona Aijima, Erika Yamauchi-Tomoda, Fouad MHD. Zakaria, Hiroki Kato, Norihisa Uehara, Haruyoshi Yamaza, Takayoshi Yamaza

**Affiliations:** 1 Department of Molecular Cell Biology and Oral Anatomy, Kyushu University Graduate School of Dental Science, 3-1-1 Maidashi, Higashi-Ku, Fukuoka, 812-8582 Japan; 2 Department of Oral and Maxillofacial Surgery, Faculty of Medicine, Saga University Graduate School of Dental Science, Fukuoka, Japan; 3 Department of Oral and Maxillofacial Radiology, Kyushu University Graduate School of Dental Science, Fukuoka, Japan; 4 Laboratory of Creative Science for Insect Industries, Kyushu University Graduate School of Bioresource and Bioenvironmental Sciences, Fukuoka, Japan; 5 Department of Pediatric Dentistry, Graduate School of Medical and Dental Sciences, Kagoshima University, Kagoshima, 890-8544 Japan

**Keywords:** Cartilage progenitor cells, Extracellular vesicles, Stem cells from human exfoliated deciduous teeth, Extracellular signal-regulated kinase 1 and 2, Telomerase activity, G1/S phase transition, Cell biology, Molecular biology, Stem cells

## Abstract

The developmental dysfunction of cartilage progenitor cells (CPCs) causes dwarfism. Radical therapies for dwarfism remain underdeveloped. Recently, the therapeutic benefits of extracellular vesicles (EVs) released from human exfoliated deciduous tooth-derived stem cells (SHED) have been investigated for their potential to restore disease-target cells. However, the effects of EVs on human CPCs for the treatment of dwarfism remain unclear. We investigated the impact of EVs on cell proliferation, telomerase activity, *telomerase reverse transcriptase* (*TERT*) expression, cell cycle, and extracellular signal-regulated protein kinase 1/2 (ERK1/2) levels in human CPCs and in our established osteogenesis imperfecta (OI)-specific SHED (OI-SHED). EVs enhanced the proliferation and G1/S phase transition of CPCs, which was associated with increased *TERT* expression and telomerase activity. However, RNase-preconditioned EVs did not attenuate the efficacy of EVs in CPCs. ERK1/2 inhibitor tests demonstrated that CPCs exhibited suppressed proliferation, telomerase activity, and G1/S phase progression. EV-transferring CD29 induced ERK1/2 phosphorylation in CPCs, subsequently activating telomerase activity to induce proliferation. Interestingly, EV stimulation restored the reduction in cell proliferation, cell cycle progression, phosphorylated ERK1/2 levels, and *TERT* expression in OI-SHED. In conclusion, the present findings provide new insights into ERK1/2-mediated proliferation of human CPCs using EVs.

**Supplementary Information:**

The online version contains supplementary material available at 10.1038/s41598-026-37380-7.

## Introduction

Normal growth and development of skeletal tissues are essential for human life and are regulated by intramembranous and endochondral ossification^1^. The latter process occurs in the long bones of the body, such as the vertebrae, limb bones, and ribs. Growth plate cartilage progenitor cells (CPCs) are responsible for the longitudinal growth of long bones^2^.CPCs proliferate and form clonal longitudinal columns of proliferative chondrocytes. Subsequently, they differentiate into hypertrophic chondrocytes and undergo apoptosis. Some CPCs transdifferentiate into bone-forming osteoblasts beneath the growth plate. Clonal populations of CPCs have been identified in human fetal articular cartilage^3^.

Integrins are heterodimeric transmembrane proteins comprising alpha and beta subunits that bind to the extracellular matrix. The interaction between integrins and the extracellular matrix stimulates multiple mitogen-activated protein kinase families, such as extracellular signal-regulated protein kinase 1/2 (ERK1/2), to control cell proliferation and differentiation^4^. Chondrocyte-expressing CD29 (integrin beta 1) transduces various cartilaginous matrix molecules to promote cell proliferation via ERK1/2 signaling^5^.

The developmental dysregulation of chondrocyte proliferation causes dwarfism (short stature and limbs)^6^, for which radical therapies remain underdeveloped. Recently, the therapeutic benefits of mesenchymal stem cells (MSCs) have been proposed for the treatment of dwarfism^7,8^. Generally, multiple secretomes paracrine by parent MSCs play a role in the benefits of MSC-based therapies^9^. MSC-released extracellular vesicles act as intercellular cargos between donor and recipient cells and transfer bioactive molecules such as nucleic acids, soluble proteins, and membranous proteins^10–14^. Recently, we established extracellular vesicles from stem cells from human exfoliated deciduous teeth of healthy donors, referred to as EVs^9,10^, and reported their therapeutic potency to osteoporosis and autoimmune disease by targeting telomerase activity of the disease-site MSC-niche. Recent studies have engaged the epigenetic efficacy of EV-contained bioactive factors, such as nucleic acids and proteins, for treating cartilage repair and regeneration in cartilage disorders^15,16^. However, no prior studies have examined whether EVs on human cartilage progenitors would help frame the innovation.

In this study, we aimed to investigate the contribution of EVs to the proliferation performance of CPCs. We established human CPCs from a human fetal primary chondrocyte population to assess whether EV stimulation could induce the proliferation, population doubling levels, *telomerase reverse transcriptase* (*TERT*) expression, telomerase activity, and cell cycle machinery of CPCs. We also investigated intracellular ERK1/2 signaling in CPCs to determine the contribution of EVs to their proliferation. Furthermore, we show whether CD29 transferred from EVs triggers intracellular ERK1/2 signaling in CD29^–^CPCs. Finally, we established osteogenesis imperfecta (OI)-specific SHED (OI-SHED) and healthy donor-derived control SHED (CONT-SHED), which share CPC signatures, to analyze the effects of EVs on cell proliferation, cell cycle progression, phosphorylated ERK1/2 (pERK1/2) levels, and *TERT* expression of OI-SHED and CONT-SHED. Thus, we identified the roles of EVs in the activation of cell proliferation, telomerase activity, ERK1/2 phosphorylation, and G1/S progression.

## Results

### CPC-like cells were isolated from a primary human fetal chondrocyte population.

**Fig. 1:**
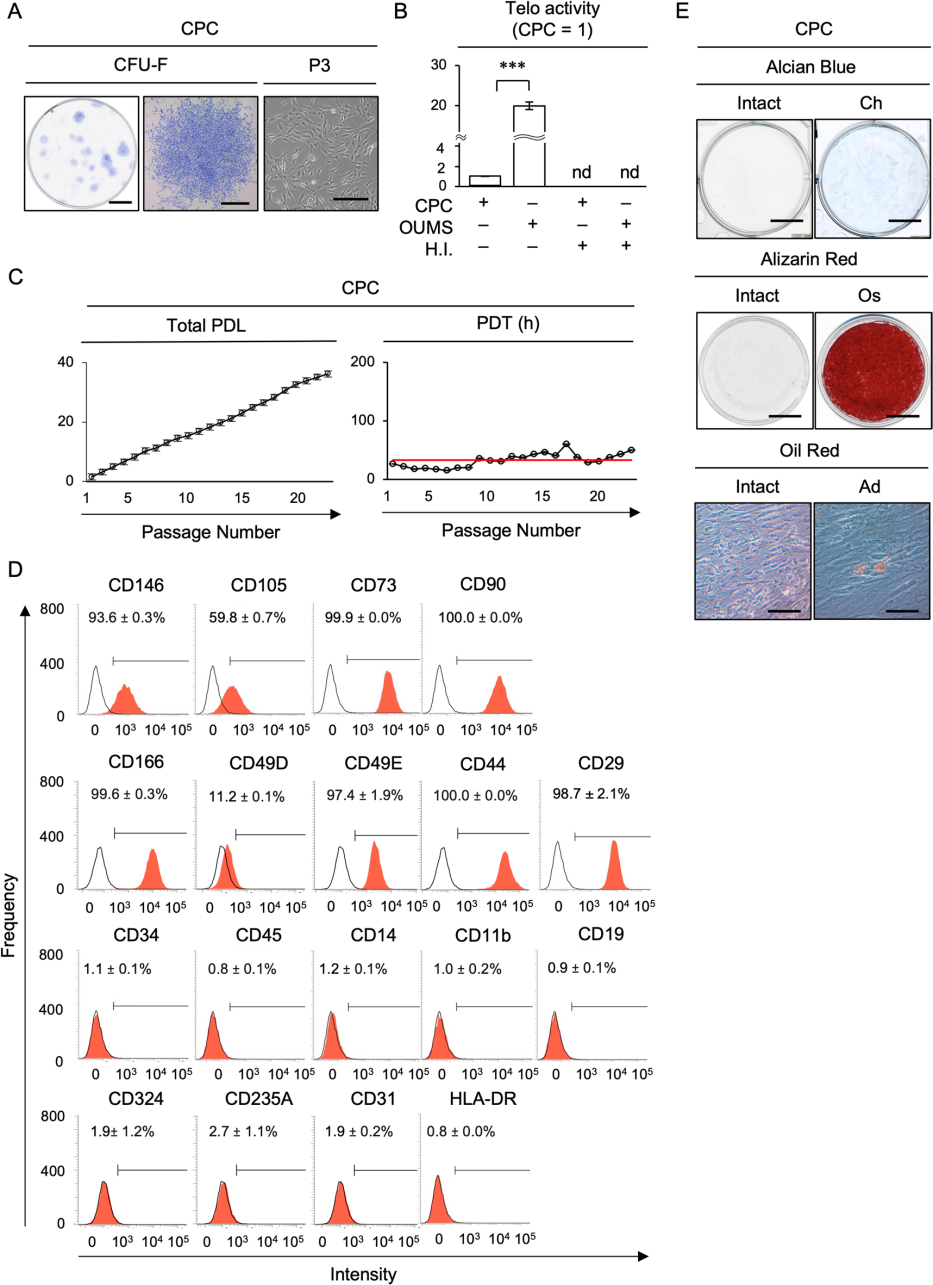
Characterization of CPCs from human fetal cartilage chondrocytes. (**A**) Representative images of CFU-F colonies on a dish (left image) and a CFU-F colony (middle image) of CPCs using toluidine blue staining. A representative microscopic image of passage 3 CPCs (P3, right image). Scale bar = 20 mm (left image), 500 μm (middle image), and 50 μm (right image). (**B**) A graph showing the results of telomerase activity (Telo activity) using TRAP-PCR. OUMS, human chondrosarcoma cell line OUMS-27 cells. H.I., heat inactivation pretreatment. The results are shown as a ratio to the result of CPCs (CPC = 1). (**C**) Graphs showing the results of total PDL and PDT in CPCs until passage 23. Red lines indicate the average of PDT. (**D**) Representative histograms of CPC markers using flow cytometry. Areas filled with red color, histograms stained with target antibodies; solid lines, histograms stained with isotype-matched control antibodies. Positive ratio (%) of cell surface markers are shown. (**E**) Representative images of cartilaginous matrix formation, calcified matrix deposition, and lipid accumulation for chondrogenic (Ch), osteogenic (Os), and adipogenic (Ad) potency using Alcian blue, Alizarin red, and Oil red staining. Scale bar = 20 mm (Alcian blue and Alizarin red staining), 100 μm (Oil red staining). Intact, each uninduced condition. (**B**–**D**) Data are presented as mean ± SD. n = 3/group. Significance was determined using an independent two-tailed *t*-test; ***P < 0.005. nd, not detectable.

Several studies have accumulated the utility of commercially available primary human fetal chondrocytes used in this study as a gold standard for investigating cartilage biology, pathology, and tissue engineering^17,18^. CPCs were isolated from the primary human fetal chondrocyte population using the colony-forming unit-fibroblasts (CFU-F) method. The isolated cells formed adherent cell clusters, which consisted of spindle-shaped cells, on a plastic culture dish (Fig. 1A). We expanded these cells to obtain passage 3 CPCs. The passage 3 CPCs displayed a low level of telomerase activity and high proliferative potency, as detected by telomerase repeat amplification protocol and polymerase chain reaction (TRAP-PCR) and population doubling assays (Fig. 1B, C). These cells showed an immunophenotype positive for CD166, CD146, CD105, CD73, CD90, CD44, CD49D, CD49E, and CD29, and negative for CD34, CD45, CD14, CD11b, CD19, CD235A, CD324, CD235A, CD31, and human leukocyte antigen DR (HLA-DR), as detected by flow cytometry (Fig. 1D). They exhibited multipotency into chondrocytes, osteoblasts, and adipocytes, as detected by tissue-specific staining, including Alcian blue, Oil red, and Alizarin red staining (Fig. 1E) and lineage-specific gene expression, including *SRY-box 9* (*SOX9*), *aggrecan* (*ACAN*), *collagen type II alpha 1 chain* (*COL2A1*), and *collagen type X alpha 1 chain* (*COL10A1*) specific for chondrocytes, *Runt-related transcription factor 2* (*RUNX2*) and *bone gamma-carboxyglutamate protein* (*BGLAP*) specific for osteoblasts, and *peroxisome proliferator-activated receptor gamma* (*PPARG*) and *lipoprotein lipase* (*LPL*) specific for adipocytes, using quantitative reverse transcription polymerase chain reaction (RT-qPCR) (Supplementary Fig. 1). These findings indicate that CFU-F-forming chondrocytes exhibit typical CPC-like features^11^.

### EV contents other than RNA activate telomerase activity, cell proliferation, and cell cycle of CPCs

We isolated CONT-SHED along with MSC features, including colony formation, MSC-like immunophenotype, and multipotency into adipocytes, chondrocytes, and osteoblasts (Supplementary Fig. 2). We analyzed the effects of conditioned medium of SHED (CM) on the viability and proliferation of CPCs using highly water-soluble tetrazolium salt 8 (WST8) and 5-bromo-2’-deoxyuridine (BrdU) incorporation assays. CM loading increased the viability and proliferation of CPCs compared to phosphate-buffered saline (PBS; Mock) loading on days 3 and 5 after treatment (Supplementary Fig. 3). We examined whether the loading effects of CM were dependent on EV-containing factors. EVs were depleted from CM using membrane affinity spin columns. EV-depleted CM loading did not induce cell viability or proliferation of CPCs on day 3 (Supplementary Fig. 4).

**Fig. 2:**
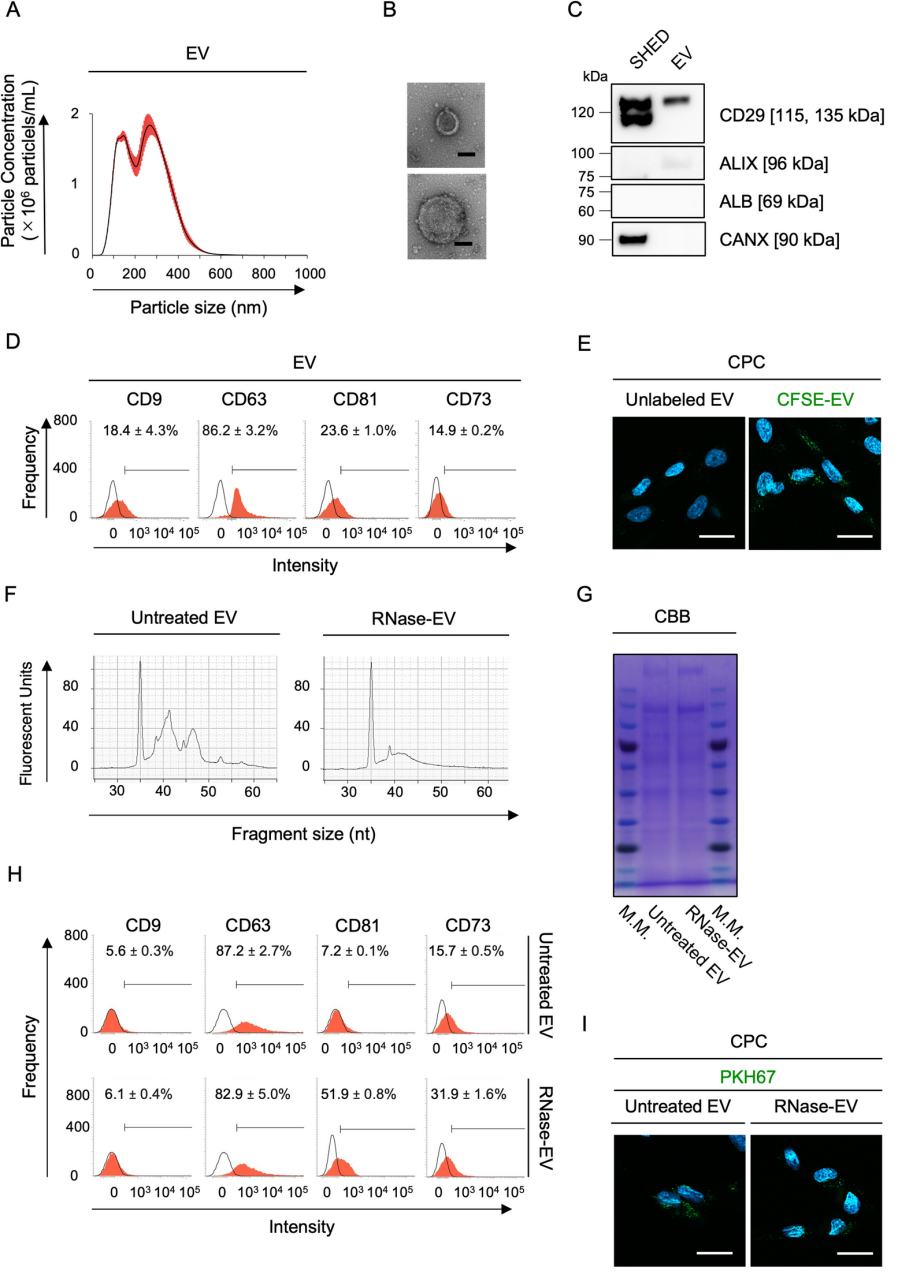
Characterization of SHED-derived EVs. (**A**) Representative histogram of particle size of EVs by nano-tracking particle analysis. (**B**) Representative transmission electron micrographs of small (upper image) and middle to large-sized (lower image) EVs. Negative staining. (**C**) Representative immunoblot images of CD29, ALIX, ALB, and CANX levels in CONT-SHED and EVs. (**D**) Representative histograms of CD9, CD63, CD81, and CD73 levels in EVs using flow cytometry. (**E**) Representative fluorescent images of the internalization of CSFE-labelled EVs and unlabeled EVs in CPCs 10 min after loading. (F) Graphs showing the results of RNA contents in RNase-treated EVs and untreated EVs. (**G**) A representative gel image of total protein contents. CBB staining. M.M., molecular marker. **(H**) Representative histograms of CD9, CD63, CD81, and CD73 levels using flow cytometry. (**I**) Representative fluorescent images of the internalization of PKH67-labeled RNase-treated EVs and untreated EVs in CPCs 10 min after loading. (**B**, **E**, **I**) Scale bars = 50 nm (**B**), 10 μm (**E**, **I**). (**D**, **H**) Areas filled with red color, histograms stained with target antibodies; solid lines, histograms stained with isotype-matched control antibodies. n = 3/group. Positive ratios (%) of markers are shown. Data are presented as mean ± SD. (E, I) DAPI counterstaining.

EVs were isolated from CM using membrane-affinity spin columns. According to nanoparticle tracking particle analysis, EVs ranged in size from 50 to 200 nm and 250 to 700 nm in diameter (Fig. 2A). Both EV sizes contained an electron-dense, core-like matrix surrounded by an electron-lucent, halo-like matrix encircled by a bilayer membrane, as detected by transmission electron microscopy (Fig. 2B). EVs expressed CD29 and apoptosis-linked gene-2 interacting protein X (ALIX) but lacked albumin (ALB) and endoplasmic reticulum-derived calnexin (CANX) compared to parental CONT-SHED, as determined by immunoblotting (Fig. 2C). They also expressed CD9, CD63, CD81, and CD73, as determined by flow cytometry (Fig. 2D). The loading of carboxyfluorescein succinimidyl ester (CFSE)-labeled EVs detected fluorescence in the CPCs, but that of PBS-treated unlabeled EVs did not 10 min after loading (Fig. 2E). CPCs were treated with EVs (4, 20, and 40 μg/mL) or PBS (Mock), and CPC proliferation was analyzed 3 days after stimulation using the BrdU incorporation assay. EV-stimulated CPCs enhanced cell proliferation in a dose-dependent manner compared to untreated CPCs (Supplementary Fig. 5). Therefore, we decided to use 4 μg/mL of EVs for further experiments.

Enriched small RNAs, mostly microRNAs (miRNAs), in stem cell-derived EVs facilitate cell–cell communication^12^. To understand the role of RNA in EVs in cell–cell communication, we established RNA-deleted EVs preconditioned with ribonuclease A (5 U/mL; RNase) or PBS (Mock), RNase-treated EVs, and untreated EVs for 30 min. RNase preconditioning effectively reduced the RNA content in RNase-treated EVs compared to untreated EVs (Fig. 2F). However, RNase preconditioning did not affect the total protein content between RNase-treated EVs and untreated EVs, as determined by Coomassie brilliant blue (CBB) staining (Fig. 2G). The total protein concentration was 2.2 ± 0.1 × 10^3^ and 3.1 ± 0.2 × 10^3^ μg/mL in RNase-treated EVs and untreated EVs (mean ± SD; n = 3 each), respectively. RNase-treated EVs showed immunophenotypes and internalization potency similar to those of untreated EVs, as determined using flow cytometry and the PKH67-labeling membrane tracking assay (Fig. 2H, I).

**Fig. 3:**
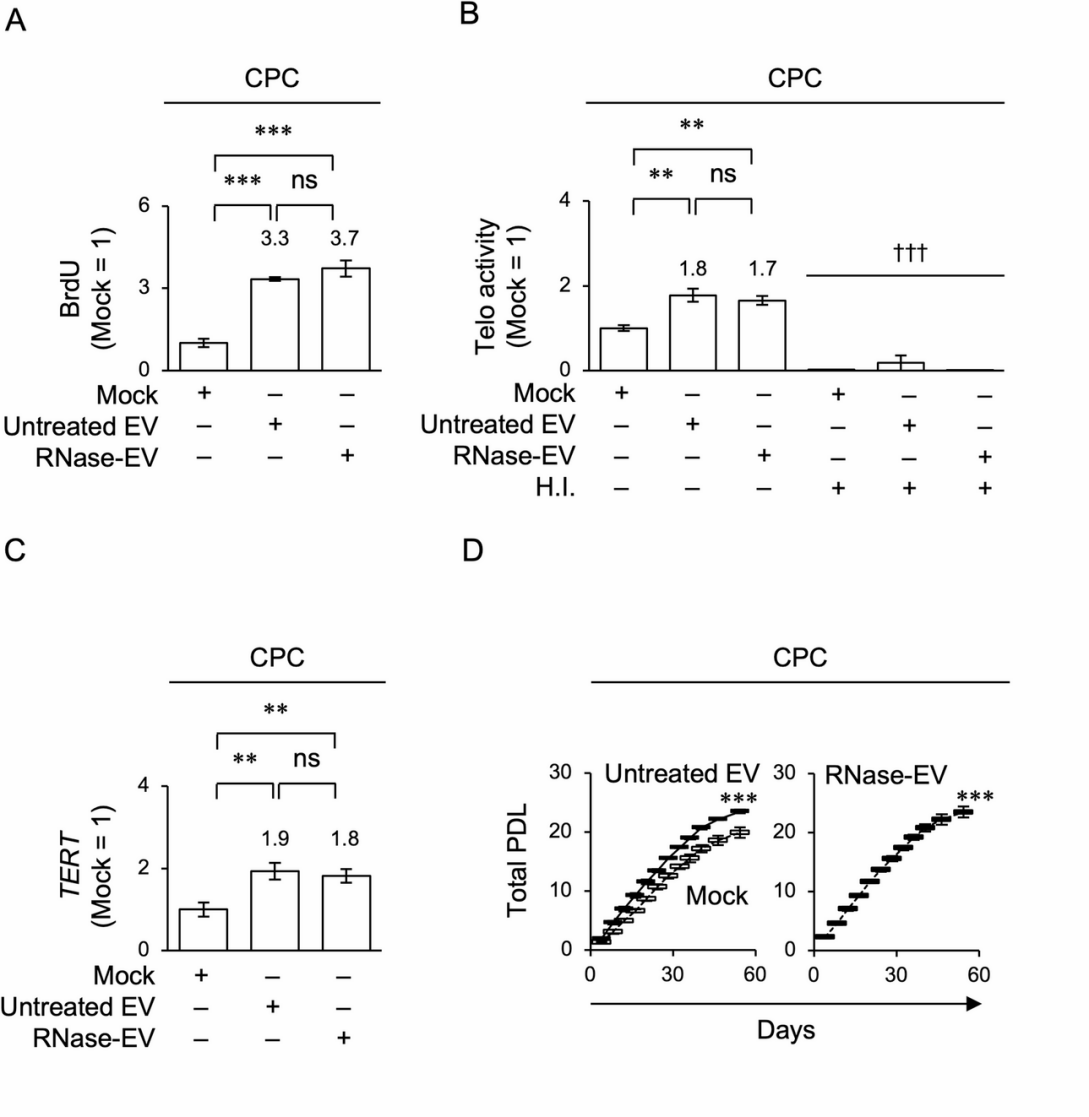
Effects of EVs on cell proliferation, telomerase activity, *TERT* expression, and population doubling of human CPCs. (A–F) CPCs were treated with PBS (Mock), PBS-treated EVs (untreated EVs) (4 μg/mL), and RNase-treated EVs (4 μg/mL). Graphs showing the results of cell proliferation using BrdU incorporation assay (**A**), telomerase activity (Telo activity) using TRAP-PCR (**B**), *TERT* expression using RT-qPCR (**C**), and total PDL (**D**). H.I., heat inactivation (**B**). Data are analyzed as the ratio of 18S rRNA expression (**C**). Representative contour plots of the cell cycle in CPCs assessed using flow cytometry with BrdU-7AAD staining (**E**). Graphs showing the percentage (%) of the G0/G1, S, and G2/M phases in CPCs using flow cytometry (**F**). (**A**–**D**, **F**) n = 3/group. Data are presented as mean ± SD. Significance was determined using a one-way ANOVA with Tukey’s post-hoc test. *P < 0.05, **P < 0.01, ***P < 0.005. ns, not significant. †††* P* < 0.005 versus the corresponding sample group. (**A**–**C**) Results are shown as a ratio of the results of untreated CPCs (Mock = 1). The numbers indicate the average ratio to untreated CPCs.

EV-loaded CPCs exhibited enhanced BrdU uptake, telomerase activity, *TERT* expression, and population doubling levels (PDL) compared to Mock-loaded CPCs (untreated CPCs) using BrdU incorporation, TRAP-PCR, RT-qPCR, and population doubling assays (Fig. 3A–D). EV-loaded CPCs displayed significant alterations in DNA content 24 h after EV loading, as determined by flow cytometry with propidium iodide staining (Supplementary Fig. 6). Further flow cytometry with BrdU and 7-amino-actinomycin D (BrdU-7AAD) staining demonstrated that EV-loaded CPCs showed a shortened G0/G1 phase and prolonged S and G2/M phases compared with untreated CPCs 24 h after loading (Supplementary Fig. 7). In contrast, RNase-treated EV loading did not attenuate the enhanced effects on cell proliferation, telomerase activity, *TERT* expression, PDL, or the accelerated G1/S transition of CPCs under untreated EV loading (Fig. 3 and Supplementary Fig. 7). Considering the results of the PKH67-labeling membrane tracking assay, the present findings from RNase-treated EV loading tests suggest that EV contents other than RNA, such as membrane proteins, participate in the cellular functions of EV on CPCs.

### EVs loading stimulates phosphorylation of ERK1/2 to control telomerase activity, G1/S transition, and cell proliferation in CPCs

**Fig. 4:**
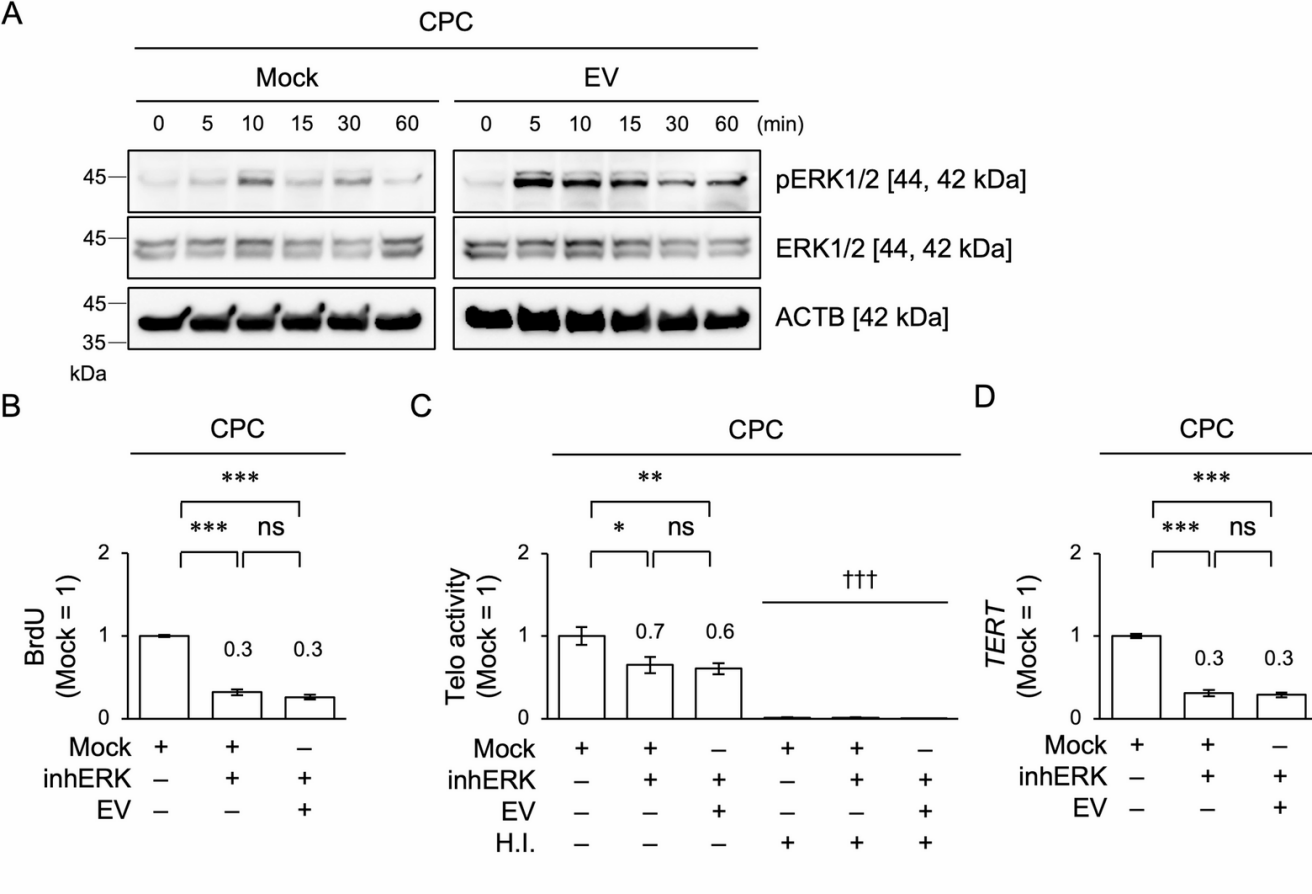
Effects of ERK1/2 inhibitor on the cell proliferation, telomerase activity, and *TERT* expression in CPCs under EV stimulation. (**A**) CPCs were loaded with EVs or Mock (PBS) for 0, 5, 10, 15, 30, and 60 min. Representative immunoblot images of the temporal levels of ERK1/2 and pERK1/2 in CPCs. ACTB, actin beta. (**B**–**D**) CPCs were pretreated with an ERK1/2 inhibitor, PD98059 (inhERK; 10 μM) or Mock (PBS), for 1 day and subsequently treated with EVs (4 μg/mL). Graphs showing the results of cell proliferation using BrdU incorporation assay (**B**), telomerase activity (Telo activity) using TRAP-PCR (**C**), and *TERT* expression using RT-qPCR (**D**). H.I., heat inactivation (**C**). Data are analyzed as a ratio to 18S rRNA expression (**D**). (**B**–**D**) n = 3/group. Data are presented as mean ± SD. The results are shown as a ratio to the result of untreated CPCs (Mock = 1). The numbers indicate the average ratio to untreated CPCs. Significance was determined using a two-tailed t-test; ***P < 0.005. †††* P* < 0.005 versus the corresponding sample group. ns, not significant.

Various cartilaginous matrices stimulate chondrocyte proliferation via integrin-ERK1/2 signaling^5^. To understand the effects of EVs on intracellular signaling in CPCs, we investigated the effects of EV loading on the temporal levels of intracellular ERK1/2 signaling in CPCs using immunoblotting. EV loading increased pERK1/2 levels at 5 min and sustainably maintained the increased levels up to 60 min (Fig. 4A and Supplementary Fig. 8) compared to Mock loading. To analyze the dominance of ERK1/2 activity after EV loading, CPCs were pretreated with an ERK1/2 inhibitor, PD98059 (10 μM; inhERK), or dimethyl sulfoxide (Mock) for 1 day and compared with pretreated CPCs. The inhERK-pretreated CPCs exhibited a deficiency in cell proliferation, *TERT* expression, and telomerase activity compared with untreated CPCs (Figs. 4B–D). The inhERK-pretreated CPCs exhibited a decrease in G1/S transition compared to untreated CPCs (Supplementary Fig. 9). EV loading did not restore the cellular deficiency in inhERK-pretreated CPCs to untreated CPCs (Fig. 4B–D and Supplementary Fig. 9), indicating that ERK1/2 activity is a crucial signal transduction pathway for understanding the effects of EV loading on enhancing proliferation in CPCs with increased telomerase activity and progressed G1/S transition.

### EV-transferring CD29 is involved in the activation of intracellular ERK1/2, telomerase activity, cell cycle, and cell proliferation signaling in CPCs

**Fig. 5:**
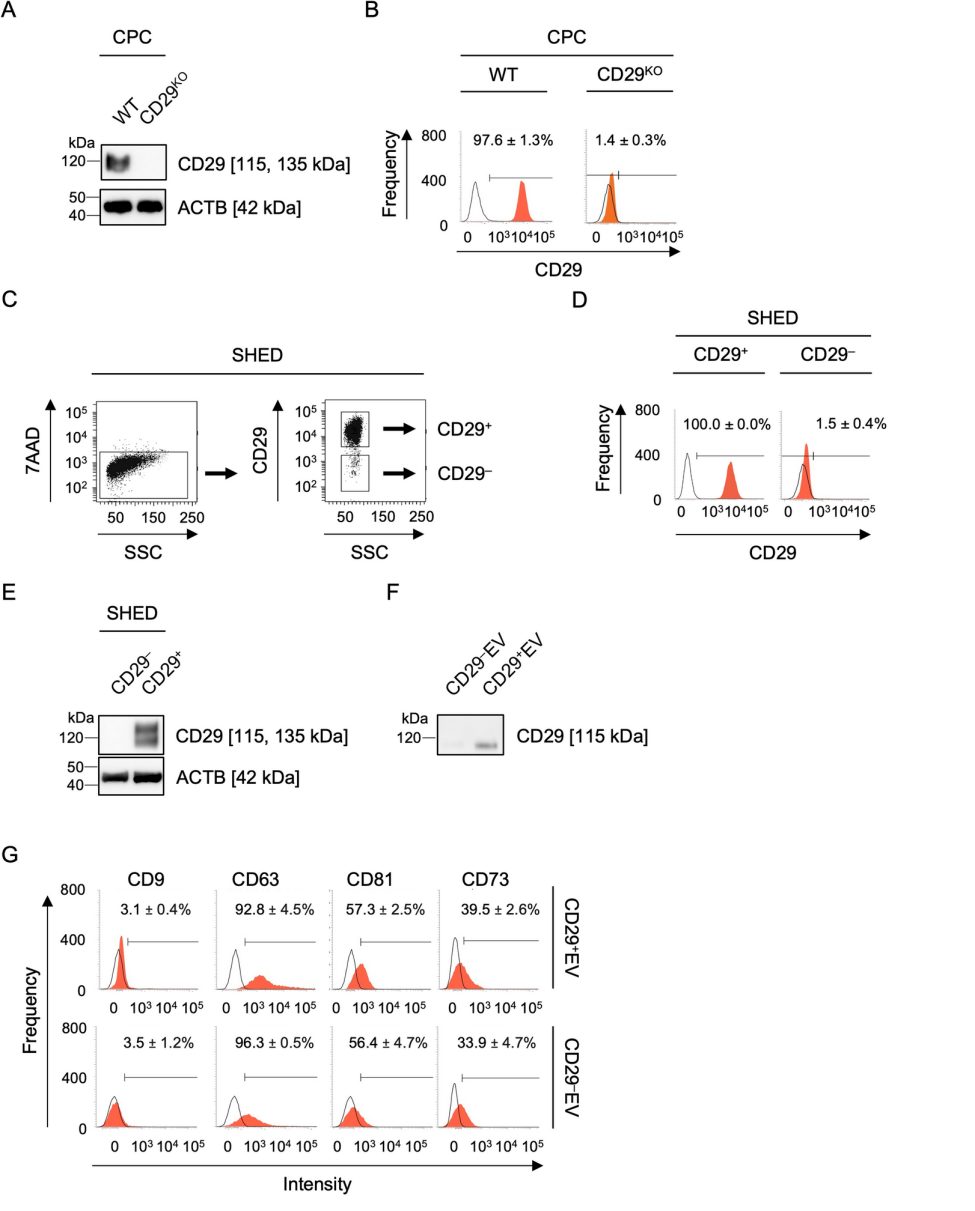
Establishment of CD29^KO^CPCs and isolation of CD29^+^EVs and CD29^–^EVs. (**A**, **B**) Representative immunoblot images (**A**) and histograms (**B**) of CD29 levels in CD29^KO^CPCs (CD29^KO^) and wild-type CPCs (WT). (**C**) Representative dot plots of CD29^+^ and CD29^–^population purified from CONT-SHED using fluorescent cell sorting. SSC, side scatter light. (**D**, **E**) Representative histograms (**D**) and immunoblot images (**E**) of CD29 levels in purified CD29^+^ and CD29^–^ population in CONT-SHED. (**F**, **G**) Representative immunoblot images of CD29 levels (F) and histograms of extracellular vesicle marker expression (**G**) in CD29^+^EVs and CD29^–^EVs. (A, E) ACTB, actin B. (B, D, G) n = 3/group. Positive ratios (%) of markers are shown. Areas filled with red color, histograms stained with target antibodies; solid lines, histograms stained with isotype-matched control antibodies. Data are presented as mean ± SD.

**Fig. 6:**
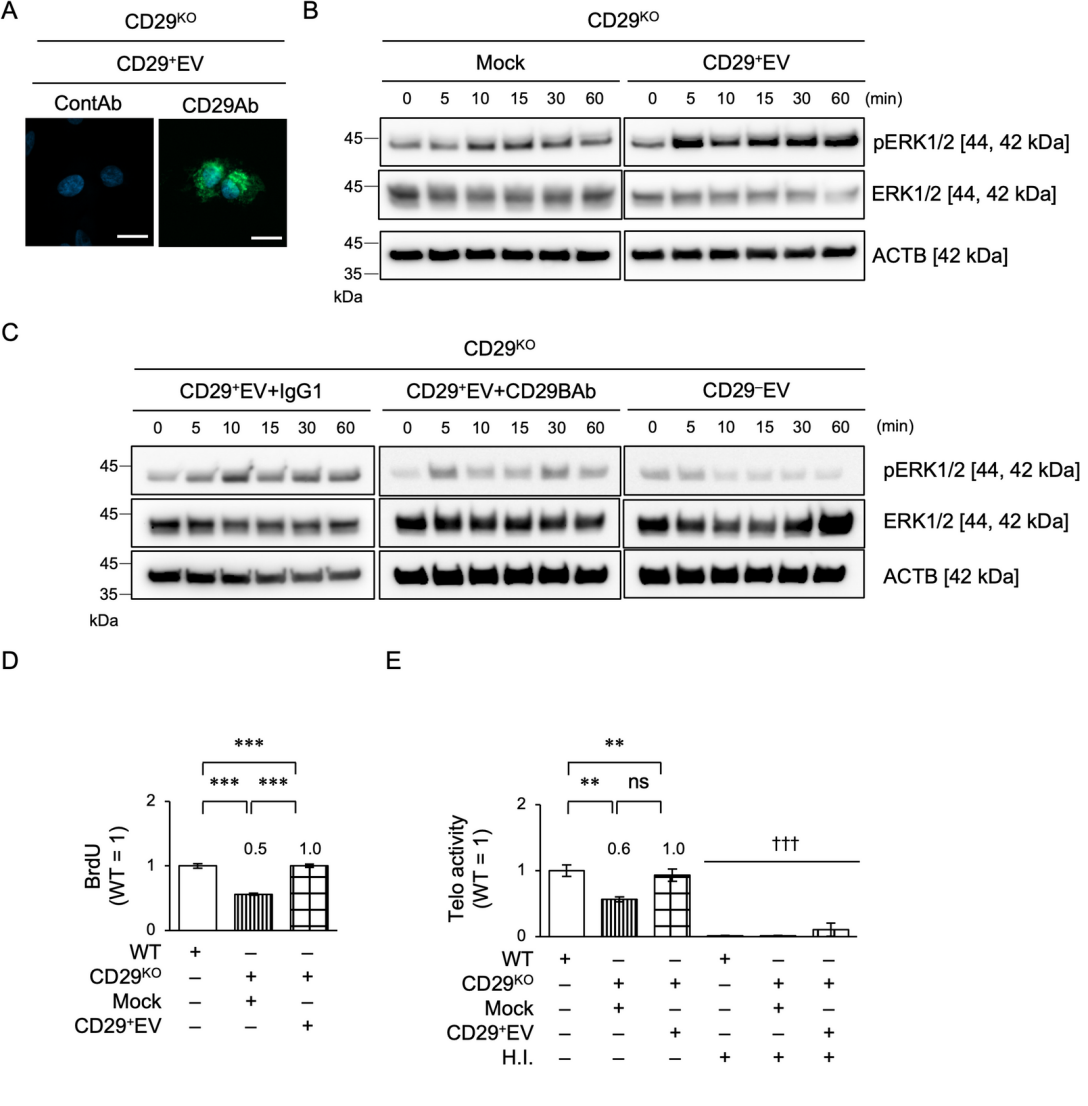
Effects of EV-transferring CD29 on the ERK phosphorylation, cell proliferation, and telomerase activity in CPCs under EV stimulation. EVs (4 μg/mL) were loaded on CD29^KO^CPCs (CD29^KO^). (**A**) Representative fluorescent images of membranous CD29 on CD29^+^EVs in CD29^KO^CPCs 10 min after CD29^+^EV loading. DAPI counterstaining. ContAb, Alexa Fluor488-conjugated control antibody-labeling; CD29Ab, Alexa Fluor488-conjugated anti-ITGB1 antibody-labeling. Scale bars = 10 μm. (**B**–**E**) Representative immunoblot images of the temporal levels of ERK1/2 and pERK1/2 in CD29^KO^CPCs. ACTB, actin beta. Mock, PBS loading; CD29^+^EV, CD29^+^EV loading; CD29^–^EV, CD29^–^EV loading; IgG1, IgG1 loading; CD20BAb, anti-CD20 blocking loading (**B**, **C**). Graphs showing the results of cell proliferation using BrdU incorporation assay (**D**) and telomerase activity (Telo activity) using TRAP-PCR in CD29^KO^CPCs (**E**). H.I., heat inactivation (**E**). (**D**, **E**) n = 3/group. Data are presented as mean ± SD. The results are shown as a ratio to the result of wild-type CPCs (WT = 1). The numbers indicate the average ratio to wild-type CPCs. Significance was determined using a two-tailed t-test; **P < 0.01, *P* < 0.005. †††* P* < 0.005 versus the corresponding sample group. ns, not significant.

Next, we examined the effects of transferring membranous proteins from donor EVs to recipient CPCs. We focused on CD29 because of its expression on EVs and its ability to promote chondrocyte proliferation via ERK1/2 through CD29^5^. We generated *CD29*-edited CPCs using the lentiviral clustered regularly interspaced short palindromic repeat/CRISPR-associated protein 9 (CRISPR/Cas9) system and drug screening, and cloned *CD29*-knockout CPCs (CD29^KO^CPCs) and their control wild-type CPCs (Fig. 5A, B). We also purified CD29^+^ and CD29^–^ populations from CONT-SHED using fluorescent cell sorting (Figs. 5C–E) and extracted CD29^+^ population- and CD29^–^ population-derived EVs, CD29^+^EVs and CD29^–^EVs (Figs. 5F, G). When Alexa Fluor488-conjugated anti-CD29 antibody-labeled CD29^+^EVs were loaded, fluorescence was detected in CD29^KO^CPCs 10 min after loading, but not in control antibody-labeled CD29^+^EVs (Fig. 6A). CD29^+^EV loading increased pERK1/2 levels at 5 min and sustainably maintained these levels up to 60 min, but Mock loading did not, in CD29^KO^CPCs, as detected by immunoblotting (Fig. 6B and Supplementary Fig. 10). Anti-CD29 blocking antibodies reduced the pERK1/2 levels in CD29^KO^CPCs under CD29^+^EV loading compared to their control antibodies (Fig. 6C and Supplementary Fig. 11). CD29^–^EV loading also decreased the pERK1/2 levels in CD29^KO^CPCs(Fig. 6C and Supplementary Fig. 11). BrdU incorporation assay, TRAP-PCR, and flow cytometry with BrdU-7AAD staining showed impaired cellular functions of proliferative capacity, telomerase activity, and G1/S phase transition in CD29^KO^CPCs compared with those in wild-type CPCs (Figs. 6D, E, and Supplementary Fig. 12). Meanwhile, CD29^+^EV loading ameliorated the deficiency in CD29^KO^CPCs (Figs. 6D, E, and Supplementary Fig. 12). These findings suggest that EVs can directly transfer donor membrane proteins and activate endogenous ERK1/2 signaling in recipient CPCs.

### EV loadings stimulate proliferation and pERK1/2 levels in chondrocytes of the cartilaginous growth plate

We examined whether EV loading stimulated chondrocyte proliferation and increased pERK1/2 levels in the growth plate cartilage of newborn mouse tibiae ex vivo. The growth plate cartilage consisted of resting, proliferating, and hypertrophic zones (Supplementary Fig. 13A). Immunohistochemical analysis revealed that EV loading increased BrdU-positive nuclei and enhanced pERK1/2 levels in the chondrocytes of the proliferating zone compared to mock loading (Supplementary Fig. 13B, C), indicating that EVs have the potential to accelerate cell division in the growth plate chondrocytes of developing long bones.

### EV loading stimulates phosphorylation of ERK1/2 to control cell proliferation, telomerase activity, and G1/S transition in OI-SHED.

**Fig. 7:**
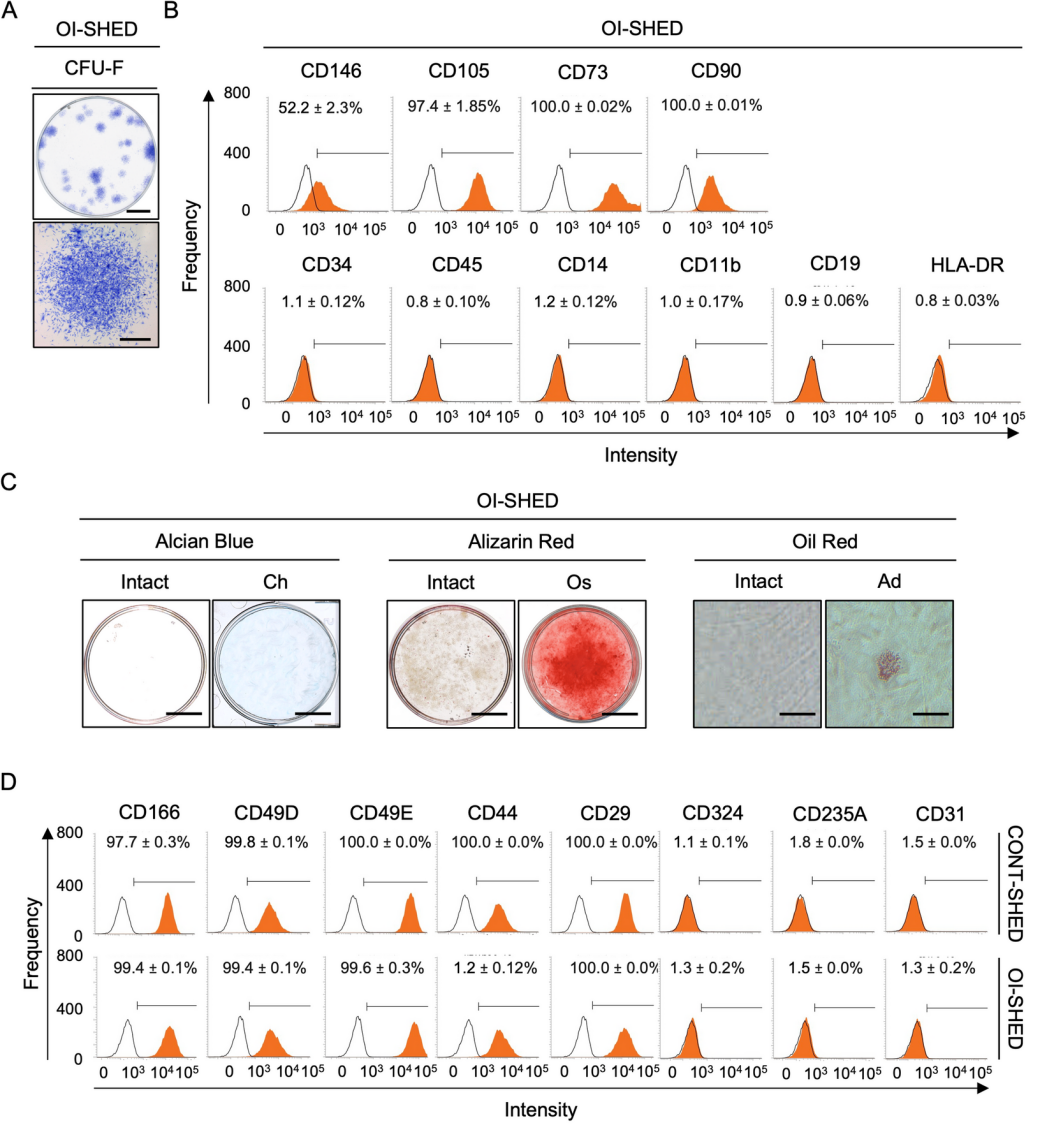
Characterization of OI-SHED. OI-SHED were isolated from patients (n = 2) with OI type III using the CFU-F method. (**A**) Representative images of CFU-F colonies on a whole dish (upper image) and a CFU-F colony (lower image). Bar, 20 mm (upper), 500 μm (lower). (**B**) Representative histograms of CD146, CD105, CD73, CD90, CD34, CD45, CD14, CD11b, CD19, and HLA-DR expression in OI-SHED using flow cytometry. (**C**) Representative images of cartilaginous matrix formation, calcified matrix deposition, and lipid accumulation in OI-SHED using Alcian blue, Alizarin red, and Oil red staining. Bar, 20 mm (Alcian blue staining and Alizarin red), 100 μm (Oil red staining). (D) Representative histograms of CD166, CD49D, CD49E, CD44, CD29, CD324, CD235A, and CD31 expression in OI-SHED and CONT-SHED using flow cytometry. (**B**, **D**) Areas filled with red color, histograms stained with target antibodies; solid lines, histograms stained with isotype-matched control antibodies. n = 3/group. The numbers indicate the positive ratio (%) of the cell surface. Data are presented as mean ± SD.

Generally, the patients with OI type III exhibit hereditary disorders of dentin formation, dentinogenesis imperfecta type I, expressing small pulp or pulp obliteration by X-ray analysis^19^. Finally, we established SHED from two patients with OI-SHED, which displayed CFU-F-forming ability and MSC immunophenotype (Fig. 7A, B). They exhibited multipotency into chondrocytes, osteoblasts, and adipocytes, as detected by tissue-specific staining, including Alcian blue, Oil red, and Alizarin red staining (Fig. 7C) and lineage-specific gene expression, including *SOX9*, *ACAN*, *COL2A1*, and *COL10A1* specific for chondrocytes, *RUNX2* and *BGLAP* specific for osteoblasts, and *PPARG* and *LPL* specific for adipocytes, using RT-qPCR (Supplementary Fig. 14).

In addition, OI-SHED and CONT-SHED shared CPC-like signatures, as indicated by CPC immunophenotypes positive for CD166, CD44, CD49D, CD49E, and CD29, and negative for CD235A, CD324, and CD31 (Fig. 7D), indicating that OI-SHED and CONT-SHED are considered to be CPC sources.

**Fig. 8:**
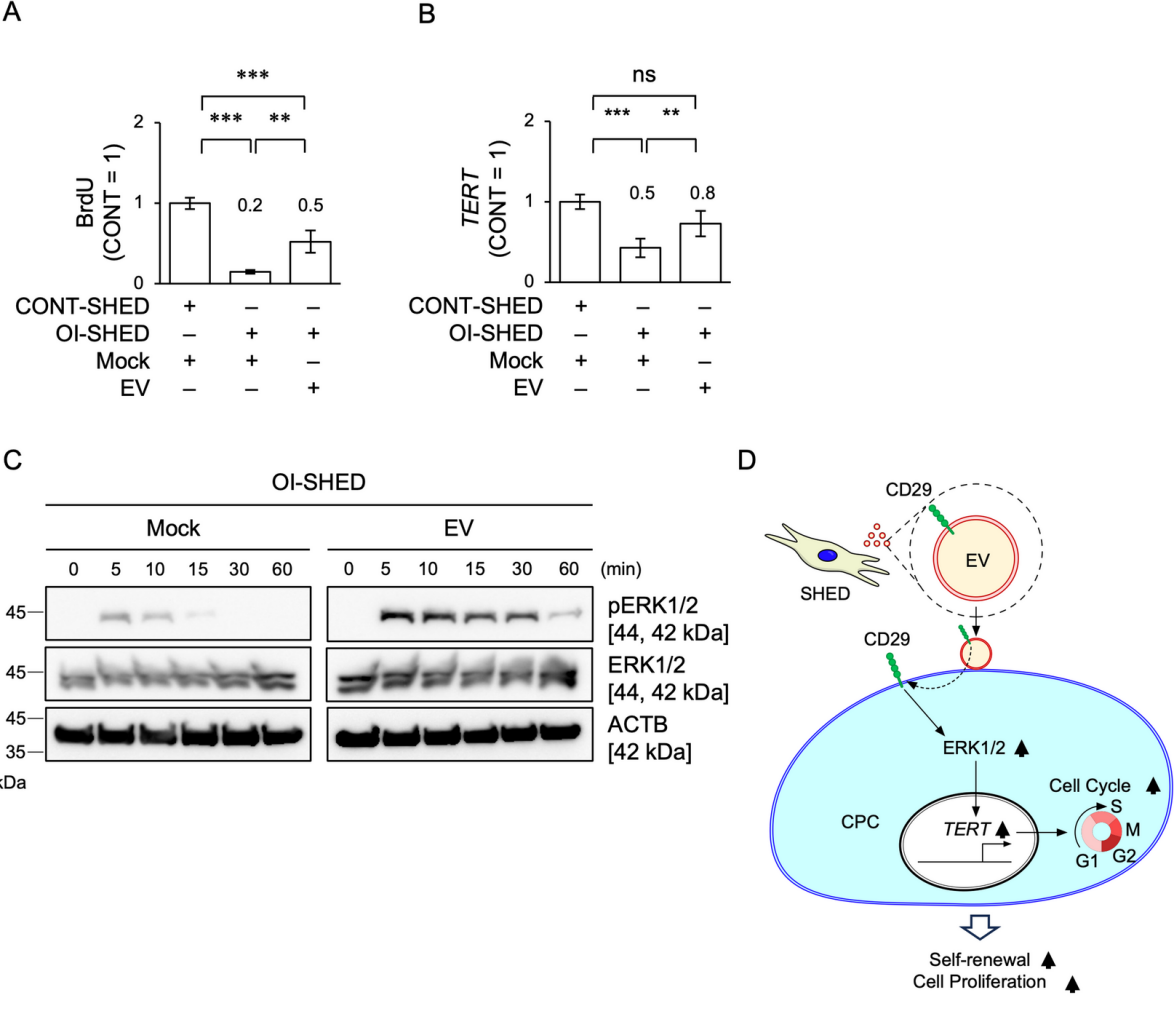
Effects of EVs on cell proliferation, *TERT* expression, and ERK1/2 phosphorylation of OI-SHED. OI-SHED were treated with PBS (Mock) and EVs (4 μg/mL). (**A**) Graphs showing the results of cell proliferation in OI-SHED on Day 3 after EV loading using BrdU incorporation assays. (**B**) A graph showing the results of *TERT* expression using RT-qPCR. Data are analyzed as the ratio of 18S rRNA expression. (**C**) Representative immunoblot images show the temporal levels of ERK1/2 and pERK1/2 in OI-SHED. ACTB, actin beta. (**D**) A graphic summary. This study demonstrates that the cell membrane expressing CD29 on SHED-releasing EVs transfers to the cell surface membrane of human CPCs. The transferred CD29 enhances telomerase activity and progresses the G1/S phase transition by activating ERK1/12-mediated *TERT* expression in human CPCs, thereby inducing the self-renewal and proliferation. (A, B) n = 3/group. Data are presented as mean ± SD. Results are shown as a ratio of the results of Mock-treated CONT-SHED (CONT = 1). The numbers indicate the average ratio to OI-SHED. Significance was determined using a one-way ANOVA with Tukey’s post-hoc test. ** < 0.01, ***P < 0.005. ns, not significant.

OI-SHED displayed decreased cell proliferation capacity, as indicated by reduced BrdU uptake and a delayed cell cycle with increased G0/G1 and reduced S and G2/M phases compared to CONT-SHED, using BrdU incorporation assay and flow cytometry with BrdU-7AAD staining (Figs. 8A and Supplementary Fig. 15). EV loading restored the decreased proliferation capacity of OI-SHED (Figs. 8A and Supplementary Fig. 15). EV loading restored the reduced *TERT* expression in OI-SHED compared to that in CONT-SHED (Fig. 8B). EV loading increased pERK1/2 at 5 min and sustainably maintained the increased levels up to 30 min after stimulation in OI-SHED (Fig. 8C and Supplementary Fig. 16).

## Discussion

Extracellular vesicle-containing miRNAs participate in the cellular functions of target cells; MicroRNA-197 and miR-26a-5p in extracellular vesicles increase chondrocyte proliferation^13,14^. SHED-released EV-containing MIR346 accelerates *TERT* expression and telomerase activity in target MSCs^9,10^. However, the present EV-mediated acceleration of CPC proliferation was independent of the RNA content of EVs. The present findings in CD29^+^EV-loaded CD29-knockout CPCs suggest the involvement of membranous proteins of EVs in recipient CPC proliferation. Membranous proteins of MSC-derived extracellular vesicles, including CD73, CD95, and CD274, mediate the action of MSCs on recipient cells^20–22^. Our findings indicate that EV-activated ERK1/2 is a crucial signaling pathway in recipient human CPC proliferation associated with telomerase activity and G1/S phase progression. Several types of receptors, including fibroblast growth factor receptors, parathyroid hormone-related peptide receptors, and transforming growth factor-beta receptors, are expressed on chondrocytes and induce chondrocyte proliferation via ERK1/2 activation^23^. The crucial role of ERK1/2-regulating Indian hedgehog mRNA in CFK-2 chondrocyte cell-lined cells indicates the induction of chondrocyte proliferation via Indian hedgehog in a paracrine or autocrine manner^24^. Further studies are necessary to identify the crucial membranous factor(s) of EVs that trigger ERK1/2 activation, increasing the cellular kinetics of telomerase activity and G1/S transition in human CPCs.

Cell cycle progression in growth plate chondrocytes is responsible for the longitudinal growth of endochondral bones^25^. The regulation of cyclin D1 (CCND1) is crucial for the G1/S transition^26^. In the growth plate cartilage, CCND1 expression is limited to proliferating chondrocytes^27^. CCND1-deficient mice exhibit a reduced proliferation zone^25^. ERK1/2 controls proliferation by inducing CCND1-mediated G1/S transition in embryonic stem cells^26^. Chondrocytes in *CD29*-deficient mice exhibit a deletion of the G1/S transition associated with pERK1/2 reduction^28^. Our results using ERK1/2 inhibitors suggest that EV-induced ERK1/2 signaling plays a significant role in regulating the G1/S cell cycle transition in human CPCs. Further studies are necessary to elucidate the molecular mechanisms underlying the G1/S transition in human CPCs via EV-induced ERK1/2 signaling.

Human TERT (hTERT)-regulated telomerase activity contributes to self-renewal capacity, clonogenicity, and proliferation of tissue stem/progenitor cells^29,30^. The ERK1/2 signaling pathway activates the hTERT promoter to increase telomerase activity^31,32^. Ectopic stable expression of hTERT accelerates proliferation capacity and prolongs the replicative lifespan in human and canine chondrocytes^33,34^. hTERT mRNA expression is significantly observed in the proliferating and hypertrophied zones compared to the resting and calcified zones of the growth plates of articular cartilage, particularly at human developmental stages from fetus to child^35^. The MEK/ERK signaling activation participates in the proliferation of rabbit chondrocytes by statin^36^. MSC-derived extracellular vesicles increase chondrocyte proliferation through the ERK1/2 signaling pathway^22^. In the present study, EV loading activated the ERK1/2 signaling pathway, *TERT* expression, telomerase activity, and the G1/S cell cycle transition in CPCs. Given that the self-renewal of human embryonic stem cells is associated with shortened G1 phase, regulated by G1-related cyclin D2 and cyclin-dependent kinase 4^37^, EV-induced G1/S cell cycle transition supports the upregulation of self-renewal capacity in CPCs. These findings suggested that EV-stimulated ERK1/2 controls self-renewal and proliferation of CPCs through the hTERT-mediated telomerase activity, implying the benefit of EV loading in growing the clonal chondrocyte columns in the growth plate cartilage. Furthermore, studies will be necessary to elucidate the unknown molecular mechanism by which ERK1/2 controls self-renewal capacity, clonogenicity, and proliferation of CPCs through telomerase activity.

Indian hedgehog and parathyroid hormone-related peptide receptors are produced by chondrocytes in the hypertrophic and resting zones of the growth plate, respectively, and they target chondrocytes in the proliferating zone^38,39^. Indian hedgehog secreted from periosteal stem cells can also attack chondrocytes in the proliferation zone^40^. These findings support the cartilaginous permeability of EVs to target CPCs in the growth plate cartilage. OI model Oim/Oim mice with shortened body lengths display proliferation deficiency and cell cycle delay in chondrocytes of the proliferating zone^41^. Intravenous infusion of MSC-derived extracellular vesicles successfully promotes body length and long bone growth in Oim/Oim mice^42^. Recent studies have evaluated the epigenetic benefits of intravenously infused EVs on the functional restoration of target bone marrow MSCs in postmenopausal osteoporosis and autoimmune disease model mice^9,10^. Given the present efficacy of ex vivo and in vitro EV loading on the growth plate cartilage of mouse growing long bone and OI patient-derived CPC-like cells (Supplementary Fig. 13 and Fig. 8), EVs may have potential benefits for targeting growth plate dysfunction, including self-renewal and proliferation of CPCs. Further preclinical animal studies are necessary to address the delivery, dosing, and potential immunogenicity of EVs for the clinical option to pediatric patients with dwarfism or OI.

Taken together, in this study, we demonstrated that SHED-derived EVs stimulate self-renewal and proliferation of CPCs via the ERK1/2–TERT-telomerase activity pathway (Fig. 8D). Pharmacologically, paracrine efficacy of EVs derived from the other dental-tissue-specific stem cells, including permanent tooth dental pulp stem cells (DPSCs), periodontal stem cells (PDLSCs), and gingival MSCs (GMSCs), has been reported to accelerate osteoblast differentiation and anti-inflammatory action^43–45^, expecting the potential contribution of DPSC-, PDLSC-, and GMSC-derived EVs to CPC proliferation. Thus, the present findings imply future EV-based cell-free therapy for pediatric patients with skeletal disorders associated with dwarfism.

## Methods

### Ethics approval and consent to participate

We obtained human deciduous teeth as clinically discarded materials from healthy pediatric donors (n = 3, 2 male and one female, aged 5–7 years) and patients with OI (n = 2, aged 7–8 years) due to the eruption of permanent teeth at the Department of Pediatric Dentistry, Kyushu University Hospital. The pediatric patients with OI type III (a 7-year-old female and an 8-year-old male) were clinically diagnosed as OI type III with severe multiple fractures, scoliosis, short stature, and restricted mobility with hearing loss, cardiovascular, and respiratory complications. However, they did not examine their genetic background. The handling of human samples was approved by the Institutional Review Boards/Ethics Committees of Kyushu University Hospital and Medical Institutions for the study titled “Research on congenital diseases using cells derived from oral tissues”, with protocol no. 678–03, granted on March 15th, 2021. All the experimental methods in this manuscript were performed in accordance with the relevant guidelines and regulations. We also obtained written informed consent from the legal guardians of all participants in this study.

All animal experiments were approved by the Institutional Animal Care and Use Committee of Kyushu University for the study titled “Regulation of chondrocyte proliferative potential by extracellular vesicles derived from dental pulp stem cells”, with approval no. A23-167-0, granted on April 1st, 2023. All the experimental methods in this study were performed in accordance with the relevant guidelines and regulations and reported in accordance with ARRIVE guidelines 2.0. This study utilized human chondrosarcoma cell line OUMS-27 cells (JCRB Cell Bank, IFO50488) purchased from the Japanese Collection of Research Biosources Cell Bank (Osaka, Japan). The supplier provided the derivation of these cell lines with the appropriate ethical approval and the donor’s informed consent. The supplier provided the detailed ethical information.

### Antibodies

All antibodies used in this study are summarized in Supplementary Tables 1 and 2.

### Isolation and culture of human CPCs

We purchased cryopreserved primary human chondrocytes (HCs; passage 1) from fetal articular condyles from Cell Applications (Catalog number 402-05f.; San Diego, CA, USA) and used them to isolate human CPCs using the CFU-F method^46^. The cryopreserved HCs were thawed quickly, seeded in T-75 flasks (Corning, Corning, NY, USA) at 1 × 10^3^ cells/flask, cultured in an HC growth medium at 37 °C with 5% CO_2_ in a CO_2_ incubator (Forma, Thermo Fisher Scientific, Waltham, MA, USA). The HC growth medium consisted of HC basal medium (Cell Applications), HC Growth Supplement (Cell Applications), and penicillin–streptomycin premixed solution (P/S; 100 U/mL penicillin and 100 µg/mL streptomycin; Nacalai Tesque, Kyoto, Japan). Single attached cells were obtained by washing with PBS (pH 7.4) 16 h after seeding and cultured for 14 days until they formed adherent colonies. Colony-forming cells were passaged and expanded in the HC growth medium. The medium was exchanged twice a week. Population doubling was analyzed until the CFU-F cells reached passage 24. Finally, the passaged 3 cells were analyzed to characterize CPC signatures of CFU-F formation, immunophenotype, and multipotency into adipocytes, chondrocytes, and osteoblasts^46^. Passage 10 cells were used for further experiments.

### Genome editing of the CD29 gene

The *CD29* gene was edited in CPCs using a lentiviral CRISPR/Cas9 system. CPCs were seeded at 2.5 × 10^5^ cells per well in a 60 mm culture dish (Corning) and cultured in antibiotic-free minimum essential medium Eagle alpha modification (αMEM; Thermo Fisher Scientific) supplemented with 10% fetal bovine serum (FBS; Equitech-Bio, Kerrville, TX, USA), referred to as 10% FBS/αMEM. The cells were co-transfected with 1 × 10^7^ TU/f LentiArray^TM^Cas9 lentivirus (Thermo Fisher Scientific) and 1 × 10^8^ TU/f LentiArray™ gRNA Lentivirus for *CD29* (Thermo Fisher Scientific) in 10% FBS/αMEM supplemented with 5 μg/mL hexadimethrine bromide (Nacalai Tesque). Forty-eight hours after transfection, the cells were incubated with 3 μg/mL blasticidin (Nacalai Tesque) and 2 μg/mL puromycin (Nacalai Tesque) in 10% FBS/αMEM for five days and referred to as Cas9CD29-CPCs. Single Cas9CD29-CPCs were cultured using the limiting dilution method, expanded to analyze *CD29* expression using RT-qPCR, and obtained CD29^KO^CPCs and their control wild-type CPCs. CD29 levels in CD29^KO^CPCs and CD29^WT^CPCs were analyzed using immunoblotting and flow cytometry.

### Isolation and culture of SHED

SHED were isolated and cultured according to previously described protocols^46^. Dental pulp tissues were extracted from human exfoliated deciduous teeth and treated with an enzyme cocktail, consisting of 0.3% collagenase type I (Worthington Biochemicals, Lakewood, NJ, USA) and 0.4% dispase II (Sanko Junyaku, Tokyo, Japan) in PBS, for 60 min at 37 °C. The cell suspension was passed through a 70-μm cell strainer (Corning). The obtained single-cell population was incubated in T-75 culture flasks (Corning) with an MSC growth medium and maintained at 37 °C with 5% CO2 in a CO2 incubator (Forma, Thermo Fisher Scientific).

Single attached cells were obtained by washing with PBS (pH 7.4) 16 h after seeding and cultured for 14 to 16 days until they formed adherent colonies. Adherent colony-forming cells were passaged and expanded in the MSC growth medium. The growth medium consisted of 15% FBS (Equitech-Bio), 100 µM L-ascorbic acid 2-phosphate (FUJIFILM Wako Pure Chemicals, Osaka, Japan), 2 mM L-glutamine (Nacalai Tesque), and P/S (Nacalai Tesque) in αMEM (Thermo Fisher Scientific). The MSC growth medium was changed twice a week. Passage 3 SHED were analyzed to characterize MSC signatures of CFU-F formation, immunophenotype, and multipotency into adipocytes, chondrocytes, and osteoblasts, and were used for further experiments^47^.

*Establishment of CD29* ^+^ *SHED and CD29* ^–^ *SHED population using fluorescent cell sorting.*

CONT-SHED were seeded at 2.5 × 10^5^ cells/dish in 100 mm dishes and expanded in MSC medium. CONT-SHED were stained with 7AAD and RPE-conjugated anti-CD29 and isotype-matched antibodies and fractioned to CD29^+^SHED and CD29^–^SHED on a cell sorter FACSAria Fusion (BD Biosciences) using the software FACDiva (BD Biosciences). CD29 levels in CD29^+^SHED and CD29^–^SHED were analyzed using flow cytometry and immunoblotting.

### CFU-F assay

Isolated dental pulp cells and thawed chondrocytes were seeded at 1 × 10^3^ cells/dish in 100-mm culture dishes (Corning) and cultured in MSC growth and HC growth media for 16 days. The cells were treated with 2% paraformaldehyde (Millipore, Darmstadt, Germany) and 0.5% toluidine blue (Millipore) in PBS (pH 7.4). The colonies were acquired using a scanner GTX970 (EPSON, Tokyo, Japan). The number of colonies containing over 50 cells was measured using an inverted microscope Primo Vert (Carl Zeiss Microscopy, Jena, Germany).

### Immunophenotype assay

Cells (1 × 10^5^) were stained with R-phycoerythrin-conjugated specific antibodies to CD166, CD146, CD105, CD90, CD73, CD49D, CD49E, CD44, CD29, CD45, CD34, CD19, CD14, CD11b, CD324, CD235A, CD31, and HLA-DR (Supplementary Table 1) and analyzed using flow cytometry.

### Mesenchymal multipotent assay

Cells were seeded at 5 × 10^3^/dish and grown on 60-mm dishes (Corning) until they reached confluence. To analyze adipogenic potency, the cells were induced in an adipogenic induction medium consisting of MSC growth medium supplemented with 500 μM isobutyl-methylxanthine (Millipore), 60 μM indomethacin (Millipore), 0.5 μM hydrocortisone (Millipore), and 10 μM insulin (Millipore). To analyze chondrogenic potency, the cells were maintained in a chondrogenic induction medium consisting of Dulbecco’s modified Eagle’s medium with 1 g/L glucose (lowDMEM; Thermo Fisher Scientific) supplemented with 15% FBS (Equitech-Bio), 2 mM L-glutamine (Nacalai Tesque), 100 μM L-ascorbate-2-phosphate (FUJIFILM Wako Pure Chemicals), 2 mM sodium pyruvate (Nacalai Tesque), 1% Insulin-Transferring-Selene mixture (BD Biosciences, Franklin Lakes, NJ, USA), 100 nM dexamethasone (Millipore), 10 ng/mL transforming growth factor beta 1 (PeproTech, Rocky Hill, NJ, USA), and P/S (Nacalai Tesque). To analyze osteogenic potency, the cells were cultured in an osteogenic induction medium consisting of MSC growth medium supplemented with 1.8 mM potassium dihydrogen phosphate (Millipore) and 10 nM dexamethasone (Millipore) for 1 or 4 weeks. The adipogenic, chondrogenic, and osteogenic induction medium was changed twice a week. The cultures were analyzed using lineage-specific staining with Oil-Red-O, Alcian blue, and Alizarin Red S dyes. Lineage-specific gene expression was analyzed using RT-qPCR for adipocytes [*peroxisome proliferator-activated receptor gamma 2* and lipoprotein lipase] and chondrocytes (*SRY-box 9*, *aggrecan*, *collagen type 2 alpha chain 1*, and *collagen type 10 alpha chain 1*).

### Manufacturing of CM

SHED were seeded at 2.5 × 10^5^ cells/dish in 100 mm dishes and cultured until 70–80% confluence in an EV-depleted complete medium. The EV-depleted complete medium consisted of 15% EV-depleted FBS, 100 µM L-ascorbic acid 2-phosphate (FUJIFILM Wako Pure Chemicals), 2 mM L-glutamine (Nacalai Tesque), and P/S (Nacalai Tesque) in αMEM (Thermo Fisher Scientific). The EV-depleted FBS was prepared from FBS (Equitech-Bio) by centrifugation at 4 °C for 16 h at 10,000 × *g* using an ultra-high speed centrifugal machine, himac CP80a (Hitachi, Tokyo, Japan) equipped with a swing rotor P40ST (Hitachi) in 13PA tubes (Hitachi). SHED cultures were washed with PBS and subsequently incubated in FBS-free and antibiotic-free lowDMEM (Thermo Fisher Scientific) for 48 h. CM was manufactured at 4ºC from the culture supernatants by sequential centrifuging at 440 × *g* for 5 min in 50 mL tubes (Corning) on a centrifugal machine, Allegra X-30R (Beckman Coulter, Brea, CA, USA) equipped with a swinging rotor SX4400 (Beckman Coulter) and subsequently centrifuged at 10,000 × *g* for 30 min in 50 mL tubes (Corning) on an ultracentrifuge machine, himac CP80a (Hitachi) equipped with a swing rotor P40ST (Hitachi). Then, CM was ten-fold-enriched using a centrifugal filter, Amicon Ultra (Merck, Darmstadt, Germany), at 1500 ×*g* for 30 min at 4 °C on a centrifugal machine, Allegra X-30R (Beckman Coulter) equipped with a swinging rotor SX4400 (Beckman Coulter), according to the manufacturer’s instructions.

### Purification, RNase treatment, and characterization of EVs

SHED were cultured until 70–80% confluence in the EV-depleted complete medium and incubated in the FBS-free and antibiotic-free lowDMEM (Thermo Fisher Scientific) for 48 h to collect CM. EVs were purified from CM using the membrane affinity spin column method with an exoEasy Maxi kit (Qiagen, Valencia, CA, USA) on a centrifuge machine Allegra X-30R (Beckman Coulter) equipped with a swinging rotor SX4400 (Beckman Coulter), according to the manufacturer’s protocol^9^. CM was negatively fractioned through a membrane affinity spin column, and EV-depleted CM was collected. Some EVs were treated with 5 U/mL RNase (Thermo Fisher Scientific) or PBS (Mock) at 37 °C for 3 h and incubated with 40 U/mL RNase inhibitor (Thermo Fisher Scientific) at room temperature for 10 min according to previous reports^9,10^ and obtained RNase-treated EVs and untreated EVs. CD29^+^EVs and CD29^–^EVs were extracted from the CM of CD29^+^SHED and CD29^–^SHED cultures through membrane affinity spin columns.

The particle size of EVs was analyzed using nanoparticle tracking analysis with a nanoparticle tracking system NanoSight N3000 (Malvern Panalytical, Malvern, UK). To analyze the ultrastructure of EVs, EVs were mounted on a Formvar-membraned copper grid (#300; Nisshin EM, Tokyo, Japan), stained with an electron negative stain (Nisshin EM), and observed under a transmission electron microscope JEM 1400 + (JEOL, Tokyo, Japan) at 80 kV. Expression of extracellular vesicle markers was analyzed using immunoblotting and flow cytometry. To analyze internalization of EVs, EVs (4 mg/mL) were labeled with fluorescent dyes, including CFSE (Thermo Fisher Scientific) and PKH67 (Millipore). CPCs were loaded with the fluorescent dye-labeled EVs for 10 min and fixed with 4% paraformaldehyde (Millipore) in PBS (pH 7.4) for 15 min. They were stained with 4′,6-diamidino-2-phenylindole (DAPI; Dojindo, Kumamoto, Japan) and observed under an upright fluorescence microscope Axio Observer (Carl Zeiss Microscopy) equipped with an optical sectioning slider ApoTome 2 (Carl Zeiss Microscopy) and a microscope camera Axiocam 305 mono (Carl Zeiss Microscopy). Total RNA was extracted from EVs using the miRNeasy Mini Kit (Qiagen, Valencia, CA, USA) and quantified using small RNA chips (Agilent, Santa Clara, CA, USA) on a bioanalyzer Agilent 2100 (Agilent). Total protein was extracted from EVs and separated on a TGX FastCast acrylamide gel (Bio-Rad Laboratories, Hercules, CA, USA). The gels were stained with CBB using Coomassie Brilliant Blue R-250 Staining Solution (Bio-Rad Laboratories). The images were acquired using a gel imager, GelDoc EZ (Bio-Rad Laboratories). Total protein concentration was measured using a Bio-Rad protein assay (Bio-Rad Laboratories) with a spectrometer Multiskan GO microplate (Thermo Fisher Scientific).

### CM and EV treatment

The HC growth medium was mixed with ten-fold CM to one-tenth of the medium or EVs at 4, 20, and 40 μg/mL. Passage 10 CPCs were incubated with the CM- or EV-mixed HC growth medium and harvested on day 4. As the controls, PBS (Mock) was used instead of CM or EVs.

### ERK1/2 inhibitor treatment

Passage 10 CPCs were pretreated with an ERK1/2 inhibitor, PD98059 (10 μM, Merck), diluted in 10% DMSO (Wako Pure Chemical) or 10% DMSO (Wako Pure Chemical) as Mock, in the HC growth medium for one day.

### Anti-CD29 antibody blocking

Anti-CD29 IgG_1_ (10 μg/mL; Abcam, Cambridge, UK) and their control IgG1 (10 μg/mL; Abcam) were added with in the HC growth medium when CPC were treated with EVs.

### Population doubling assay

The cells were sequentially passaged every seven days. The culture medium was changed every three days. PDL and PDT at each passage were calculated with the following equations: PDL = (log [harvested cell number at each passage]—log [seeding cell number at each passage]) × 3.33 and PDT = culturing time (hours) × log2/([harvested cell number at each passage]—[seeding cell number at each passage]), respectively.

### Cell viability and cell proliferation assays

Passage 10 CPCs (2.0 × 10^3^ per well) were seeded in 96-well multi-plates and treated with CM or EVs three days after cell seeding. Twenty-four hours after CM or EV treatment, the cell viability and proliferation ability of CPCs were analyzed using WST8 and BrdU incorporation assays using the Cell Counting Kit 8 (Dojindo) and Cell Proliferation ELISA, BrdU (Merck), respectively, according to the manufacturer’s instructions. The Cell Counting Kit 8 solution (10 μL; Dojindo) was added to the HC growth medium 4 h before the WST assay. The labeling reagent for BrdU (Merck) was added to the HC growth medium 12 h before analysis. The results were measured using a microplate spectrometer, Multiskan GO (Thermo Fisher Scientific).

### Telomerase activity assay

Passage 10 CPCs (1.0 × 10^5^ cells per dish) were seeded on 60 mm dishes and treated with CM or EVs three days after cell seeding. Twenty-four hours after CM or EV treatment, telomerase activity (1.0 × 10^6^ cells per test) was analyzed using TRAP-PCR with Telomerase Activity Quantification qPCR Assay kit (Science Cell Research Laboratories, Carlsbad, CA, USA) according to the manufacturer’s instructions. Telomeric repeats (TTAGGG) were measured using a real-time PCR machine, LightCycler 96 system (Roche, Basel, Switzerland). Human chondrosarcoma cell-lined cells, OUMS-27 cells (P144, JCRB Cell Bank, Osaka, Japan), were cultured with 10% FBS (Equitech-Bio) and P/S (Nacalai Tesque) in low-glucose DMEM (Thermo Fisher Scientific) and used as a positive control. Some extracts from the respective samples were heated at 85 °C for 10 min and used as negative controls. The relative telomerase activity of different samples was calculated according to the manufacturer’s instructions.

### Cell cycle analysis using propidium iodide staining

Passage 10 CPCs were seeded at 2.0 × 10^5^ per dish on 100 mm dishes and then maintained under serum-depleted conditions for 15 h. The CPCs were then incubated for 3, 6, 12, 24, or 32 h in fresh HC growth medium with or without EVs. The cell cycle machinery was assayed using flow cytometry with a propidium iodide staining solution (Abcam) according to the manufacturer’s instructions and measured using a cell analyzer FACSVerse (BD Biosciences). The results were analyzed using FACSuite software (BD Biosciences).

### Cell cycle analysis using BrdU-7AAD staining

Passage 10 CPCs were seeded at 2.0 × 10^5^ per dish on 100 mm dishes and then maintained under serum-depleted conditions for 15 h. The CPCs were then incubated for 24 h in fresh HC growth medium with or without EVs. The cell cycle machinery was assayed using flow cytometry with a BrdU Flow kit (BD Biosciences) according to the manufacturer’s instructions and measured using a cell analyzer, FACSVerse (BD Biosciences). The results were analyzed using FACSuite software (BD Biosciences).

### Kinetics of ERK1/2

The kinetics of ERK1/2 and its phosphorylated form, pERK1/2, in CPCs were analyzed at 0, 5, 10, 15, 30, and 60 min after EV loading using immunoblotting.

### Ex vivo* mouse long bone analysis*

Pregnant C57BL/6 J female mice were purchased from Jackson Laboratory Japan (Tokyo, Japan). According to the American Veterinary Medical Association Guidelines for the Euthanasia of Animals (2020 Edition), since mouse pups are neurologically immature due to the underdevelopment of the brain cortex and afferent pain pathways until after postnatal day 5 to 7, decapitation using scissors and blades is acceptable with a euthanasia method for altricial neonates. In this study, neonatal mice (1-day-old) were euthanized by decapitation using sharp scissors without any use of an anesthetic agent. The tibiae were aseptically harvested and cultured in 24-well multiplates with lowDMEM (Thermo Fisher Scientific) supplemented with 10% FBS (Equitech-Bio) and a penicillin–streptomycin mixed solution (Nacalai Tesque). EVs (100 μg/mL) or PBS (Mock) were added to the ex vivo bone cultures on day 2. The bone samples were also treated with or without BrdU reagent (1 mg/mL; Merck) 24 h before harvest, harvested on day 5, and used for histological and immunohistochemical assays.

### Flow cytometry

Cultured cells (1.0 × 10^5^/100 µL) were incubated with R-phycoerythrin-conjugated specific and isotype-matched antibodies (Supplementary Table 1) in Hank’s balanced salt solution (Nacalai Tesque) supplemented with 2% heat-inactivated FBS (Equitech-Bio) and analyzed on a cell analyzer FACSVerse (BD Biosciences). The number (percentage) of positive cells was determined using FACSuite software (BD Biosciences) and compared with the corresponding control samples stained with their corresponding isotype-matched antibody with a false-positive rate of less than 1%.

### RT-qPCR

Cultured cells were lysed with a TRIzol reagent (Thermo Fisher Scientific). The extracts were digested with DNase I (Promega, Madison, WI, USA) and cleaned using an RNeasy Mini Kit (Qiagen, Valencia, CA, USA). cDNA was prepared through reverse transcription using a ReverTra Ace qPCR kit (TOYOBO, Osaka, Japan), according to the manufacturer’s instructions, and mixed with Man Gene Expression Master Mix (Thermo Fisher Scientific) and target TaqMan probes (Thermo Fisher Scientific). Gene expression was analyzed using a real-time PCR system, LightCycler 96 (Roche, Basel, Switzerland). Human 18S ribosomal RNA was used for normalization. All TaqMan probes used in this RT-qPCR are listed in Supplementary Table 3.

### Immunoblotting

Cultured cells and EVs were lysed with an M-PER mammalian protein extraction reagent (Thermo Fisher Scientific) containing a proteinase inhibitor cocktail (Nacalai Tesque) and the phosphatase inhibitor PhoSTOP (Roche). Total protein (10 mg/lane) was separated on TGX FastCast acrylamide gels (Bio-Rad Laboratories, Hercules, CA, USA) and transferred to a polyvinylidene difluoride (PVDF) Immun-Blot PVDF membrane (Bio-Rad Laboratories) using a Trans-Blot Turbo transfer system (Bio-Rad Laboratories). The membranes were blocked with 5% skim milk in Tris-buffered saline (150 mM NaCl and 20 mM Tris–HCl, pH 7.2) containing 0.1% Tween 20 for 1 h at room temperature and then incubated with primary antibodies overnight at 4 °C. The cells were then incubated with an HRP-conjugated secondary antibody (1:1000; Santa Cruz Biotechnology, Santa Cruz, CA, USA) for 1 h at room temperature. The membranes were treated with WB Stripping Solution Strong (Nacalai Tesque) and stained with an anti-actin beta antibody (Merck) as an internal control, followed by incubation with HRP-conjugated secondary antibody (1:1000; Santa Cruz Biotechnology). The membrane images were acquired using SuperSignal West Pico (Thermo Fisher Scientific) on a membrane imager, ImageQuant LAS 4010 (GE Healthcare Life Sciences, Pittsburgh, PA, USA) or ImageQuant 800 (Cytiba, Marlborough, MA, USA). All antibodies used in immunoblotting are listed in Supplementary Table 2. Immunoblot band density was measured using ImageJ software (NIH, Bethesda, MD, USA).

### Histological and immunohistochemical assay

Ex vivo cultured bone samples were fixed with 4% paraformaldehyde (Merck) in PBS at 4ºC, overnight. Some samples were embedded in OCT compound (Sakura, Tokyo, Japan) and quickly frozen using an ultralow freezing system Histo-Tek PINO (Sakura). Cryosections of the frozen samples were cut into 5 mm-thick sections using a cryostat CM1950 (Leica Biosystems, Wetzlar, Germany) and treated with a BrdU Immunohistochemistry Kit (Abcam), according to the manufacturer’s instructions. The sections were visualized with 0.05% diaminobenzidine (DAB; Dojindo), 0.025% cobalt chloride, 002% nickel ammonium, and 0.01% H_2_O_2_ in 0.05 mol/L Tris–HCl buffer (pH 7.4) and then counterstained with eosin. The other samples were cut into 300-μm-thick slices using a microslicer DSK 1000 (Dosaka EM, Kyoto, Japan). The samples were immersed in 2.3 M sucrose containing PBS (pH 7.4) at 4 °C and frozen in liquid nitrogen. Semi-thin (1 μm) sections were cut at − 70 °C on an ultramicrotome Ultracut E (Leica Microsystems) equipped with a cryo-attachment FC4E (Leica Microsystems). Cryosections were treated with 0.3% H_2_O_2_ in PBS (pH 7.2) for 60 min and incubated with 10% normal goat serum (Vector Laboratories, Burlingame, CA, USA) in PBS (pH 7.2) for 15 min. Semi-thin sections were incubated with an anti-pERK1/2 antibody (1:200) (Supplementary Table 2) for 90 min and then treated with the VECTASTAIN ABC Kit (Vector Laboratories), according to the manufacturer’s instructions. The sections were reacted with 0.02% DAB (Dojindo) and 0.006% H_2_O_2_ in 0.05 mol/L Tris–HCl buffer (pH 7.6) for 5 min and counterstained with methyl green. All sections were observed under an upright light microscope Axiolab (Carl Zeiss Microscopy) equipped with a microscope camera Axiocam 305 color (Carl Zeiss Microscopy).

### Statistical analysis

All analyses were performed by using at least, three samples per test and three repetition per analysis to obtain the results from one donor. Results from individual tests were averaged and calculated the means from, at least, three repetitions. Data are presented as the mean ± standard deviation (SD) of the averages for each group. Normality of the data was confirmed using Shapiro–Wilk test prior to performing statistical analyses. Comparisons between two groups were determined using an independent two-tailed Student’s *t*-test. Multiple group comparisons were determined using one-way analysis of variance (one-way ANOVA), followed by Tukey’s post hoc test. All statistical analyses were performed using PRISM 9 (GraphPad Software, La Jolla, CA, USA).

## Supplementary Information

Below is the link to the electronic supplementary material.

Supplementary Material 1

## Data Availability

All data generated or analyzed during this study are included in this published article and its Supplementary Information files.
