# Supplementary material for: Deciduous pulp stem cell-derived extracellular vesicles stimulate the proliferation of cartilage progenitor cells via extracellular signal-regulated protein kinase 1/2 activation
Source: Sci Rep. 2026 Mar 9;16:12654. doi: 10.1038/s41598-026-37380-7 (PMC13087214; doi:10.1038/s41598-026-37380-7)
Supplement: Supplementary file 1 — Supplementary Material 1 [file 41598_2026_37380_MOESM1_ESM.pdf]

Supplementary Information

Deciduous pulp stem cell-derived extracellular vesicles stimulate the proliferation of cartilage progenitor cells via extracellular signal-regulated protein kinase 1/2 activation.

Sara Murata, Soichiro Sonoda, Yukari Kyumoto-Nakamura, M. Majd Sharifa, Liting Yu, Reona Aijima, Erika Yamauchi-Tomoda, Fouad MHD. Zakaria, Hiroki Kato, Norihisa Uehara, Haruyoshi Yamaza, Takayoshi Yamaza

Supplementary Table 1. List of antibodies used for flow cytometry

Antibodies (Clone), conjugation	Product number	Dilution	Source
**Primary antibodies**			
anti-CD9 (6F3) mouse mAb, R-PE conjugated	sc-13118PE	5 μL/1 × 10^6 cell	SCB
anti-CD11b (ICRF44) mouse mAb, R-PE conjugated	301305	5 μL/1 × 10^6 cell	BioLegend
anti-CD14 (HCD14) mouse mAb, R-PE conjugated	325606	5 μL/1 × 10^6 cell	BioLegend
anti-CD19 (HIB19) mouse mAb, R-PE conjugated	982402	5 μL/1 × 10^6 cell	BioLegend
anti-CD29 (TS2/16) mouse mAb, Alexa Fluor 488 conjugated	303016	5 μL/1 × 10^6 cell	BioLegend
anti-CD29 (TS2/16) mouse mAb, R-PE conjugated	303004	5 μL/1 × 10^6 cell	BioLegend
anti-CD31 (JC70) mouse mAb, R-PE conjugated	623555	5 μL/1 × 10^6 cell	BioLegend
anti-CD34 (581) mouse mAb, R-PE conjugated	343516	5 μL/1 × 10^6 cell	BioLegend
anti-CD44 (BJ18) mouse mAb, R-PE conjugated	338808	5 μL/1 × 10^6 cell	BioLegend
anti-CD45 (HI30) mouse mAb, R-PE conjugated	304008	5 μL/1 × 10^6 cell	BioLegend
anti-CD49D (9F10) mouse mAb, FITC conjugated	304315	5 μL/1 × 10^6 cell	BioLegend
anti-CD49E (NKI-SAM-1) mouse mAb, APC conjugated	359310	5 μL/1 × 10^6 cell	BioLegend
anti-CD63 (H5C6) mouse mAb, unconjugated	561925	5 μL/1 × 10^6 cell	BD
anti-CD73 (AD2) mouse mAb, R-PE conjugated	344004	5 μL/1 × 10^6 cell	BioLegend
anti-CD81 (7F5) mouse mAb, unconjugated	sc-13118PE	5 μL/1 × 10^6 cell	SCB
anti-CD90 (5E10) mouse mAb, R-PE conjugated	328109	5 μL/1 × 10^6 cell	BioLegend
anti-CD105 (43A3) mouse mAb, R-PE conjugated	323206	5 μL/1 × 10^6 cell	BioLegend
anti-CD146 (P1H12) mouse mAb, R-PE conjugated	361006	5 μL/1 × 10^6 cell	BioLegend
anti-CD166 (3A6) mouse mAb, R-PE conjugated	343903	5 μL/1 × 10^6 cell	BioLegend
anti-CD235A (HI264) mouse mAb, R-PE conjugated	349106	5 μL/1 × 10^6 cell	BioLegend
anti-CD324 (67A4)) mouse mAb, FITC conjugated	324103	5 μL/1 × 10^6 cell	BioLegend
anti-HLA-DR(L243) mouse mAb, R-PE conjugated	307606	5 μL/1 × 10^6 cell	BioLegend
**Secondary antibodies**			
anti-mouse IgG1κ (RMG1-1) rat mAb, Alexa Fluor 488conjugated	406628	5 μL/1 × 10^6 cell	BioLegend
anti-mouse IgG1κ rat (RMG1-1) mAb, R-PE conjugated	406608	5 μL/1 × 10^6 cell	BioLegend
anti-rabbit IgG goat polyAb, Alexa Fluor 488 conjugated	4412	1:100	CST

APC, allophycocyanin; FITC, fluorescein isothiocyanate; mAb, monoclonal antibody; polyAb, polyclonal antibody; R-PE, R-phycoerythrin.

BD, BD Bioscience; CST; Cell Signaling Technology.

Supplementary Table 2. List of antibodies used for immunoblotting, immunohistochemistry, and blocking

Antibodies (Clone), conjugation	Product number	Dilution	Source
**Primary antibodies**			
anti-ACTB (AC-74) mouse mAb, unconjugated	A2228	1:5000	Merk
anti-ALB rabbit polyAb, unconjugated	4929	1:5000	CST
anti-ALIX (3A9) mouse mAb, unconjugated	2171	1:1000	CST
anti-BrdU (Bu20a) mouse mAb, unconjugated	5292	1:100	CST
anti-calnexin (C5C9) rabbit mAb, unconjugated	2679	1:1000	CST
anti-CD29 (JB1A) mouse mAb, unconjugated	MAB1965	1:1000	Millipore
anti-CD29 (P5D2) mouse mAb, unconjugated, BSA and Azide free	ab230293	10 μg/mL	Abcam
anti-ERK1/2 (3A7) mouse mAb, unconjugated	9107	1:1000	CST
anti-ERK1/2 (E10), phospho (Thr202/Try204) mouse mAb, unconjugated	9107	1:1000	CST
mouse IgG_1 (R312-MouseIgG1) Isotype control, unconjugated, BSA and Azide free	ab230293	10 μg/mL	Abcam
**Secondary antibodies**			
anti-mouse IgG goat polyAb, HRP conjugated	sc-2005	1:100	SCB
anti-rabbit IgG mouse mAb, HRP conjugated	sc-2357	1:100	SCB

Continued

ACTB, actin beta; ALB, albumin; ALIX, apoptosis-linked gene 2-interacting protein X; BrdU, 5-bromo-2'-deoxyuridine; ERK1/2, extracellular signal regulated kinase 1 and 2; HRP, horseradish peroxidase; mAb, monoclonal antibody; polyAb, polyclonal antibody;

CST; Cell Signaling Technology; SBI, System Bioscience Innovation; SCB, Santa Cruz Biotechnology

Supplementary Table 3. List of TaqMan probes used for qRT-PCR

Genes	Gene assay ID Numbers	Source
ACAN	Hs00153936_m1	Thermo Fisher Scientific
BGLAP	Hs01587814_g1	Thermo Fisher Scientific
COL2A1	Hs00264051_m1	Thermo Fisher Scientific
COL10A1	Hs00166657_m1	Thermo Fisher Scientific
LPL	Hs00173425_m1	Thermo Fisher Scientific
PPARG	Hs0115513_m1	Thermo Fisher Scientific
RUNX2	Hs00231692_m1	Thermo Fisher Scientific
SOX9	Hs01001343_g1	Thermo Fisher Scientific
TERT	Hs00972650_m1	Thermo Fisher Scientific
Ribosomal RNA, 18S	Hs99999901_s1	Thermo Fisher Scientific

ACAN, aggrecan; BGLAP, bone gamma-carboxyglutamate protein; COL2A1, collagen type II, alpha 1; COL10A1, collagen type X, alpha 1; LPL, lipoprotein lipase; PPARG, peroxisome proliferator-activated receptor gamma; RUNX2, RUNX family transcription factor 2; SOX9, SRY-box transcription factor 9; TERT, telomerase reverse transcriptase.

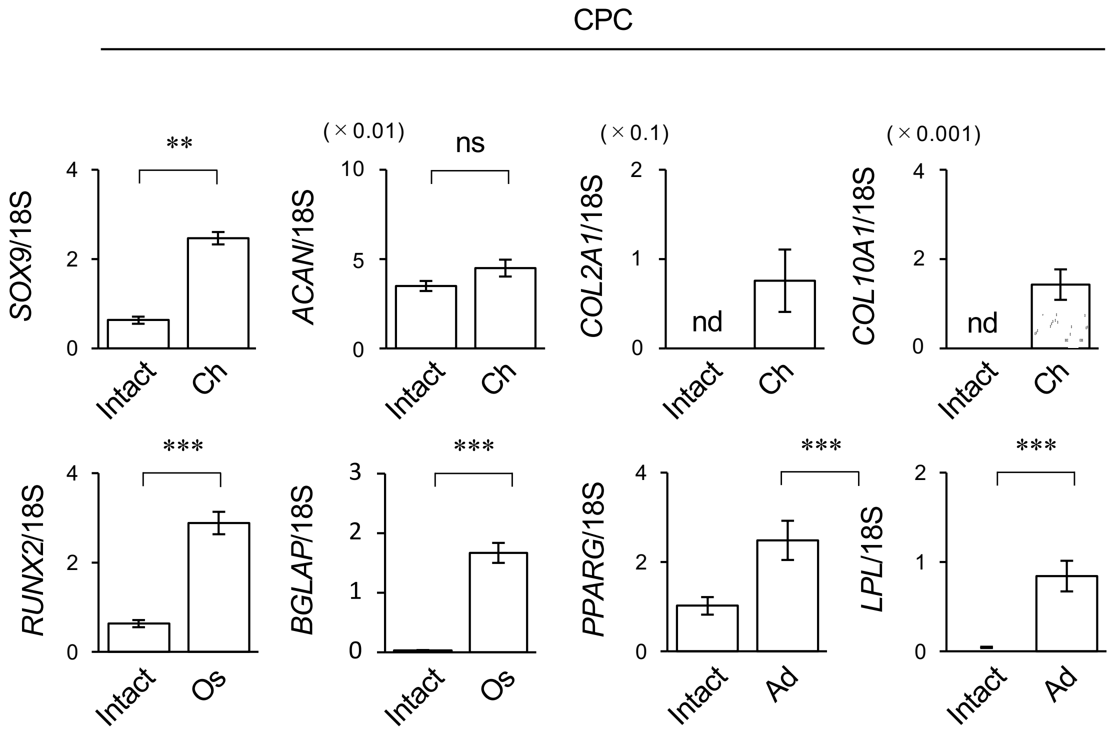

Supplementary Figure 1. Multipotency of CPCs. Graphs showing the results of gene expression of *SRY-box 9* (*SOX9*), *aggrecan* (*ACAN*), *collagen type II alpha 1 chain* (*COL2A1*), and *collagen type X alpha 1 chain* (*COL10A1*) specific for chondrocytes (Ch), *Runt-related transcription factor 2* (*RUNX2*) and *bone gamma-carboxyglutamate protein* (*BGLAP*) specific for osteoblasts (Os), and *peroxisome proliferator-activated receptor gamma* (*PPARG*) and *lipoprotein lipase* (*LPL*) specific for adipocytes (Ad) in CPCs using RT-qPCR. The results are shown as a ratio to 18S rRNA expression (/18S). Data are presented as mean ± SD. n = 3/group. Significance was determined using a two-paired *t*-test; $**P < 0.01$, $***P < 0.005$. nd, not detected, ns, not significant. Ad, adipogenic condition; Ch, chondrogenic condition; Intact, uninduced condition; Os, osteogenic condition.

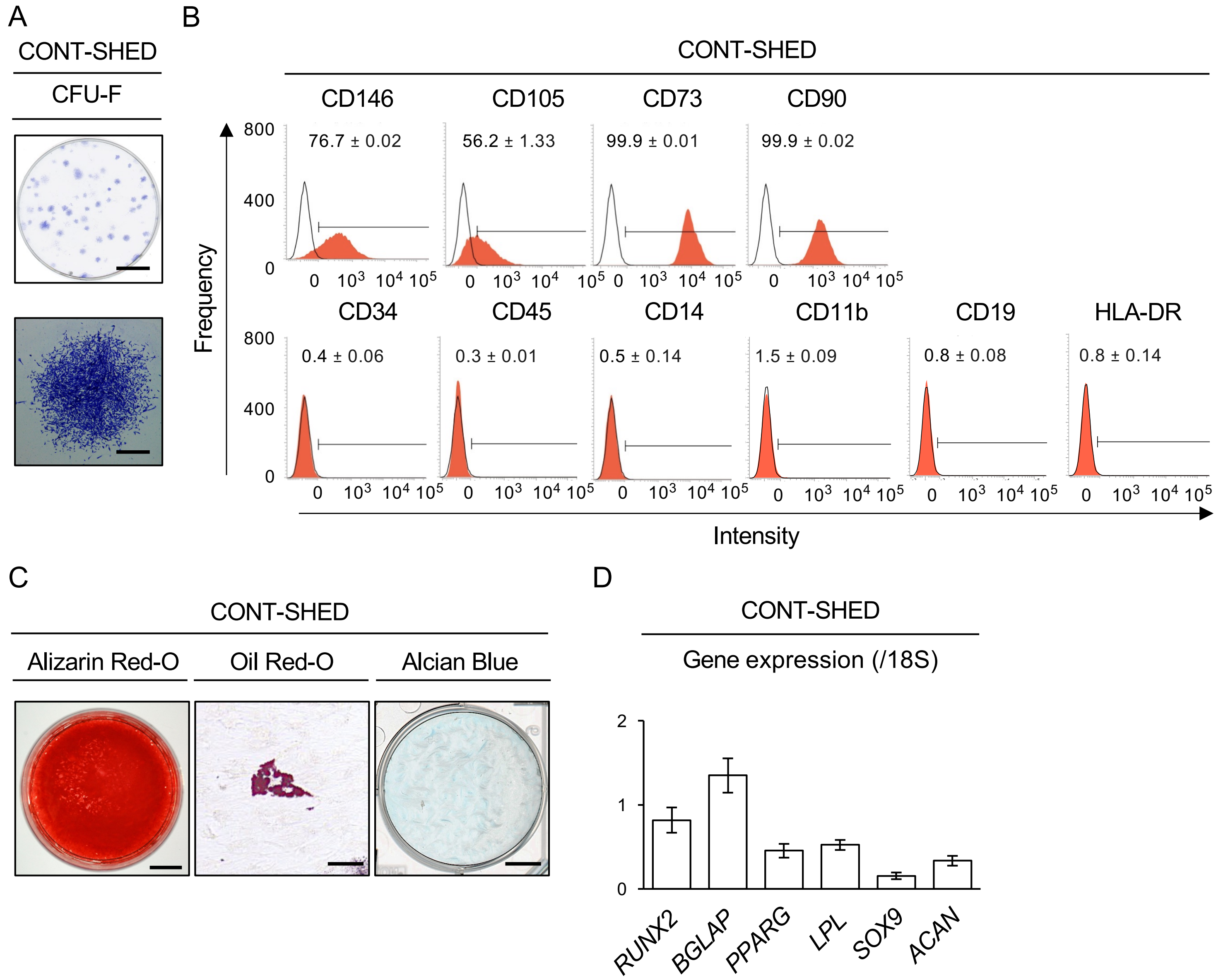

Supplementary Figure 2. Characterization of CONT-SHED. (A) Representative images of CFU-F colonies on a dish (upper image) and an adherent colony (lower image) using toluidine blue staining. Scale bar = 20 mm (upper), 500 µm (lower). (B) Representative histograms of CD146, CD105, CD73, CD90, CD34, CD45, CD14, CD11b, CD19, and HLA-DR expression using flow cytometry. Areas filled with red color, histograms stained with target antibodies; solid lines, histograms stained with isotype-matched control antibodies. The numbers indicate the positive ratio (%) of the cell surface. (C, D) Representative images of calcified nodule deposition, lipid accumulation, and cartilaginous matrix formation using Alizarin red, Oil red, and Alucian blue staining. Scale bar =20 mm (Alizarin red and Alucian blue staining), 100 µm (Oil red staining) (C). A graph showing the results of *RUNX2*, *BGLAP*, *PPARG2*, *LPL*, *SOX9*, and *ACAN* expression in CONT-SHED using RT-qPCR. The results are shown as a ratio to 18S rRNA expression (/18S) (D). (B, D) Data are presented as mean ± SD. n = 3/group.

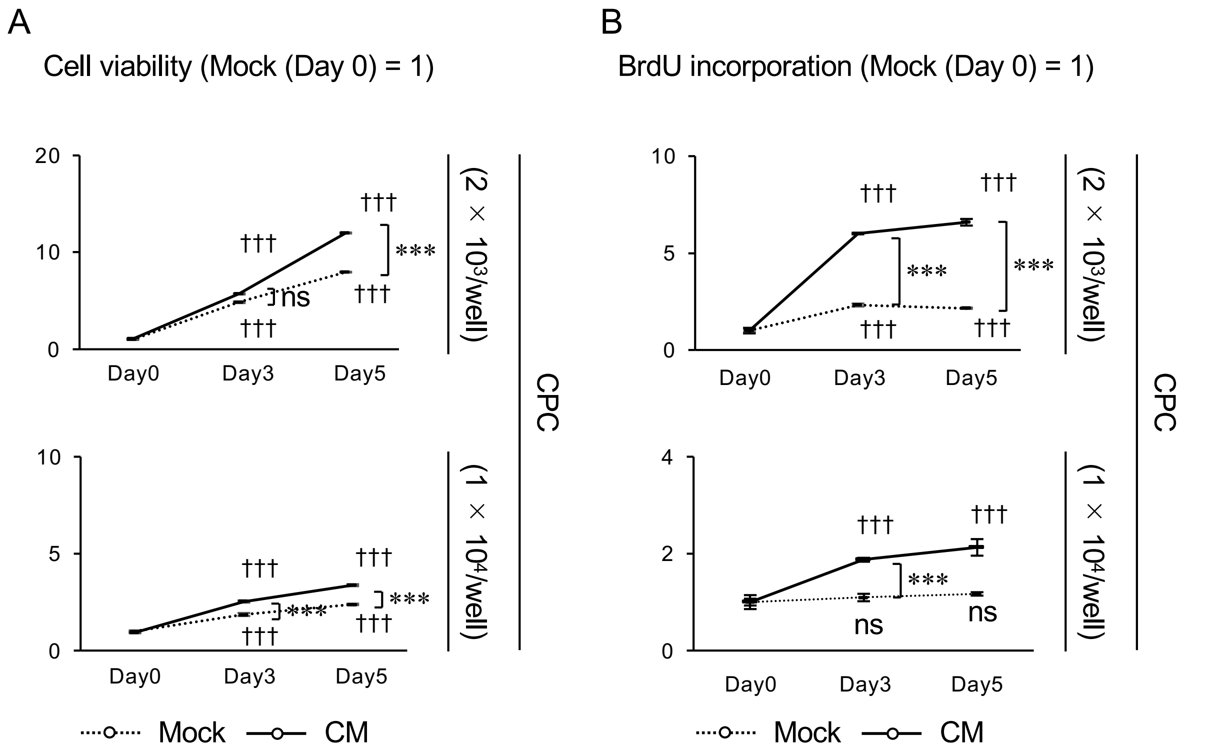

Supplementary Figure 3. Effect of CM on cell viability and proliferation in CPCs. (A, B) Graphs showing the results of cell viability (A) and proliferation (B) of CPCs (2×10^3 and 1×10^4 cells per well) on Day 0, 3, and 5 after CM loading using WST-8 and BrdU incorporation assays. The results are shown as a ratio to the result of PBS (Mock) loaded CPCs (Mock = 1). Data are presented as mean ± SD. n = 3/group. Significance was determined using a one-way ANOVA with Tukey's post hoc test; $***P < 0.005$, $\dagger\dagger\dagger\ P < 0.005$ versus the corresponding sample group on Day 0. ns, not significant.

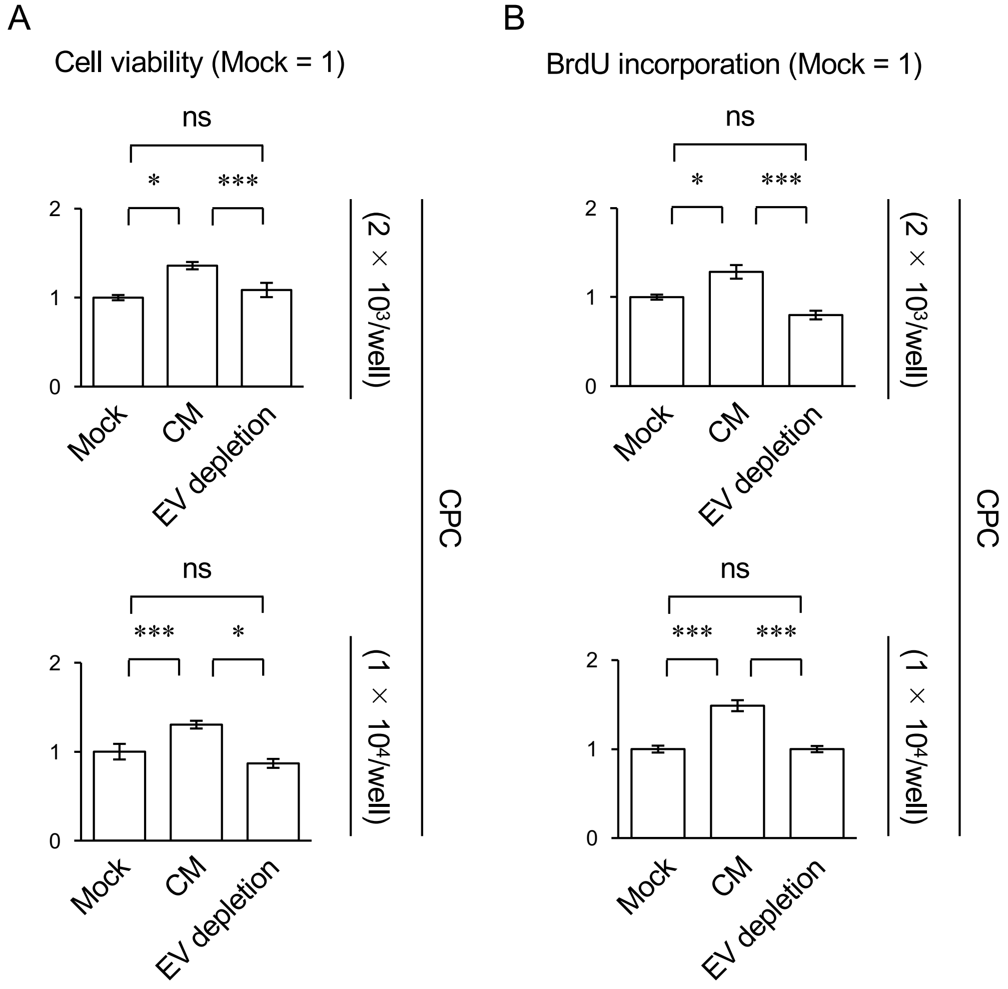

Supplementary Figure 4. Effect of EV-depletion on cell viability and proliferation of CM loading CPCs. (A, B) Graphs showing the results of cell viability (A) and proliferation (B) of CPCs (2×10^3 and 1×10^4 cells per well) on Day 3 after EV-depleted CM (EV depletion) loading using WST-8 and BrdU incorporation assays. The results are shown as a ratio to the result of PBS (Mock) loaded CPCs (Mock = 1). Data are presented as mean ± SD. n = 3/group. Significance was determined using a ones-way ANOVA with Tukey's post hoc test; $*P < 0.05$, $***P < 0.005$. ns, not significant.

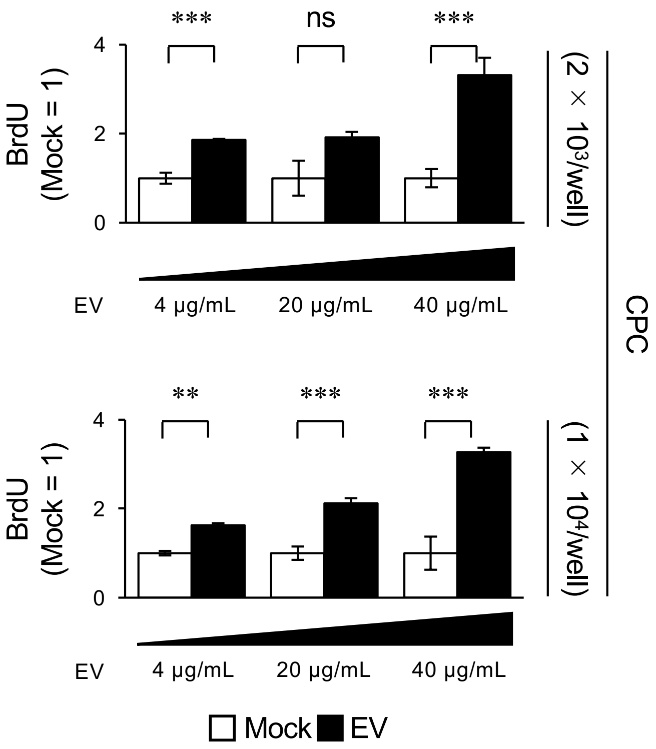

Supplementary Figure 5. Effect of SHED-derived extracellular vesicles on cell proliferation in CPCs. Graphs showing the results of cell proliferation of CPCs (2×10^3 and 1×10^4 cells per well) on Day 3 after EV and PBS (Mock) loading at 4, 20, and 40 μg/mL using BrdU incorporation assays. The results are shown as a ratio to the result of PBS (Mock) loaded CPCs (Mock = 1). Data are presented as mean ± SD. n = 3/group. Significance was determined using a two-tailed Student's *t* test; $^{**}P < 0.01$ and $^{***}P < 0.005$. ns, not significant.

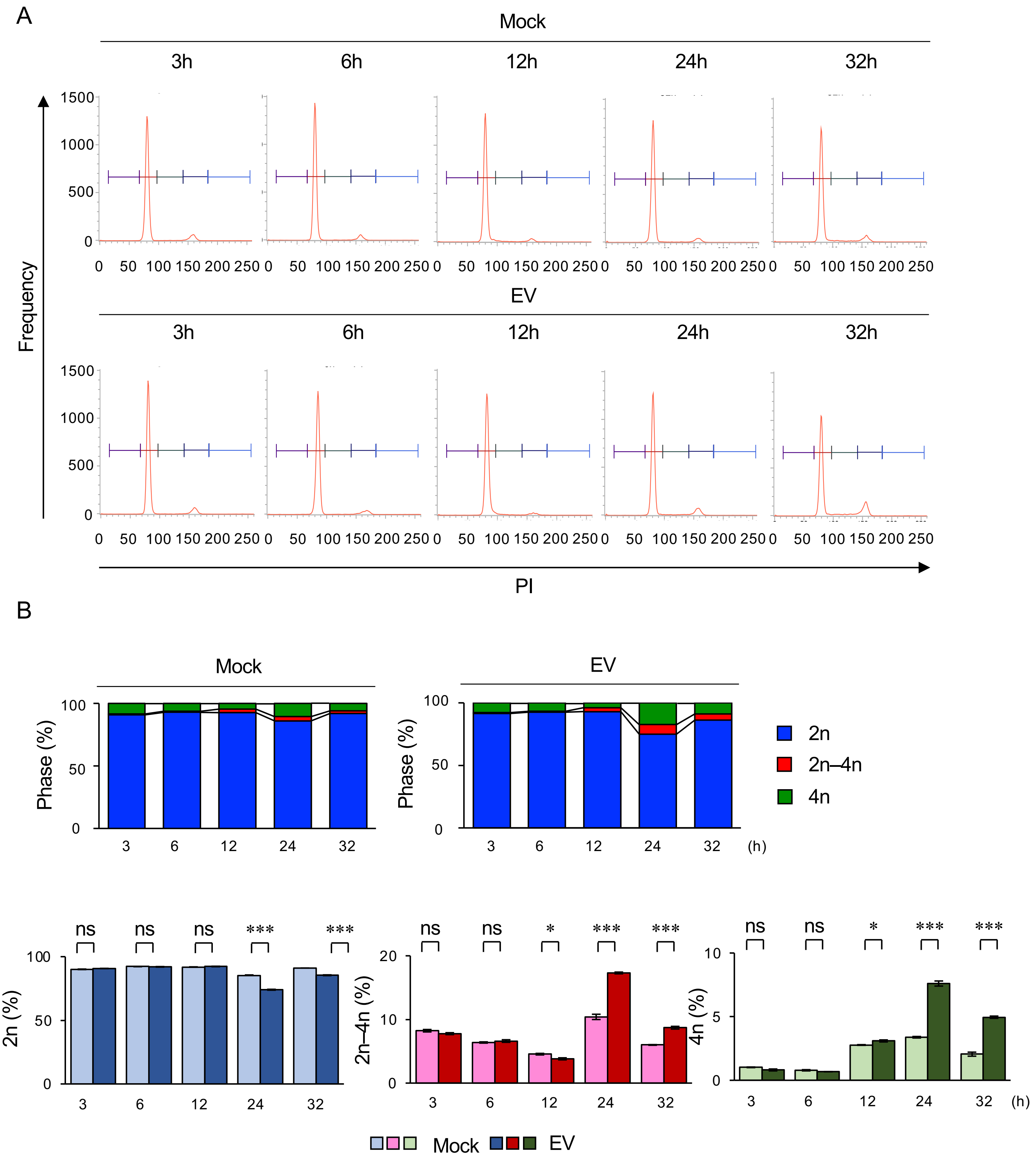

Supplementary Figure 6. EVs affect cell cycle in CPCs using propidium iodide staining. CPCs are treated with EVs (4 µg/mL) and PBS (Mock). The cells are maintained for 12 hours under a serum-starved condition before harvesting. (A) Representative histograms of DNA contents in CPCs 3, 6, 12, 24, and 32 hours (h) after EV treatment using flow cytometry using propidium iodide (PI) staining. (B) Graphs showing rate (%) of DNA contents (2n [G0/G1 phase], 2n–4n [S phase], and 4n [G2/M phase]) in total DNA of CPCs 3, 6, 12, 24, and 32 hours (h) after EV loading using flow cytometry with propidium iodide staining. Data are presented as mean ± SD. n = 3/group. Significance was determined using a two-tailed Student's t test; $^*P < 0.05$, $^{***}P < 0.005$. ns, not significant.

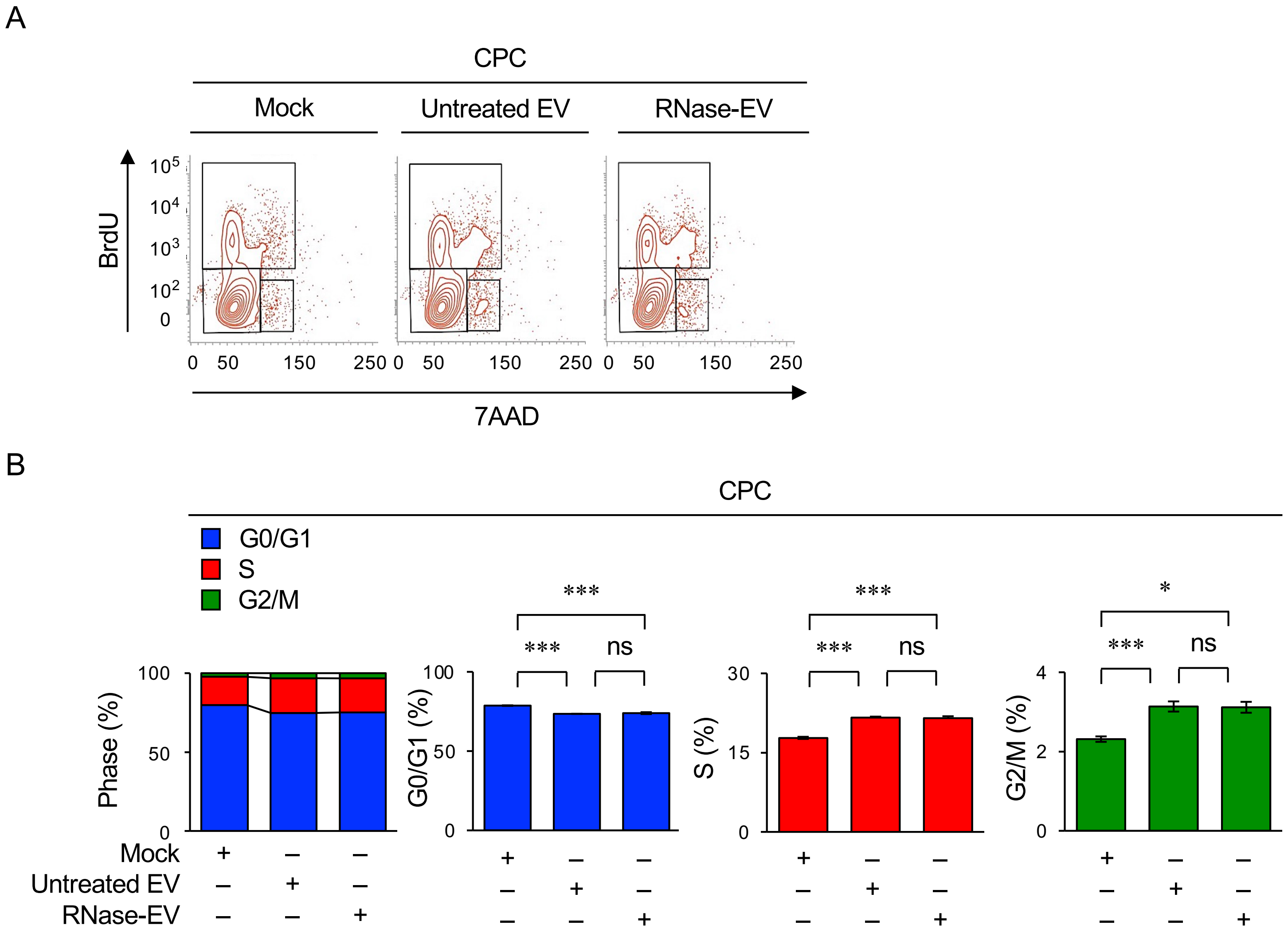

Supplementary Figure 7. Effects of EVs on the cell cycle of human CPCs. CPCs were treated with PBS (Mock), PBS-treated EVs (untreated EVs) (4 μg/mL), and RNase-treated EVs (4 μg/mL). (A) Representative contour plots of the cell cycle in CPCs assessed using flow cytometry with BrdU-7AAD staining. (B) Graphs showing the percentage (%) of the G0/G1, S, and G2/M phases in CPCs using flow cytometry. n = 3/group. Data are presented as mean ± SD. Significance was determined using a one-way ANOVA with Tukey's post-hoc test. * $P < 0.05$, *** $P < 0.005$. ns, not significant.

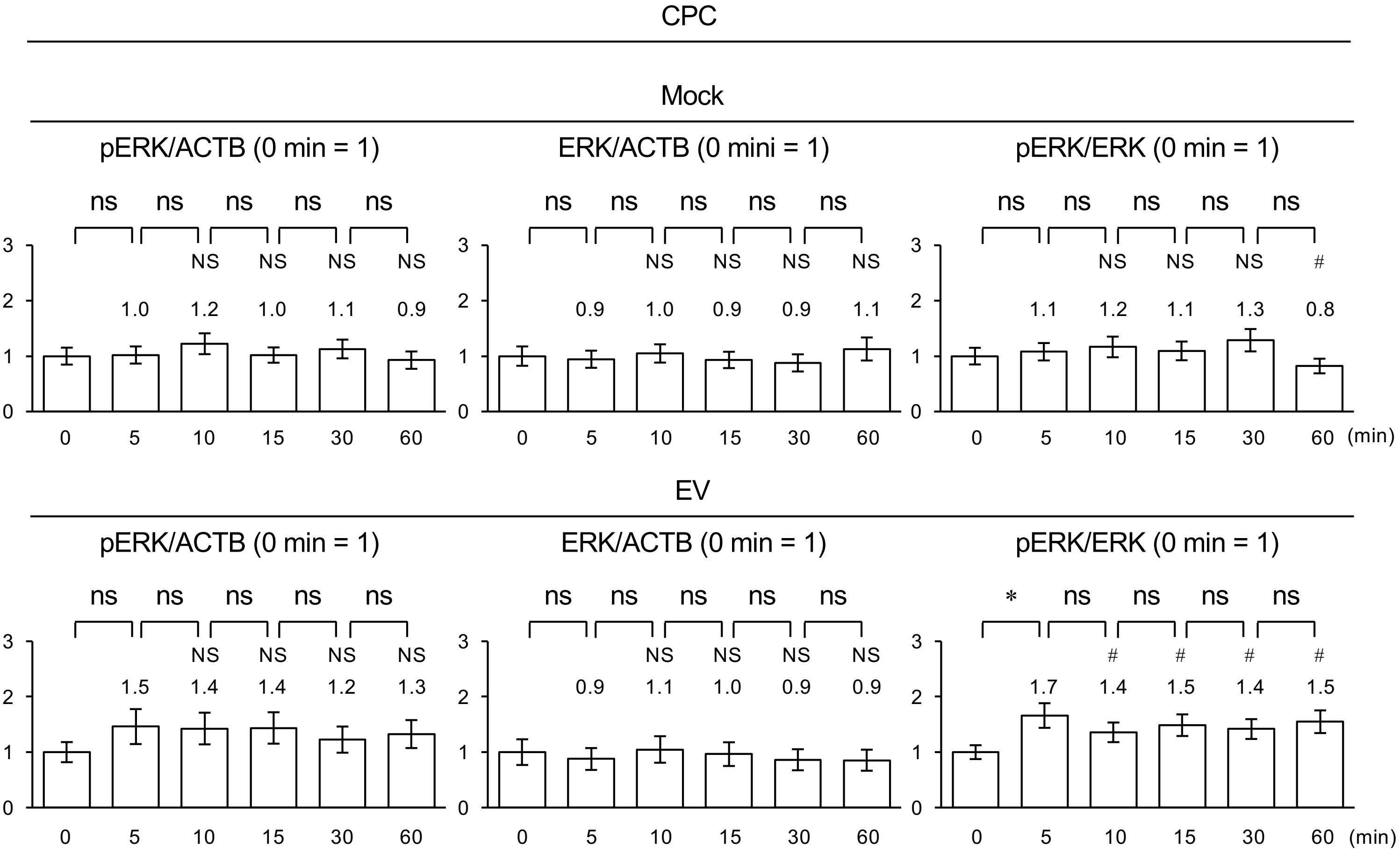

Supplementary Figure 8. Effects of EVs on the expression of pERK1/2 and ERK1/2 in CPCs. CPCs were loaded with EVs or Mock (PBS) for 0, 5, 10, 15, 30, and 60 min. The graphs show the ratios of pERK1/2 to ACTB (pERK/ACTB), ERK1/2 to ACTB (ERK/ACTB), and pERK1/2 to ERK1/2 (pERK/ERK) in CPCs based on immunoblotting. Data are presented as mean ± SD. The results are shown as the ratio at 0 min. The numbers indicate the average ratio to CPCs at 0 min. $n = 3$/group. Significance was determined using two-way ANOVA with Tukey's post hoc test; * $p < 0.05$, ns, no significance. # < 0.05 vs. 0 min. NS, not significant vs. 0 min.

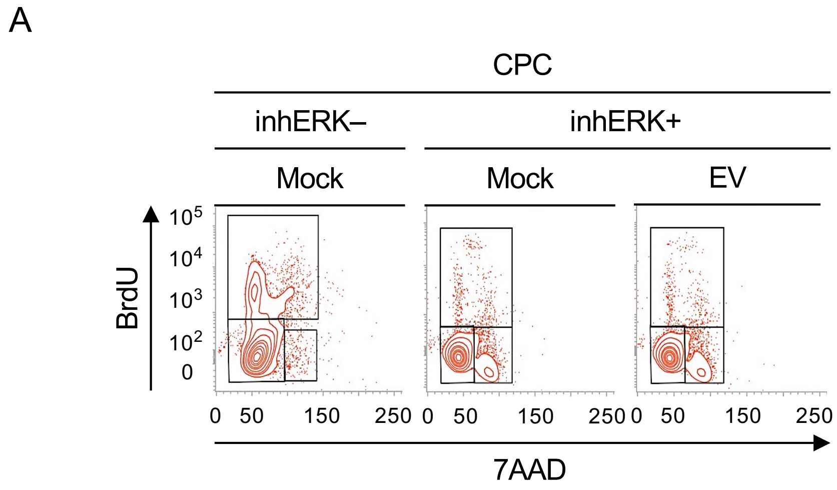

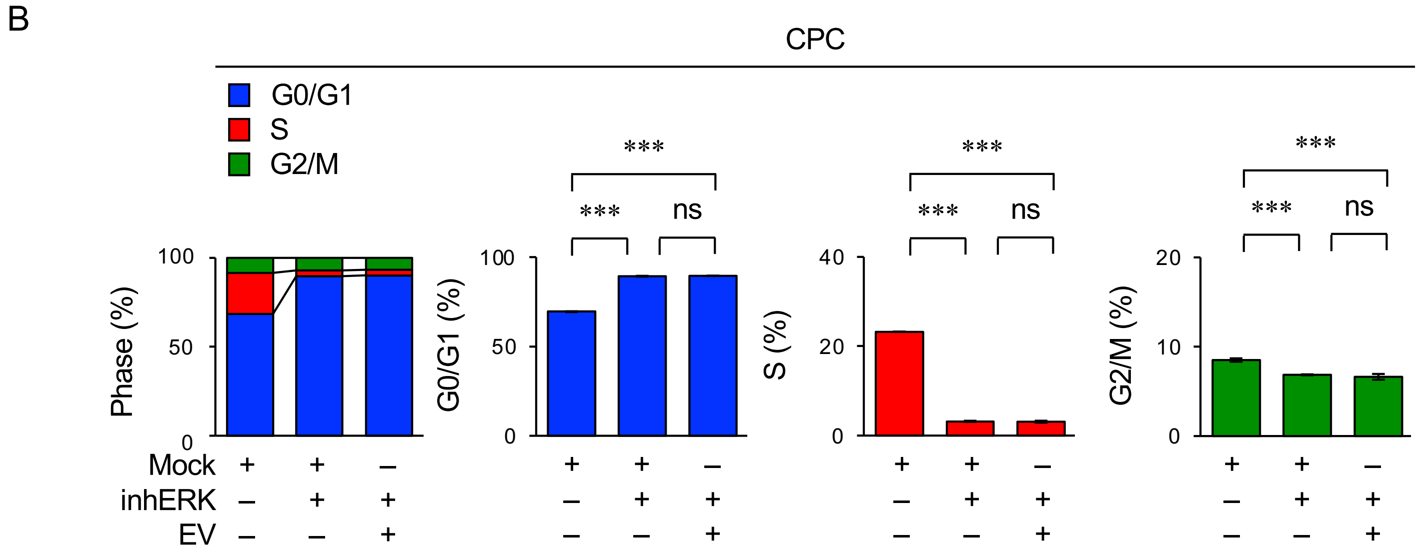

Supplementary Figure 9. Effects of ERK1/2 inhibitor on the cell cycle in CPCs under EV stimulation. CPCs were pretreated with an ERK1/2 inhibitor, PD98059 (inhERK; 10 μM) or Mock (PBS), for 1 day and subsequently treated with EVs (4 μg/mL). (A) Representative contour plots of the cell cycle using flow cytometry with BrdU-7AAD staining. (B) Graphs showing the ratio (%) of G0/G1, S, and G2/M phases. n = 3/group. Data are presented as mean ± SD. Significance was determined using a two-tailed t-test; ****P* < 0.005. ns, not significant.

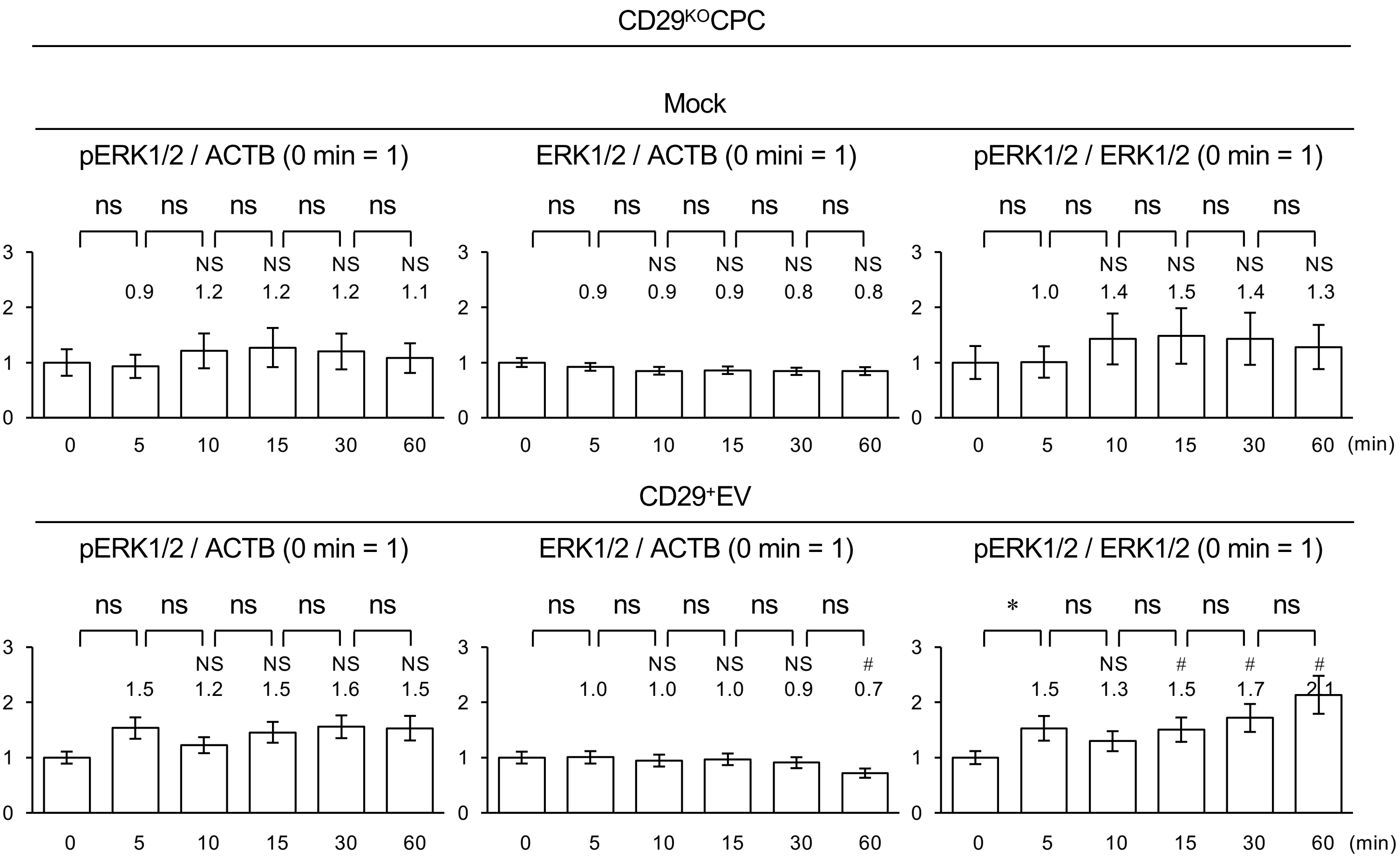

Supplementary Figure 10. Effects of CD29^{+}EVs on the expression of pERK1/2 and ERK1/2 in CD29^{KO}CPCs. CD29^{KO}CPCs were loaded with CD29^{+}EVs or Mock (PBS) for 0, 5, 10, 15, 30, and 60 min. The graphs show the ratios of pERK1/2 to ACTB (pERK/ACTB), ERK1/2 to ACTB (ERK/ACTB), and pERK1/2 to ERK1/2 (pERK/ERK) in CD29^{KO}CPCs based on immunoblotting. Data are presented as mean ± SD. The results are shown as the ratio at 0 min. The numbers indicate the average ratio to CD29^{KO}CPCs at 0 min. *n* = 3/group. Significance was determined using two-way ANOVA with Tukey's post hoc test; $*$ $p < 0.05$, ns, no significance. # < 0.05 vs. 0 min. NS, not significant vs. 0 min.

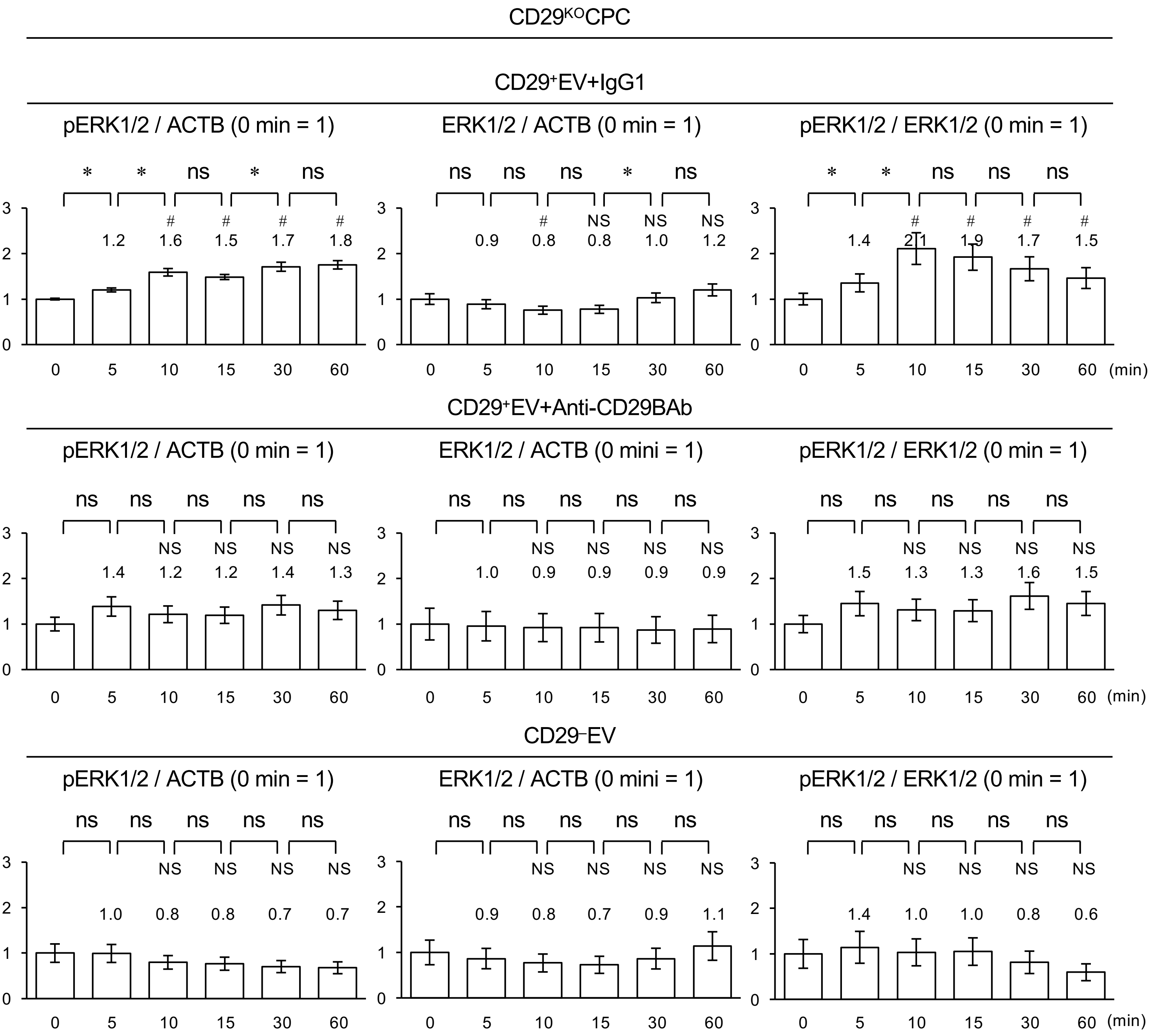

Supplementary Figure 11. Effects of anti-CD29 Bab and CD29^{-}EVs on the expression of pERK1/2 and ERK1/2 in CD29^{KO}CPCs. CD29^{KO}CPCs were loaded with CD29^{+}EVs in the presence of control IgG1 or anti-CD29 BAb for 0, 5, 10, 15, 30, and 60 min. CD29^{KO}CPCs were also loaded with CD29^{-}EVs for 0, 5, 10, 15, 30, and 60 min. The graphs show the ratios of pERK1/2 to ACTB (pERK/ACTB), ERK1/2 to ACTB (ERK/ACTB), and pERK1/2 to ERK1/2 (pERK/ERK) in CD29^{KO}CPCs based on immunoblotting. Data are presented as mean ± SD. The results are shown as the ratio at 0 min. The numbers indicate the average ratio to CD29^{KO}CPCs at 0 min. n = 3/group. Significance was determined using two-way ANOVA with Tukey's post hoc test; ∗ $p < 0.05$, ns, no significance. # < 0.05 vs. 0 min. NS, not significant vs. 0 min.

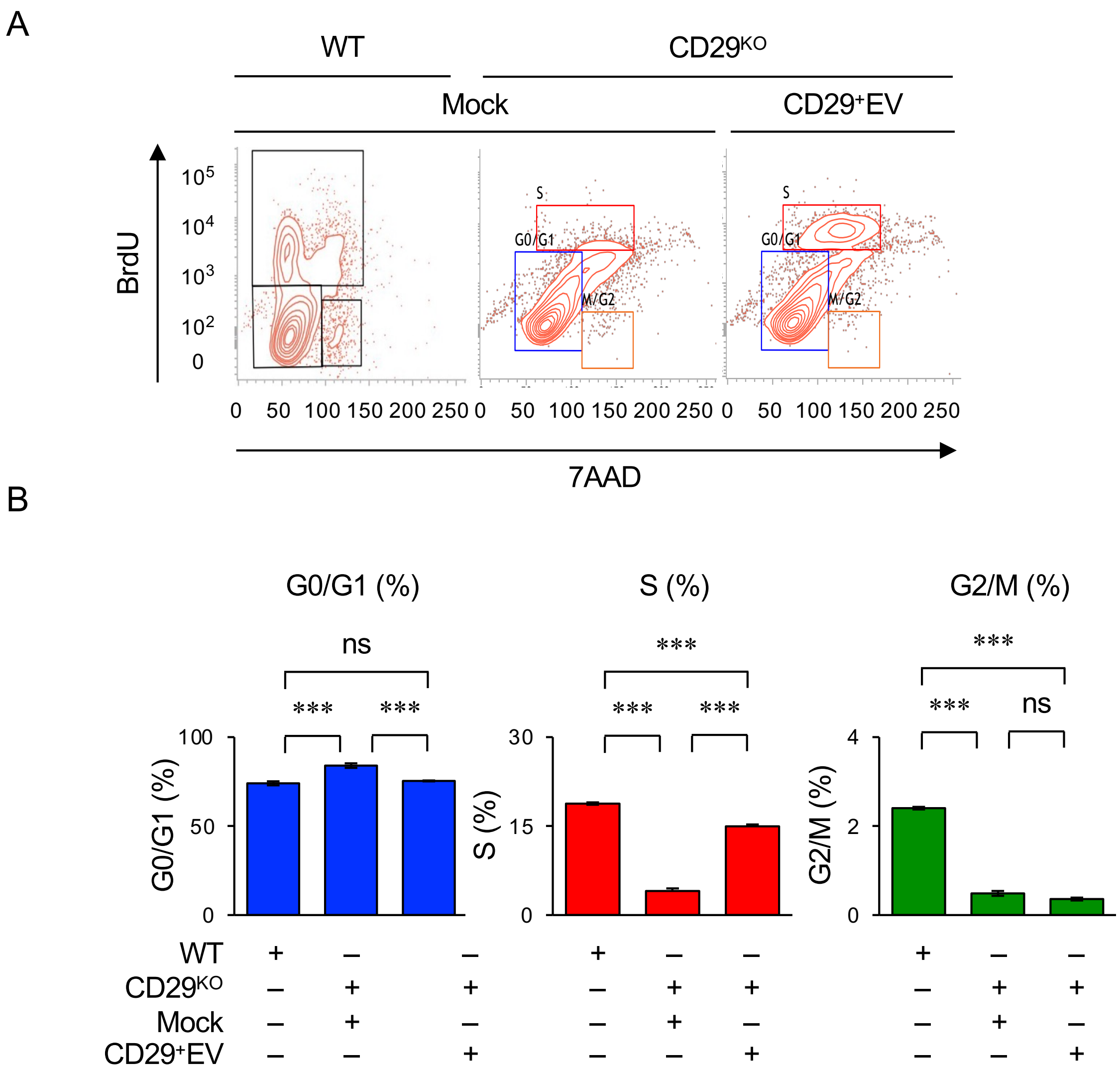

Supplementary Figure 12. Effects of EV-transferring CD29 on the cell cycle in CPCs under EV stimulation. CD29$^+$EVs (4 µg/mL) or PBS (Mock) were loaded on CD29^{KO}CPCs (CD29^{KO}) and wild-type CPC (WT). (A) Representative contour plots of the cell cycle in CD29^{KO}CPCs. (B) Graphs showing the ratio (%) of G0/G1, S, and G2/M phases. n = 3/group. Data are presented as mean ± SD. Significance was determined using a two-tailed t-test; ***$P < 0.005$. ns, not significant.

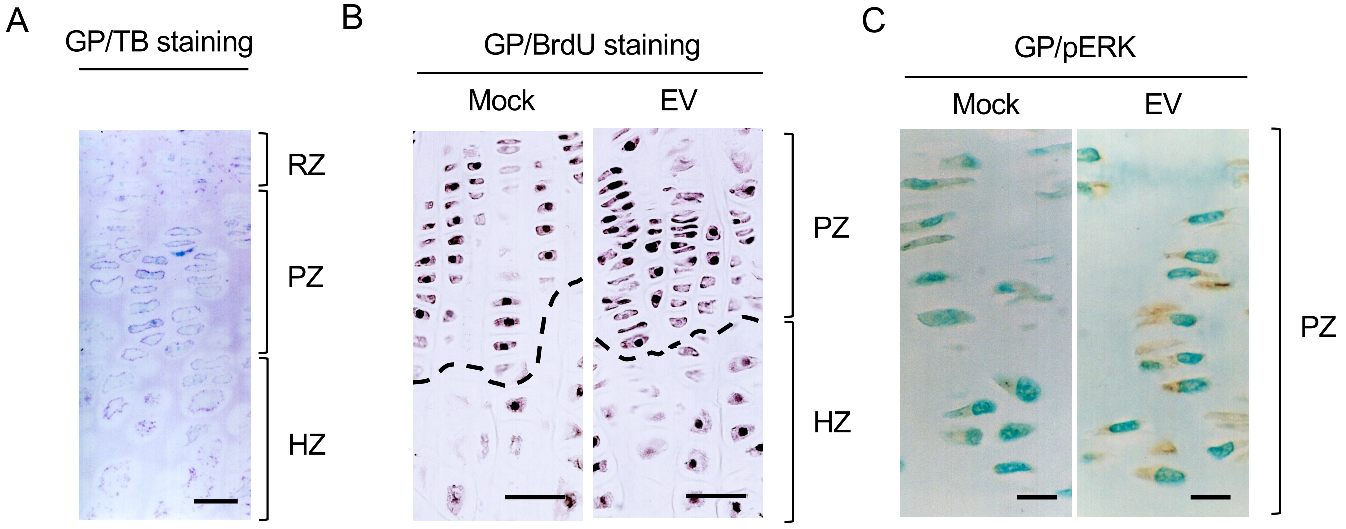

Supplementary Figure 13. *Ex vivo* effects of EVs on neonatal mouse growth plate cartilage. (A) A representative micrograph of the growth plate cartilage (GP) of the mesial epiphysis of tibiae of 2-week-old mice by toluidine blue (TB) staining. HZ, hypertrophic zone; PZ, proliferating zone; RZ, resting zone. (B) Representative micrographs of *ex vivo* mouse tibiae 5 days after EV loading using BrdU incorporation assay. Eosin counterstaining. Dot line, interface between PZ and HZ. (C) Representative immunohistochemical micrographs of pERK1/2 in PZ of GP. Methyl green counterstaining. Scale bar = 20 μm (A, B), 5 μm (C). (B, C) Mock, PBS-treated group; EV, EV-treated group.

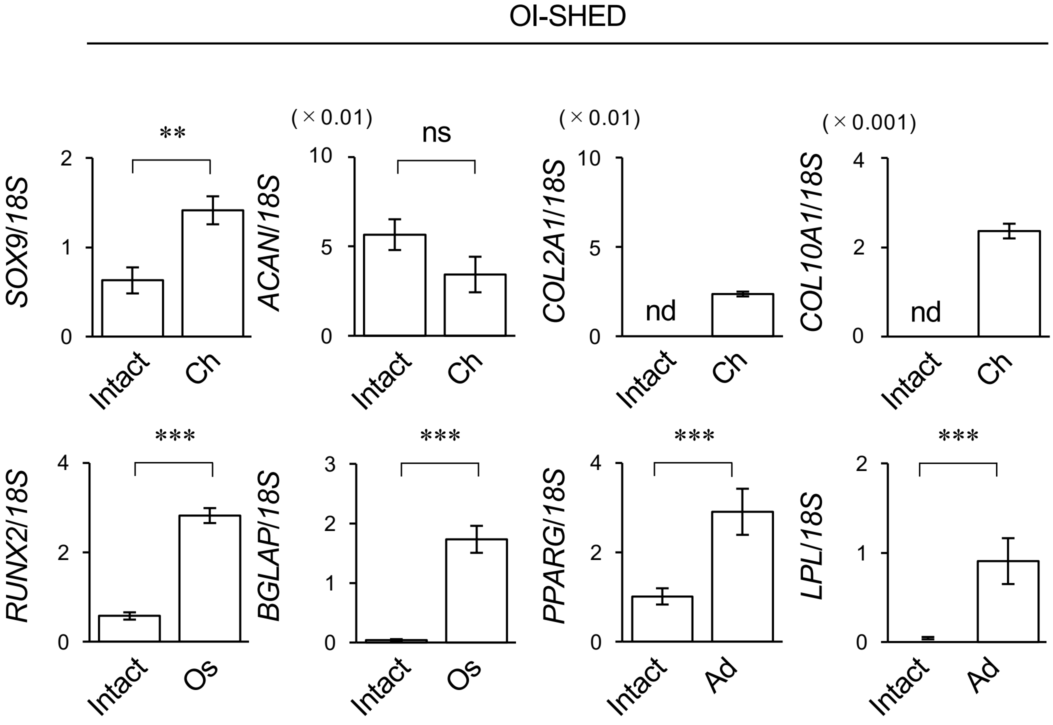

Supplementary Figure 14. Multipotency of OI-SHED. Graphs showing the results of expression of *SOX9, ACAN, COL2A1*, and *COL10A1* specific for chondrocytes, *RUNX2* and *BGLAP* specific for osteoblasts, and *PPARG* and *LPL* specific for adipocytes in CPCs using RT-qPCR. The results are analyzed as the ratio of 18S rRNA expression (*/18S*). Data are presented as mean ± SD. n = 2/group. Significance was determined using a two-tailed Student's *t* test; $^{**}P < 0.01$, $^{***}P < 0.005$. nd, not detected, ns, not significant. Ad, adipogenic condition; Ch, chondrogenic condition; Intact, uninduced condition; Os, osteogenic condition.

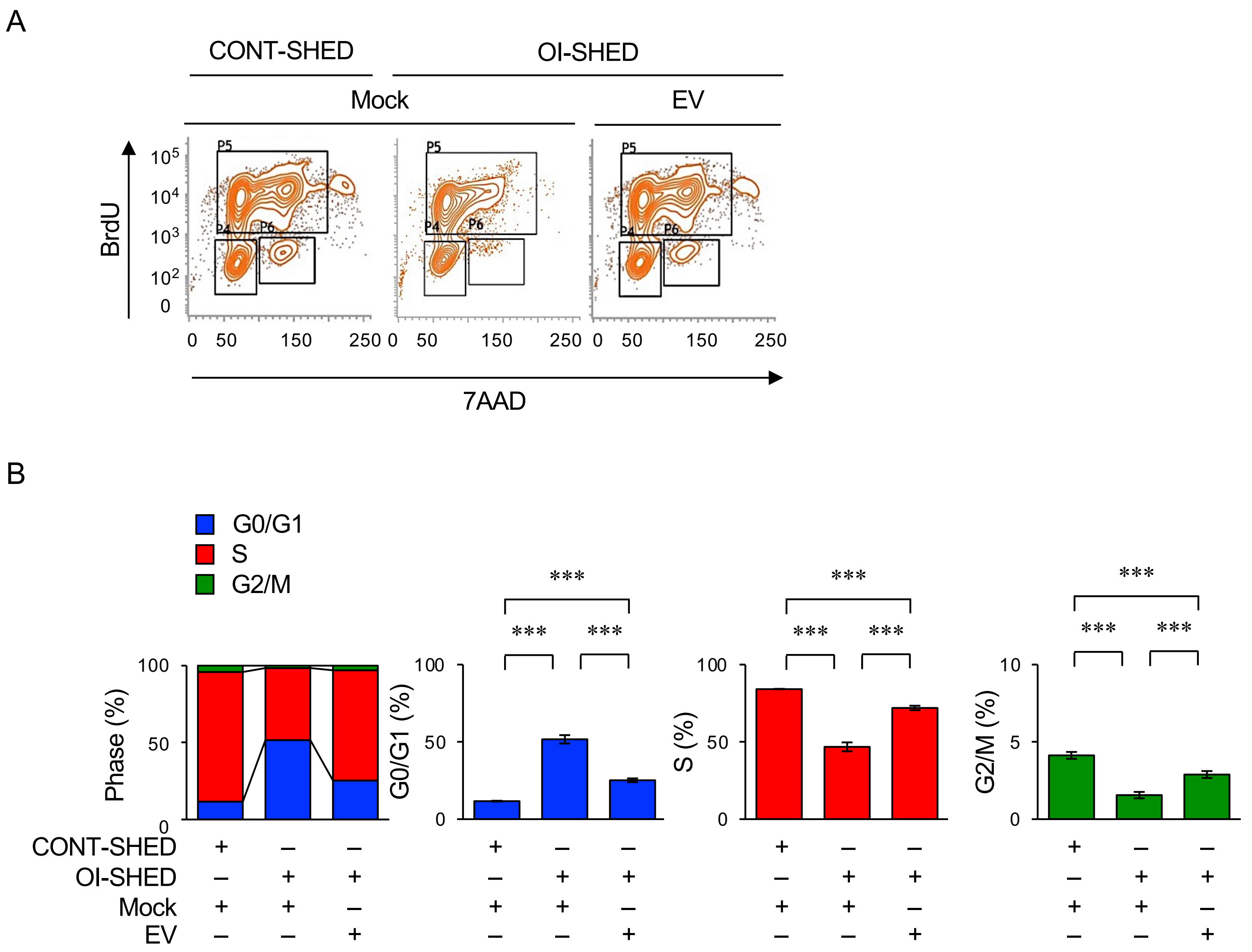

Supplementary Figure 15. Effects of EVs on cell cycle of OI-SHED. OI-SHED were treated with PBS (Mock) and EVs (4 μg/mL). (A). Representative contour plots of the cell cycle in OI-SHED using flow cytometry with BrdU-7AAD staining. (B) Graphs showing the percentage (%) of the G0/G1, S, and G2/M phases using flow cytometry. n = 3/group. Data are presented as mean ± SD. Significance was determined using a one-way ANOVA with Tukey's post-hoc test. *** $P < 0.005$. ns, not significant.

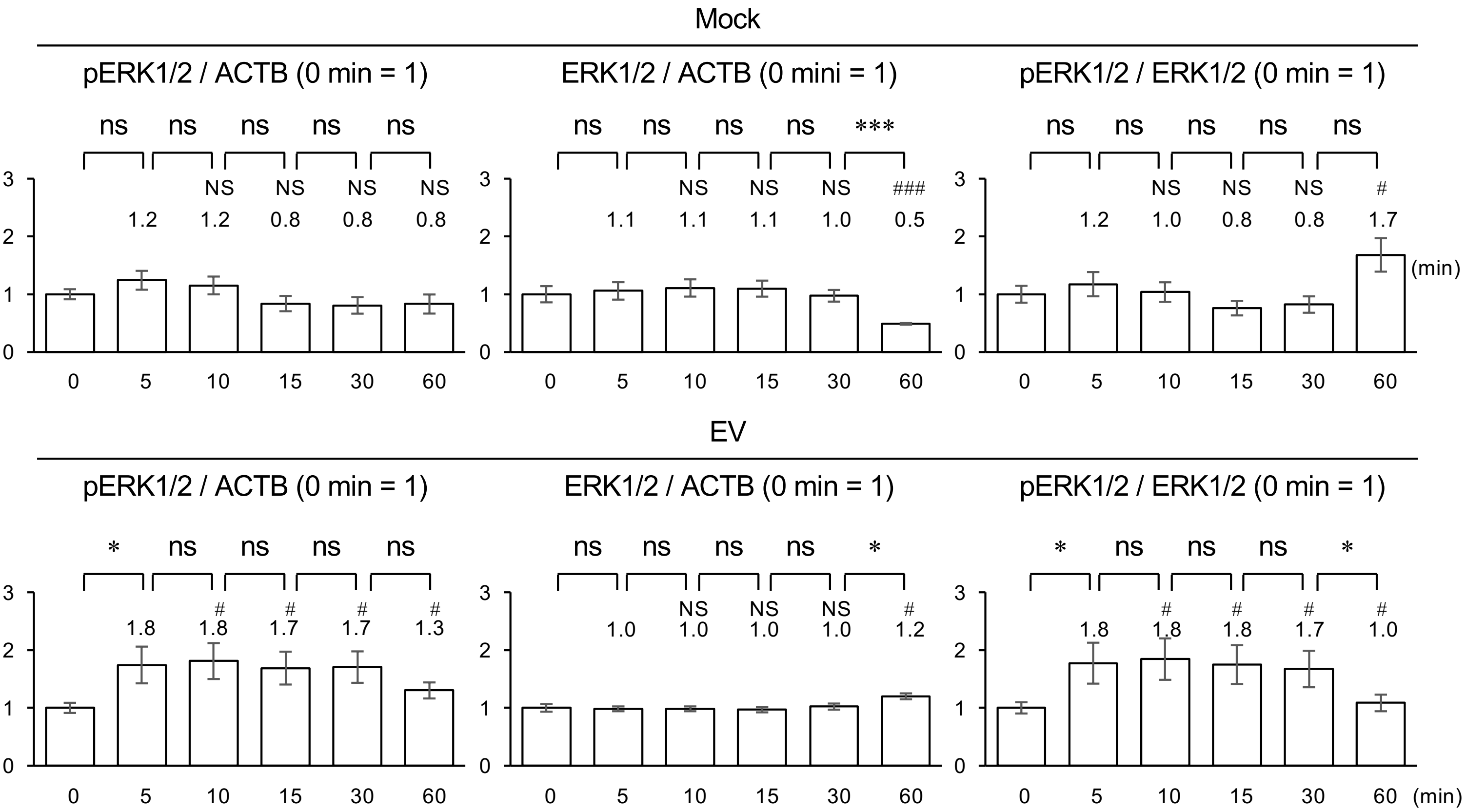

Supplementary Figure 16. Effects of EV on the expression of pERK1/2 and ERK1/2 in OI-SHED. OI-SHED were loaded with EVs or Mock (PBS) for 0, 5, 10, 15, 30, and 60 min. The graphs show the ratios of pERK1/2 to ACTB (pERK/ACTB), ERK1/2 to ACTB (ERK/ACTB), and pERK1/2 to ERK1/2 (pERK/ERK) in CPCs based on immunoblotting. Data are presented as mean ± SD. The results are shown as the ratio at 0 min. The numbers indicate the average ratio to OI-SHED at 0 min. *n* = 3/group. Significance was determined using two-way ANOVA with Tukey's post hoc test; * $p < 0.05$, ** $p < 0.01$, and *** $p < 0.001$. ns, no significance. # < 0.05 vs. 0 min; ### < 0.005 vs. 0 min. NS, not significant vs. 0 min.

Uncropped full-length images for western blot analysis

Figure 2C

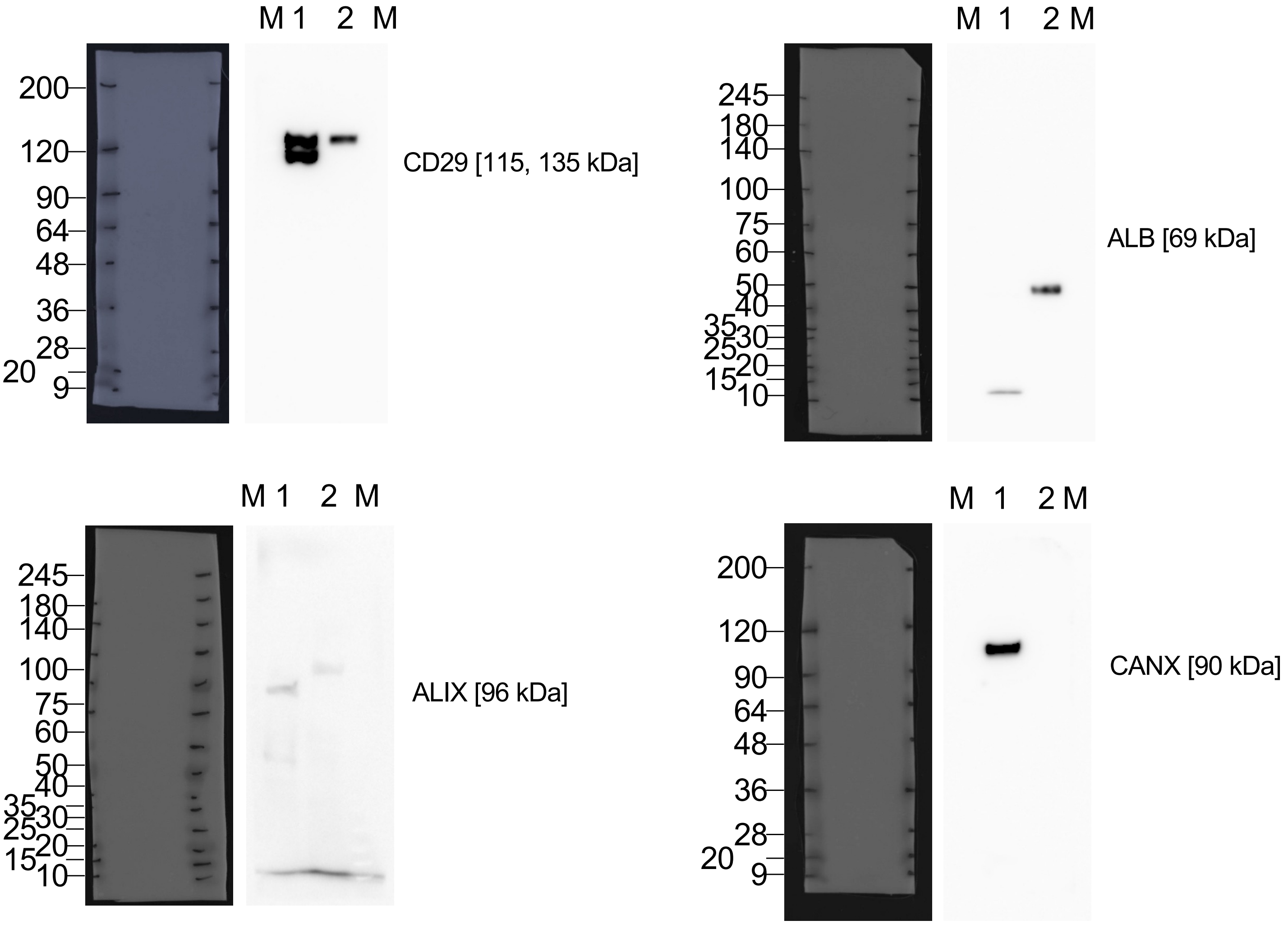

M: Molecular Markers
1: SHED
2: EV
M: Molecular Markers

Figure 2G

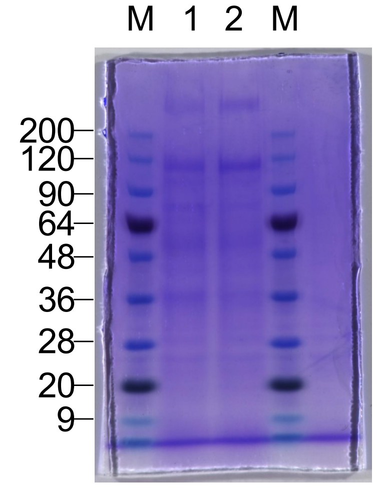

M: Molecular Markers
2: Untreated EV
3: RNase-EV
M: Molecular Markers

Figure 4A

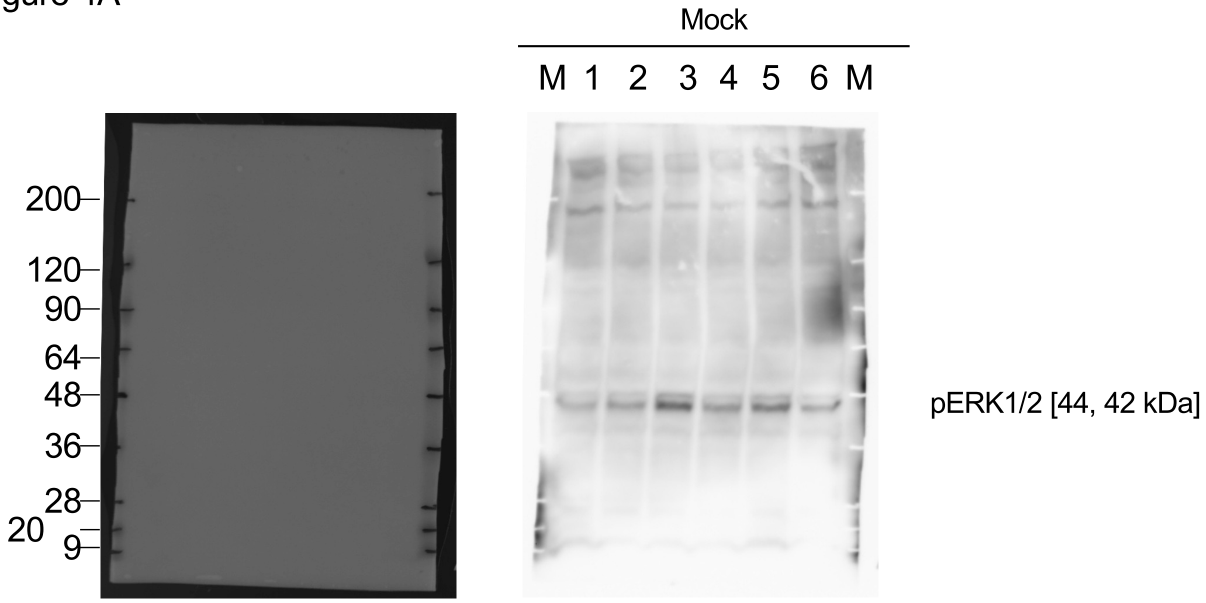

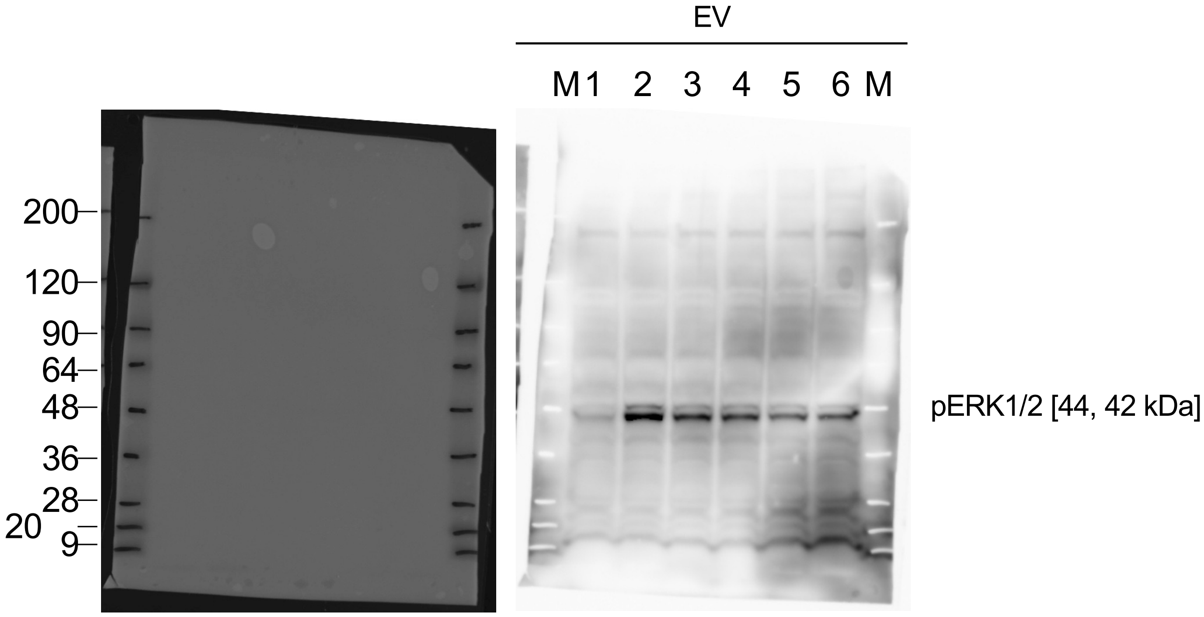

M: molecular marker
1: 0 min
2: 5 min
3: 10 min
4: 15 min
5: 30 min
6: 60 min
M: molecular marker

Figure 4A

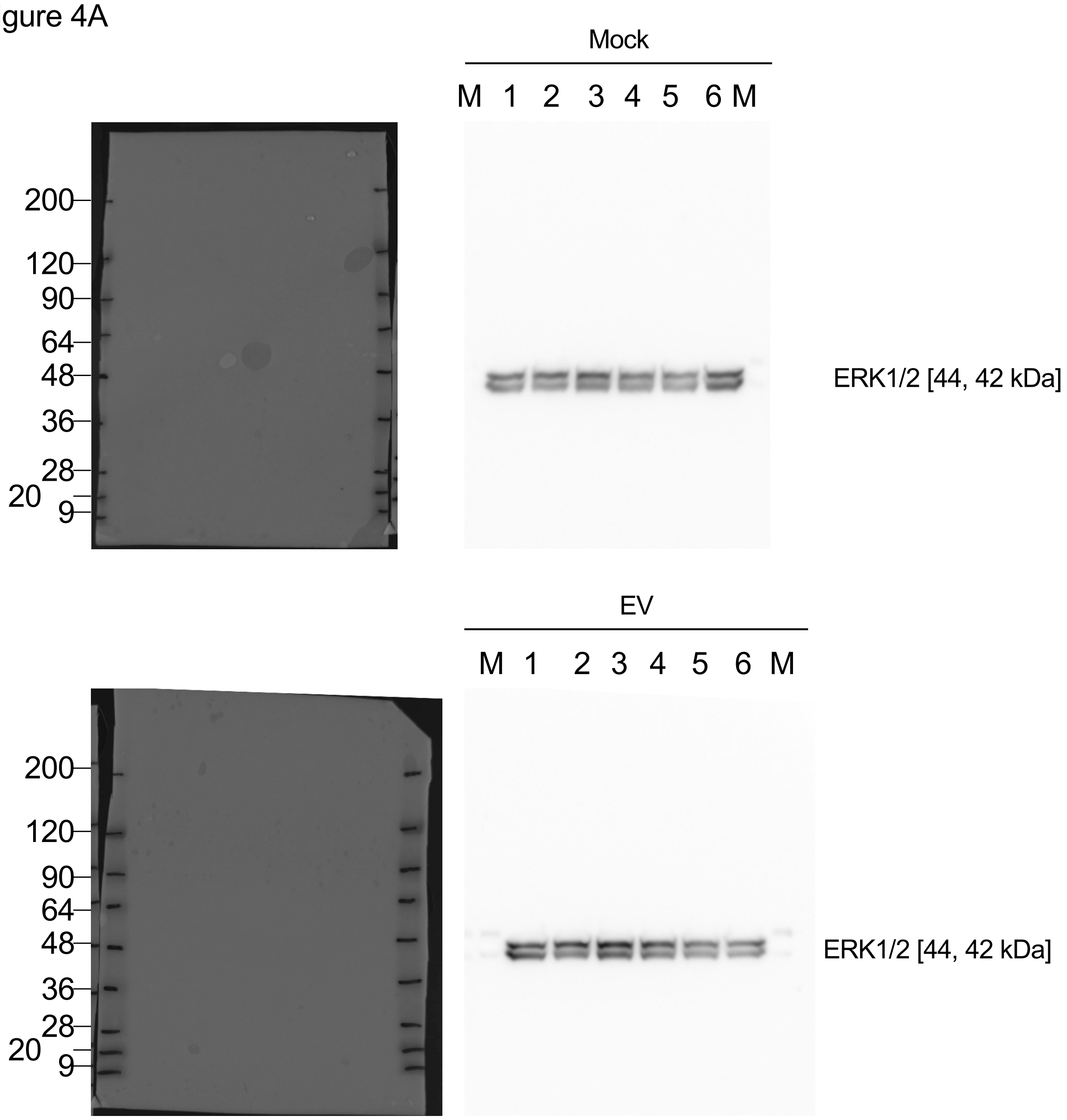

M: molecular marker
1: 0 min
2: 5 min
3: 10 min
4: 15 min
5: 30 min
6: 60 min
M: molecular marker

Figure 4A

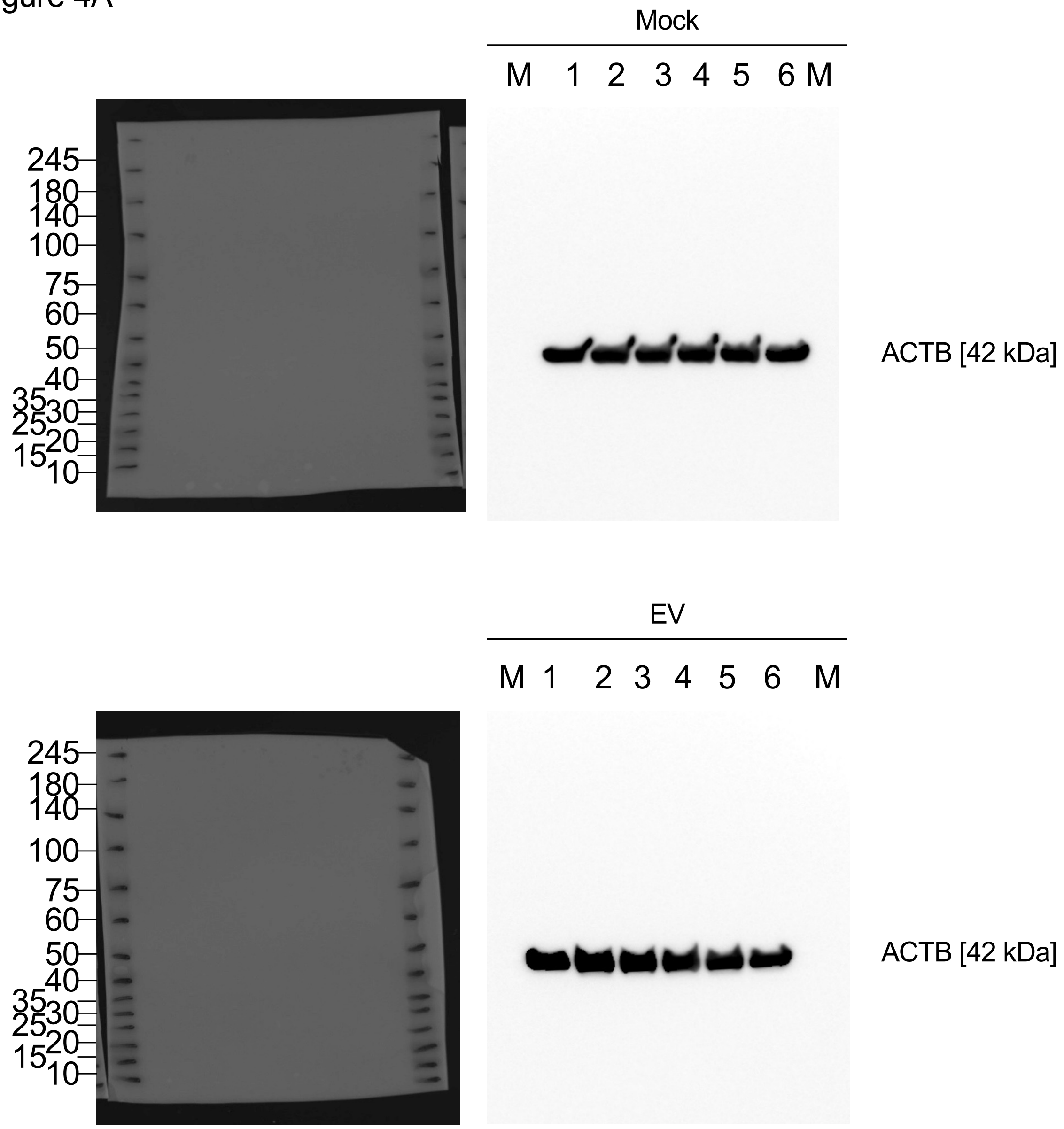

M: molecular marker
1: 0 min
2: 5 min
3: 10 min
4: 15 min
5: 30 min
6: 60 min
M: molecular marker

Figure 5A

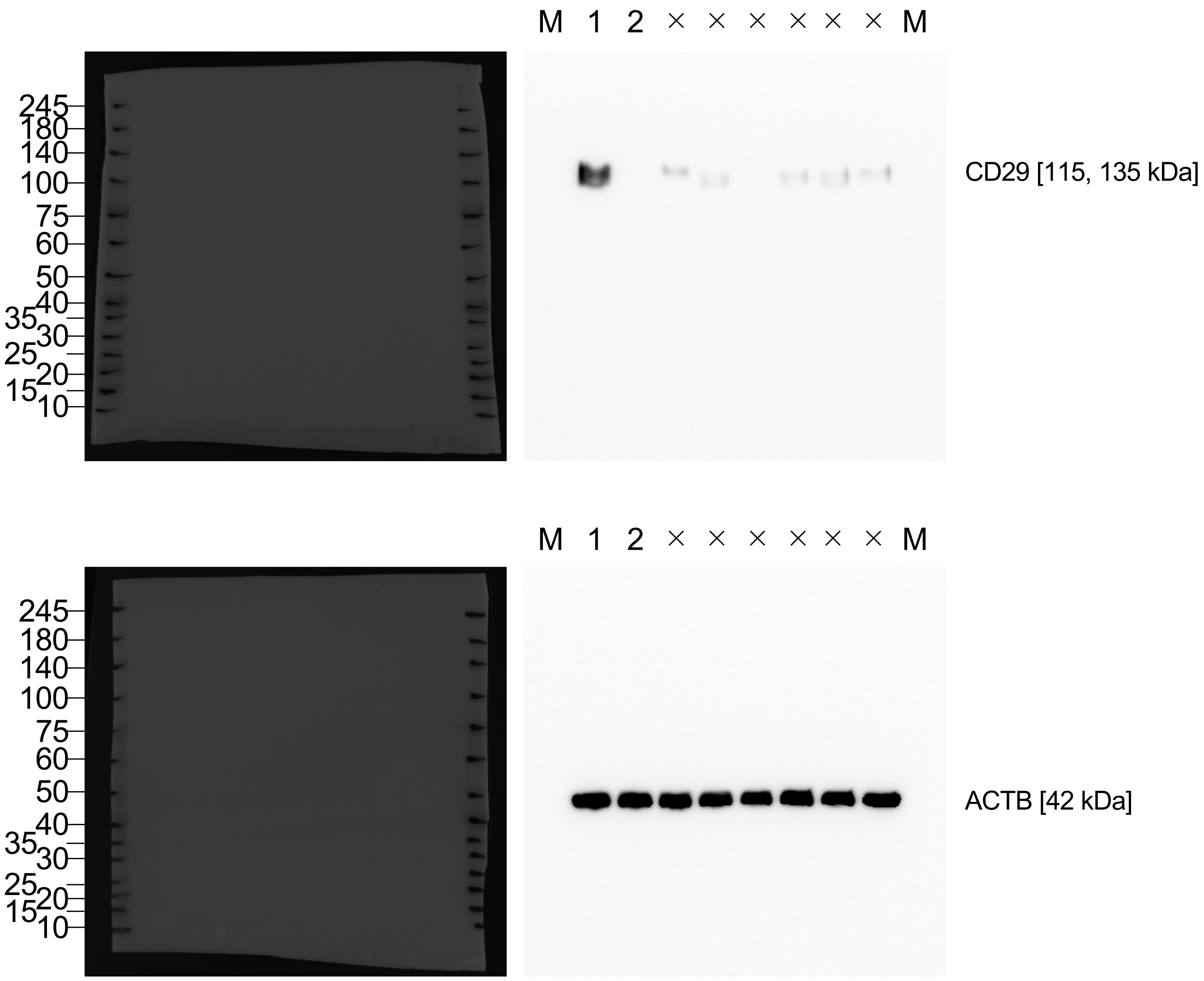

M: Molecular Markers
1: WT
2: $CD29^{KO}$
M: Molecular Markers

Figure 5E

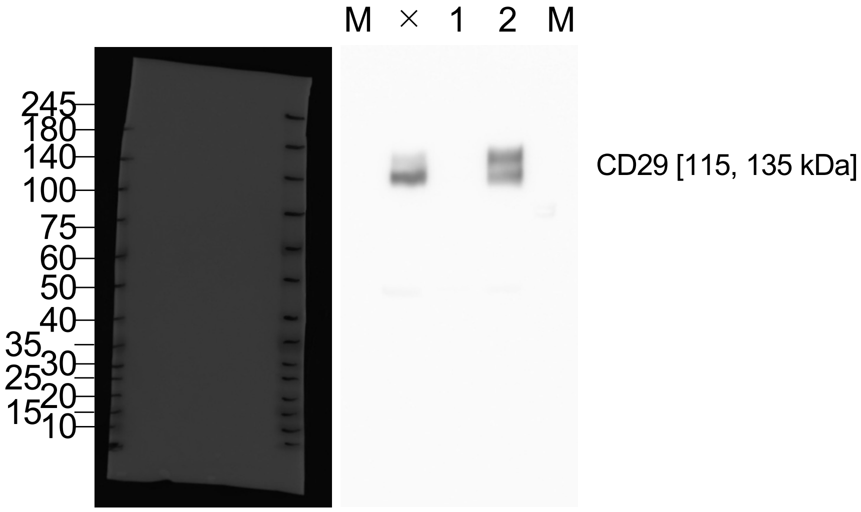

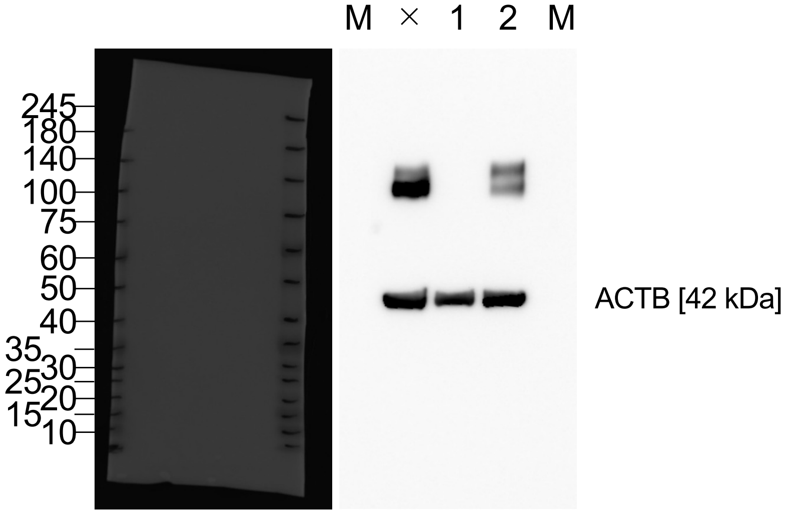

M: Molecular Markers
1: CD29⁻
2: CD29⁺
M: Molecular Markers

Figure 5F

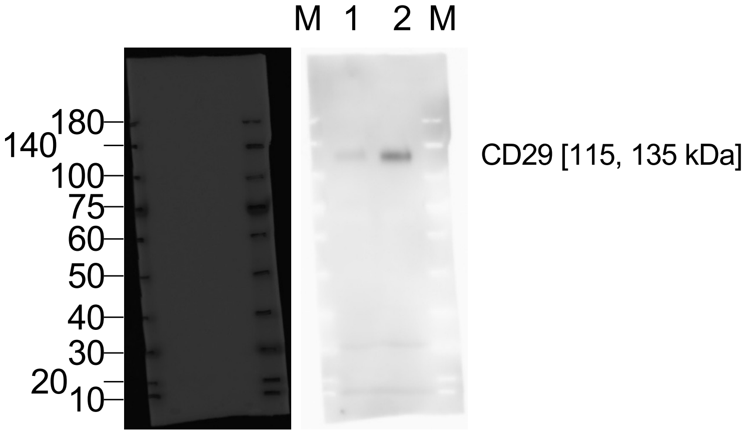

M: Molecular Markers
1: CD29⁻EV
2: CD29⁺EV
M: Molecular Markers

Figure 6B

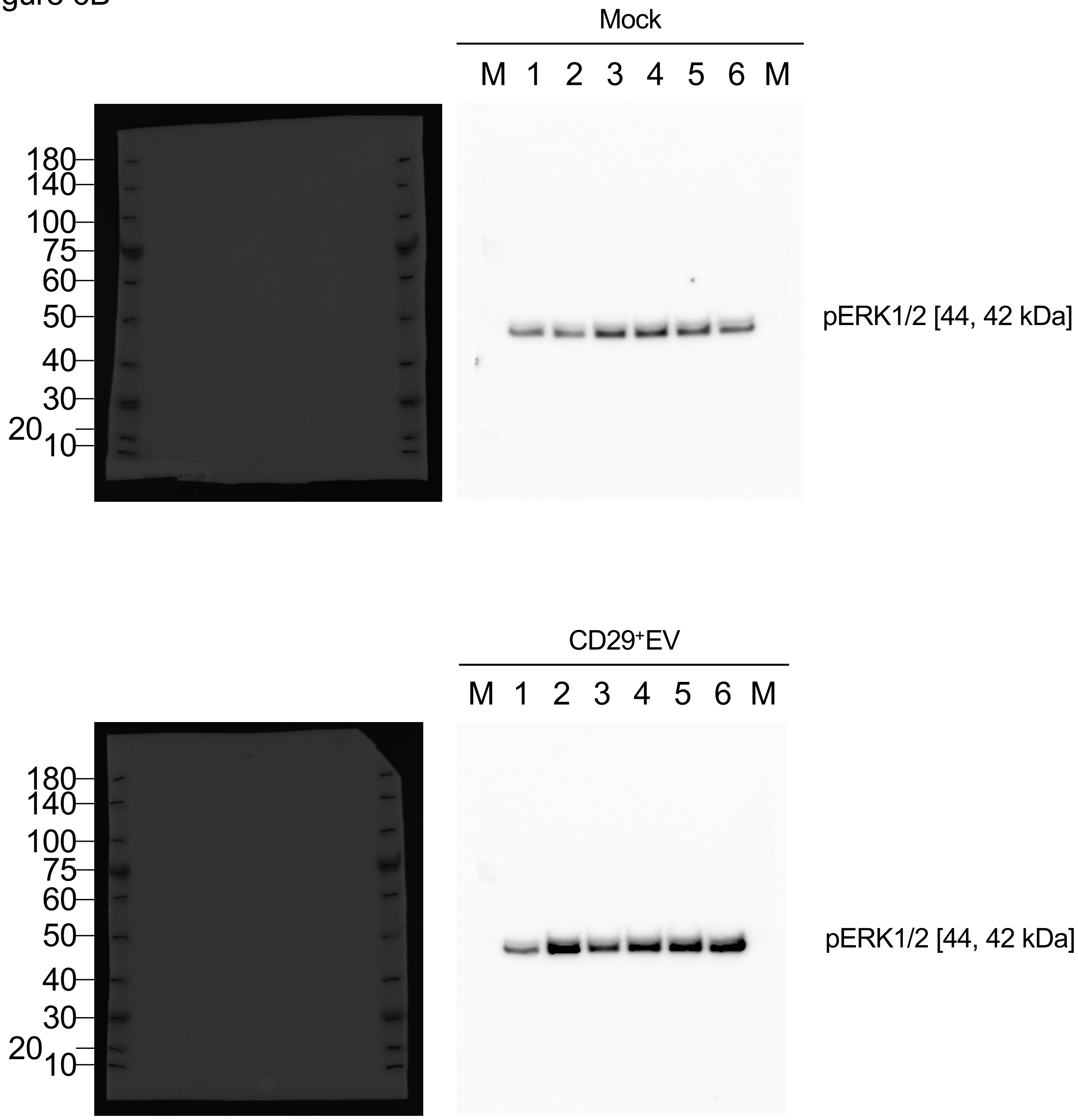

M: molecular marker
1: 0 min
2: 5 min
3: 10 min
4: 15 min
5: 30 min
6: 60 min
M: molecular marker

Figure 6B

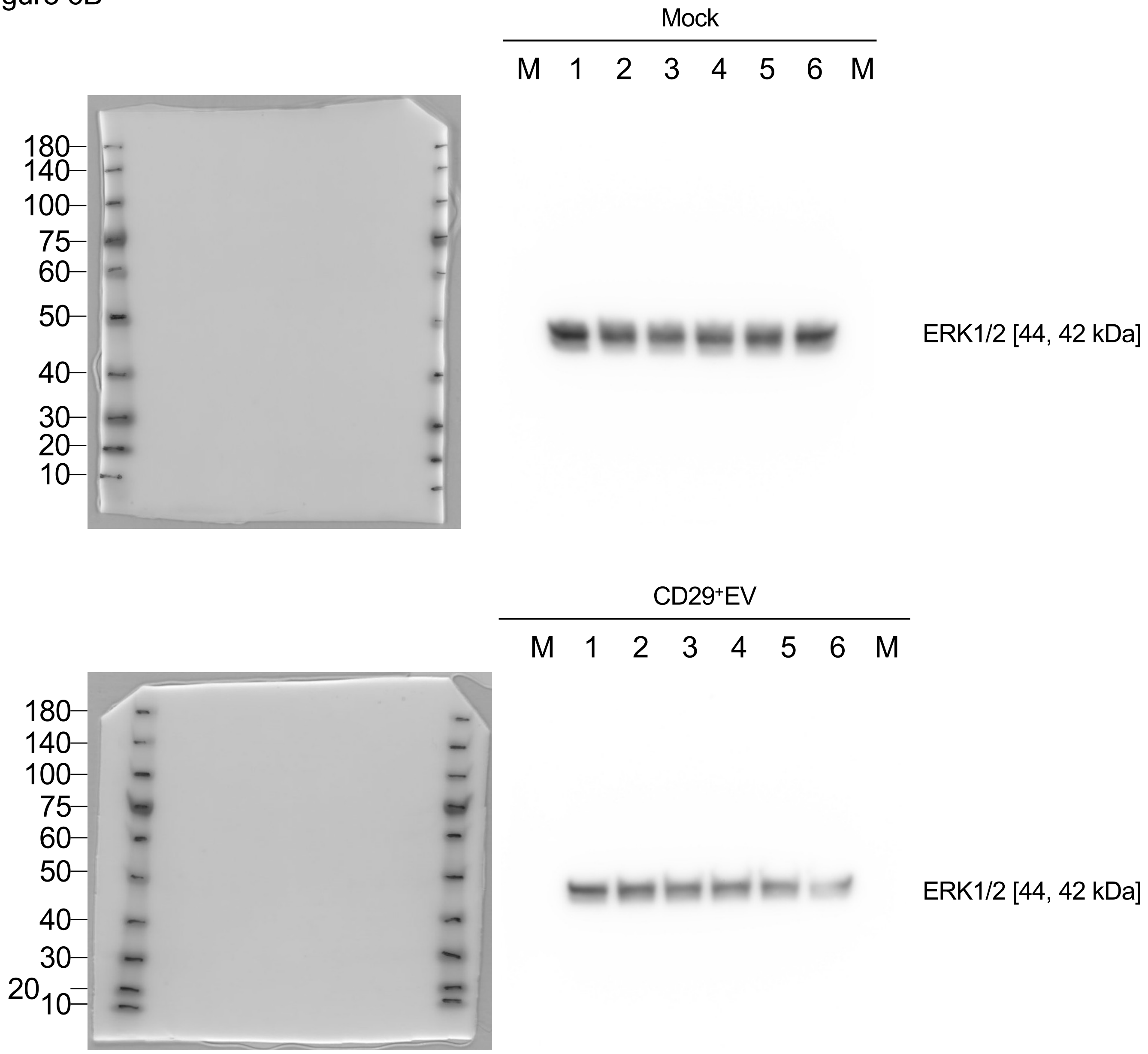

M: molecular marker
1: 0 min
2: 5 min
3: 10 min
4: 15 min
5: 30 min
6: 60 min
M: molecular marker

Figure 6B

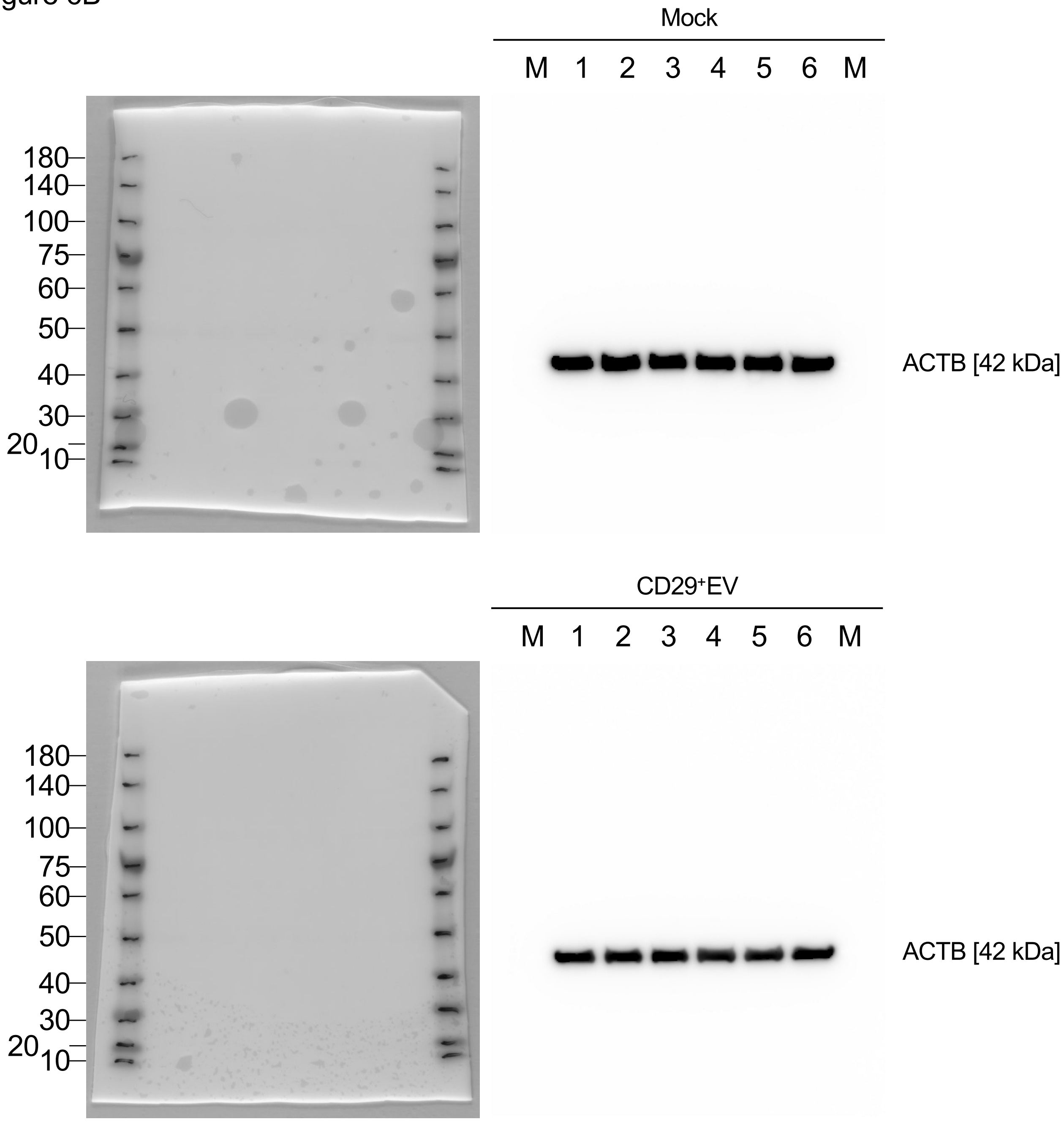

M: molecular marker
1: 0 min
2: 5 min
3: 10 min
4: 15 min
5: 30 min
6: 60 min
M: molecular marker

Figure 6C

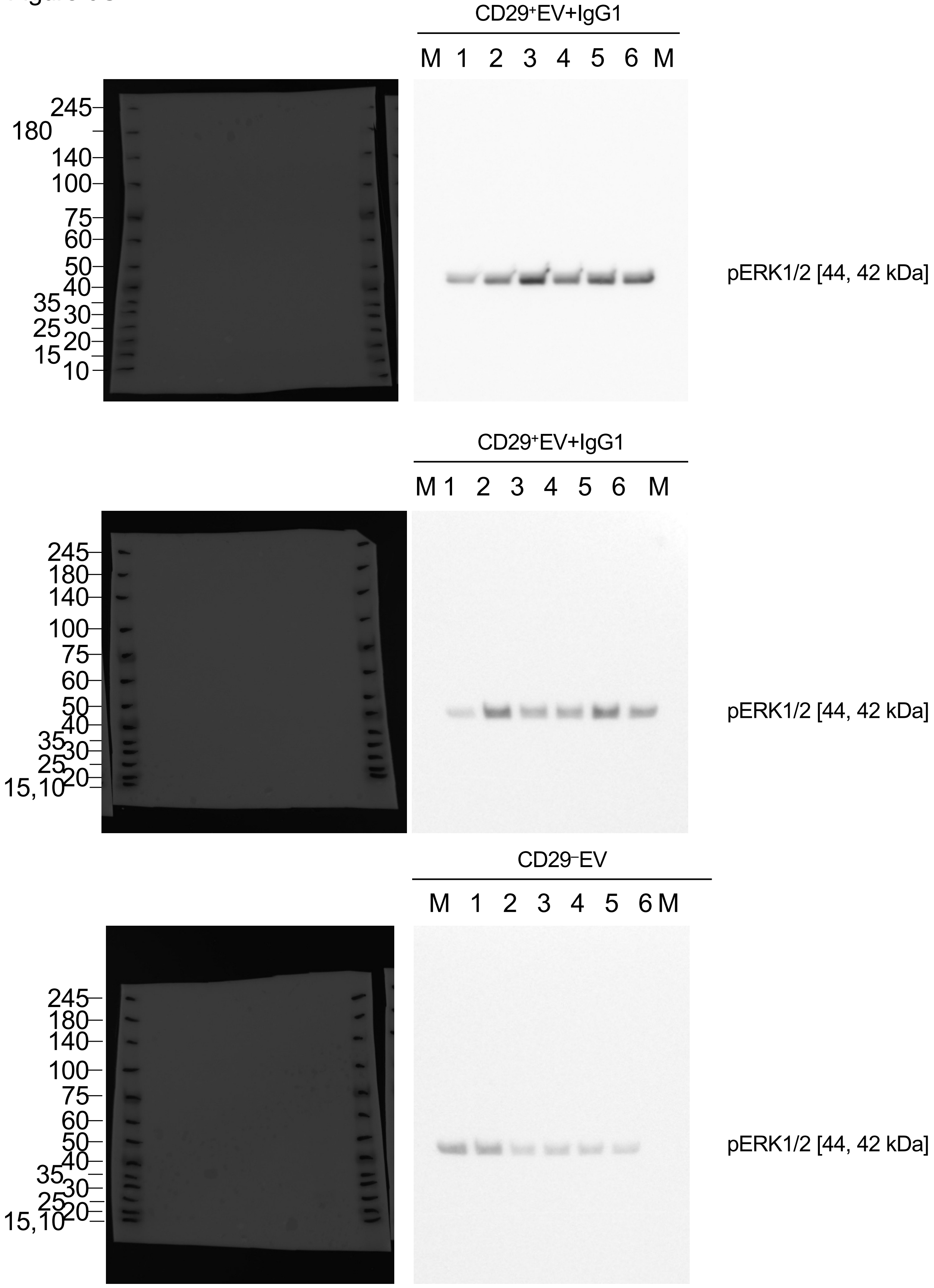

M: molecular marker
1: 0 min
2: 5 min
3: 10 min
4: 15 min
5: 30 min
6: 60 min
M: molecular marker

Figure 6C

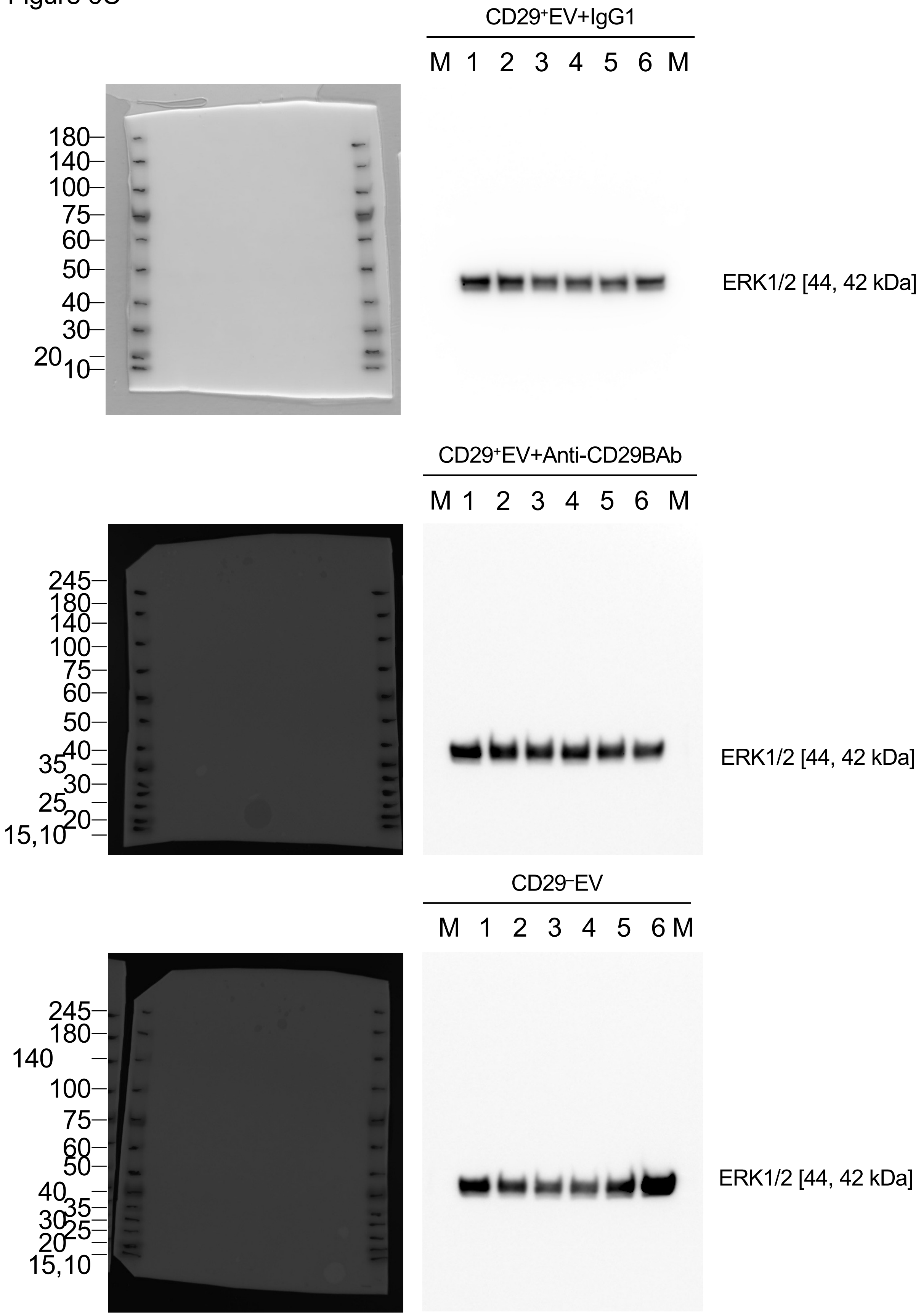

M: molecular marker
1: 0 min
2: 5 min
3: 10 min
4: 15 min
5: 30 min
6: 60 min
M: molecular marker

Figure 6C

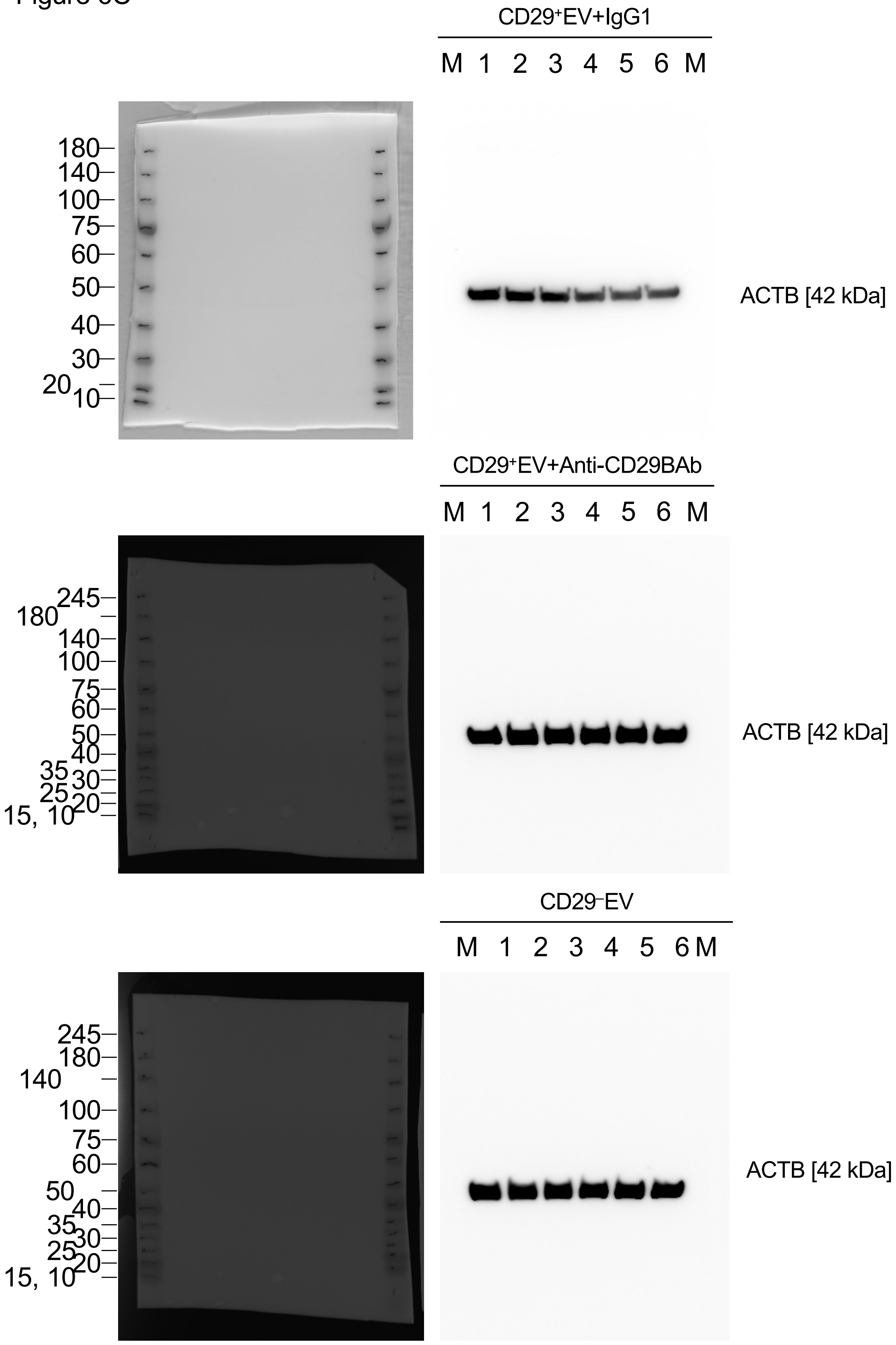

M: molecular marker
1: 0 min
2: 5 min
3: 10 min
4: 15 min
5: 30 min
6: 60 min
M: molecular marker

Figure 8C

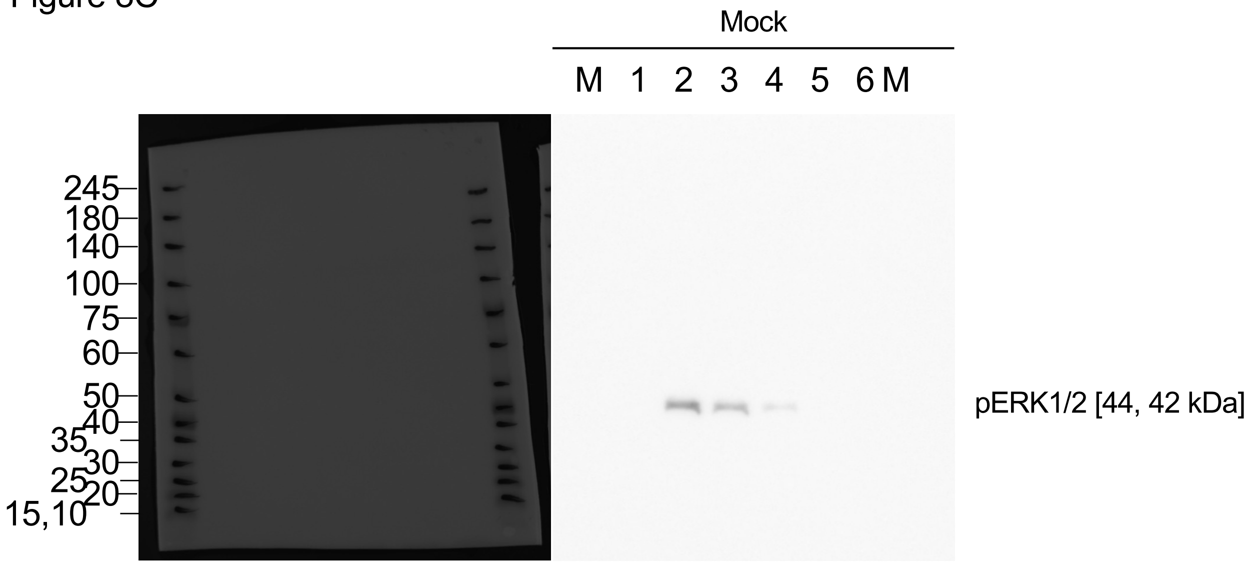

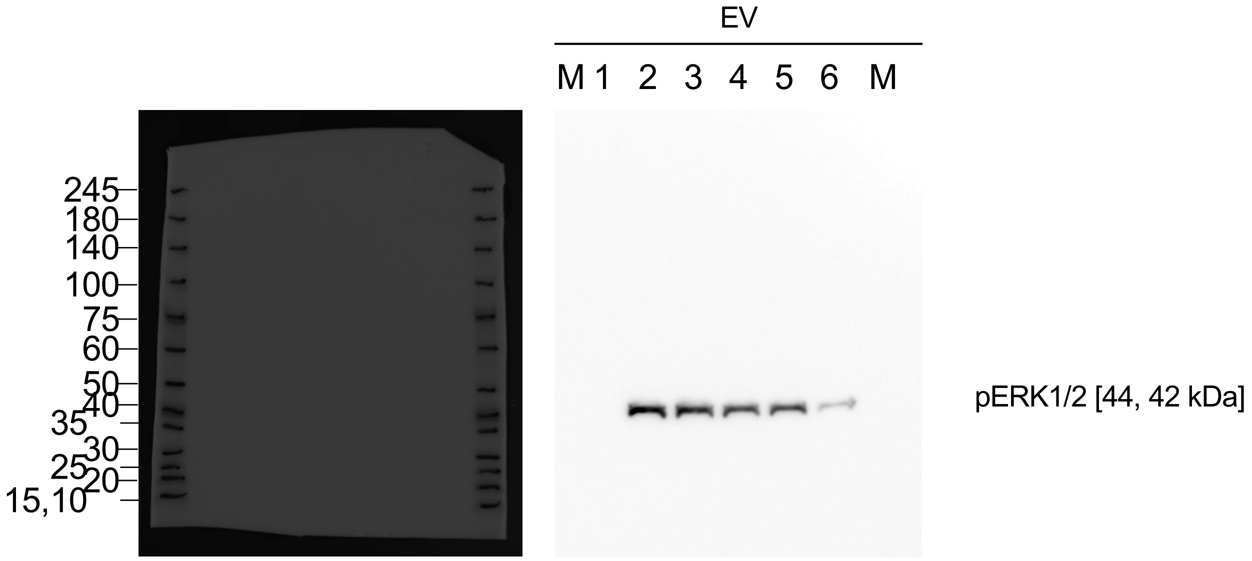

M: molecular marker
1: 0 min
2: 5 min
3: 10 min
4: 15 min
5: 30 min
6: 60 min
M: molecular marker

Figure 8C

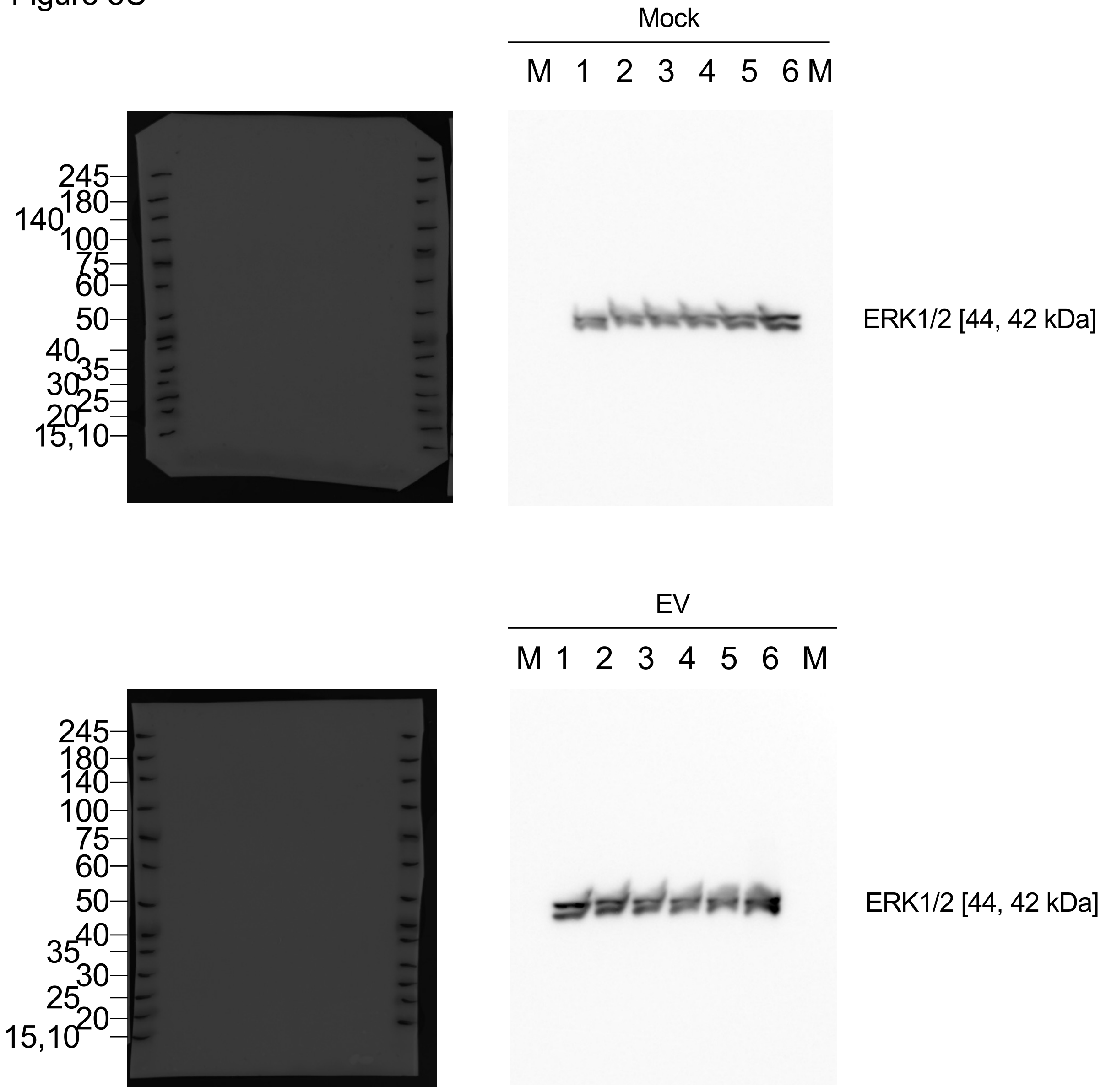

M: molecular marker
1: 0 min
2: 5 min
3: 10 min
4: 15 min
5: 30 min
6: 60 min
M: molecular marker

Figure 8C

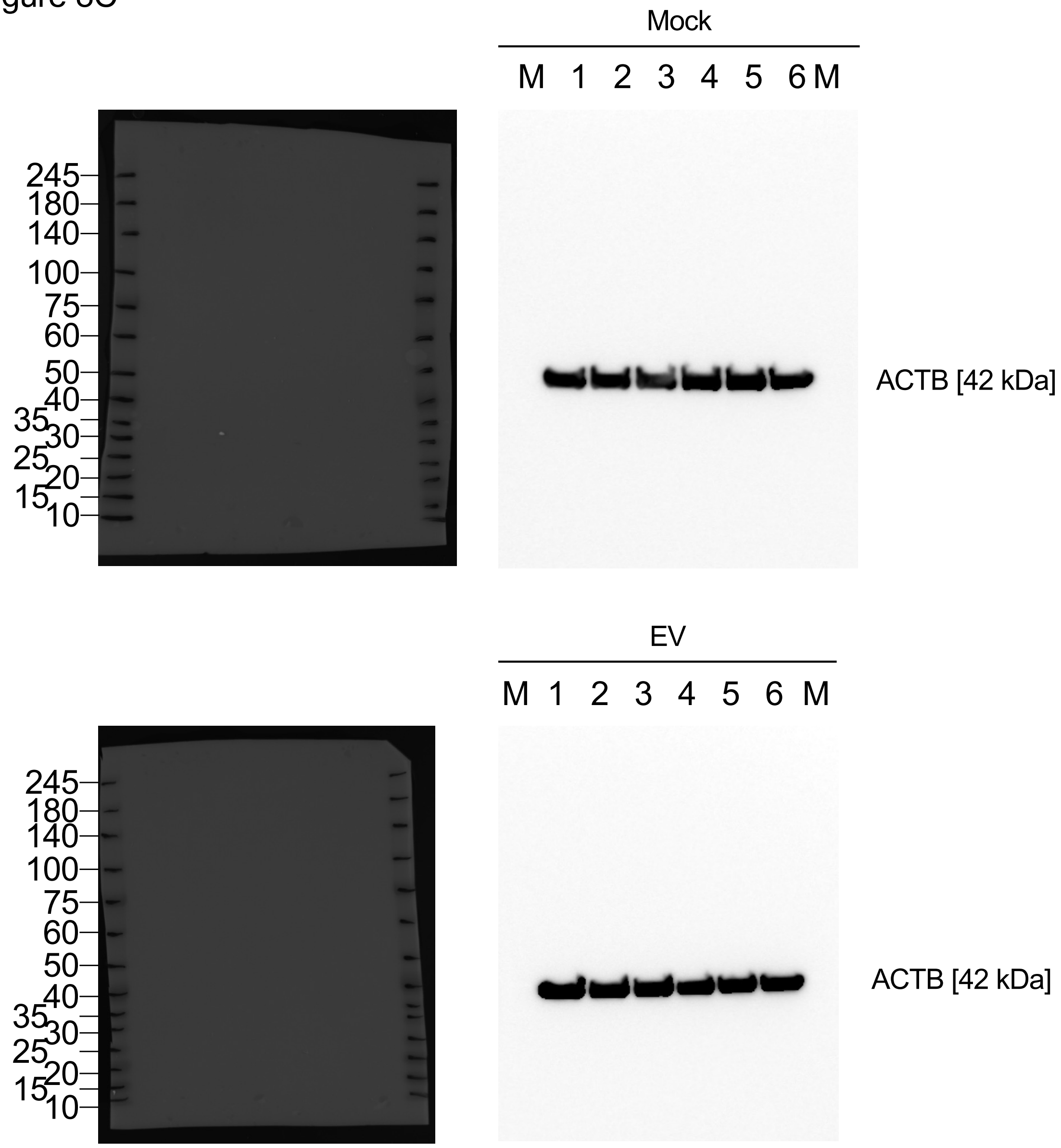

M: molecular marker
1: 0 min
2: 5 min
3: 10 min
4: 15 min
5: 30 min
6: 60 min
M: molecular marker
